# Longitudinal phenotypes in patients with acute respiratory distress syndrome: a multi-database study

**DOI:** 10.1186/s13054-022-04211-w

**Published:** 2022-11-04

**Authors:** Hui Chen, Qian Yu, Jianfeng Xie, Songqiao Liu, Chun Pan, Ling Liu, Yingzi Huang, Fengmei Guo, Haibo Qiu, Yi Yang

**Affiliations:** 1 Jiangsu Provincial Key Laboratory of Critical Care Medicine, Department of Critical Care Medicine, Zhongda Hospital, School of Medicine, Southeast University, No. 87, Dingjiaqiao Road, Gulou District, Nanjing, 210009 People’s Republic of China; 2 Department of Critical Care Medicine, The First Affiliated Hospital of Soochow University, Soochow University, No. 899 Pinghai Road, Suzhou, 215000 People’s Republic of China; 3 Department of Radiology, Zhongda Hospital, School of Medicine, Southeast University, No. 87, Dingjiaqiao Road, Gulou District, Nanjing, 210009 People’s Republic of China

**Keywords:** Acute respiratory distress syndrome, Longitudinal phenotypes, 28-day mortality, Heterogeneity of treatment effect

## Abstract

**Background:**

Previously identified phenotypes of acute respiratory distress syndrome (ARDS) have been limited by a disregard for temporal dynamics. We aimed to identify longitudinal phenotypes in ARDS to test the prognostic and predictive enrichment of longitudinal phenotypes, and to develop simplified models for phenotype identification.

**Methods:**

We conducted a multi-database study based on the Chinese Database in Intensive Care (CDIC) and four ARDS randomized clinical trials (RCTs). We employed latent class analysis (LCA) to identify longitudinal phenotypes using 24-hourly data from the first four days of invasive ventilation. We used the Cox regression model to explore the association between time-varying respiratory parameters and 28-day mortality across phenotypes. Phenotypes were validated in four RCTs, and the heterogeneity of treatment effect (HTE) was investigated. We also constructed two multinomial logistical regression analyses to develop the probabilistic models.

**Findings:**

A total of 605 ARDS patients in CDIC were enrolled. The three-class LCA model was identified and had the optimal fit, as follows: Class 1 (*n* = 400, 66.1% of the cohort) was the largest phenotype over all study days, and had fewer abnormal values, less organ dysfunction and the lowest 28-day mortality rate (30.5%). Class 2 (*n* = 102, 16.9% of the cohort) was characterized by pulmonary mechanical dysfunction and had the highest proportion of poorly aerated lung volume, the 28-day mortality rate was 47.1%. Class 3 (*n* = 103, 17% of the cohort) was correlated with extra-pulmonary dysfunction and had the highest 28-day mortality rate (56.3%). Time-varying mechanical power was more significantly associated with 28-day mortality in Class 2 patients compared to other phenotypes. Similar phenotypes were identified in four RCTs. A significant HTE between phenotypes and treatment strategies was observed in the ALVEOLI (high PEEP vs. low PEEP) and the FACTT trials (conservative vs. liberal fluid management). Two parsimonious probabilistic models were constructed to identify longitudinal phenotypes.

**Interpretation:**

We identified and validated three novel longitudinal phenotypes for ARDS patients, with both prognostic and predictive enrichment. The phenotypes of ARDS can be accurately identified with simple classifier models, except for Class 3.

**Supplementary Information:**

The online version contains supplementary material available at 10.1186/s13054-022-04211-w.

## Introduction

Acute respiratory distress syndrome (ARDS), clinically defined by the Berlin definition [1], is a heterogenous syndrome characterized by acute hypoxic respiratory failure that can be caused by a wide variety of insults [2]. ARDS is a clinically heterogenous syndrome with diverse populations, multiple etiologies, and a broad definition which might explain the absence of benefit in most randomized controlled trials (RCTs) assessing various treatment strategies [3]. Identifying specific ARDS phenotypes could lead to more favorable clinical trials and personalized ARDS management [4–6].

Several ARDS phenotypes have been documented. In the past few years, Calfee and colleagues used latent class analysis (LCA) using cross-sectional data at baseline and identified two phenotypes: a hypo-inflammatory, and a hyper-inflammatory phenotype [7]. The latter had higher levels of pro-inflammatory biomarkers and poorer outcomes. Subsequent analyses demonstrated that patients with the hyper-inflammatory phenotype might benefit more from higher positive end-expiratory pressure (PEEP) and a conservative fluid strategy [8, 9]. According to 54 respiratory and CT-derived variables, a two-class model was identified as best fitting: non-recruitable phenotype and recruitable phenotype, and the recruitable phenotype presented with an increased PaO_2_/FiO_2_ ratio, compliance, and decreased alveolar dead space in response to a ﻿standardized recruitment maneuver [10].

However, these phenotypes do not capture the complexity and diversity of ARDS, and were derived only based on ﻿cross-sectional data collected within one day. Using more diverse data with a longitudinal approach might be more informative in identifying phenotypes. The longitudinal phenotypes of traditional ARDS have never been addressed. Phenotypes may be dynamic and change throughout the course of a patient’s illness. A retrospective study divided septic patients into four illness categories based on the severity of laboratory and vital sign abnormalities, and demonstrated that almost 60% of them changed their illness category at least once during hospitalization [11]. To our best knowledge, only one prior study has evaluated the dynamic change of ARDS phenotypes [12].

Therefore, we designed this study based on a multi-database to identify longitudinal phenotypes of ARDS. We hypothesized that using diverse data with a longitudinal approach could identify novel longitudinal phenotypes, with different clinical characteristics, mortality rates, and most importantly, responding differently to treatments. We also aimed to explore the dynamic change of ARDS phenotypes across days. Finally, we aimed to derive and validate simplified probabilistic models for phenotype assignment.

## Method

### Study design and participants

We conducted a multi-database study based on the Chinese Database in Intensive Care (CDIC) and RCTs from the National Heart, Lung, and Blood Institute (NHLBI) ARDS Network. The CDIC collected data from 11,560 patients admitted to the Department of Critical Care Medicine, Zhongda Hospital, Southeast University, China, from January 2014 to March 2021, and was classified as a derivation cohort in the present study. Patients in CDIC ﻿who fulfilled the berlin definition of ARDS (detail in Additional file 1: Additional methods) [1] and received mechanical ventilation for at least 24 h ﻿were eligible for inclusion, ﻿we excluded patients younger than 18 years. We only included the first Intensive Care Unit (ICU) admission of each patient. ARDSNet trials including ALVEOLI [13], FACTT [14], EDEN [15] and SAILS [16] were classified as validation cohorts. Involved patients in RCTs were all intubated and received mechanical ventilation. The details of CDIC and ARDSNet trials were presented in Additional file 1: Table S1.

The present study was approved by the Research Ethics Commission of Zhongda Hospital Southeast University (2022ZDSYLL082-P01). For ARDSNet trials, all data were approved by Biologic Specimen and Data Repository Information Coordinating Center (BioLINCC, https://biolincc.nhlbi.nih.gov). STROBE recommendations were followed.

### Data collection and outcomes

The detail of data collection was presented in Additional file 1: Additional methods. The primary outcome in the CDIC derivation cohort was 28-day mortality. The primary outcome was 60-day mortality in ALVEOLI, FACTT and SAILS trials, and was ventilator-free days (VFDs) to 28 days in the EDEN trial. ﻿Other outcomes included the use of neuromuscular blocking agents (NMBAs), ICU mortality, hospital mortality and 90-day mortality were also investigated.

### Quantitative CT analysis

For each patient in CDIC, the whole lung CTs were collected within three days before the initiation of mechanical ventilation, and were treated as lung CTs on Day 0. The cross-sectional lung images were processed and analyzed by a custom-designed software package [17]. After excluding hilar structures, the total lung parenchyma was divided into four regions with different inflated status based on CT attenuation, as follows: non-aerated (voxel density + 100 to − 100 Hounsfield Units, HU), poorly-aerated (− 101 to − 500 HU), well-aerated (− 501 to − 900 HU), and over-inflated lung tissue (− 901 to − 1000 HU) [18]. Fours masks in the total lung were gained as the output, the volume and volumetric percentage of each mask were calculated. The image analysis process was displayed in Additional file 1: Fig. S1. All analyses were performed in Python version 3.7.

### Phenotype derivation

In the CDIC derivation cohort, phenotypes were first studied longitudinally using time-dependent analysis with 24-hourly data from the first four days of invasive ventilation, which were identified by longitudinal LCA [19]. Clinical variables were selected based on their association with the severity or outcome of ARDS, and were used as inputs for the identification of latent classes (Additional file 1: Table S2), including age, minute ventilation, PEEP, driving pressure, mechanical power, ventilatory ratio, PaO_2_/FiO_2_ ratio, heart rate, MAP, pH, creatinine, bicarbonate, lactate and fluid balance. We recorded the most abnormal value if a variable was recorded more than once.

Before longitudinal LCA, we first assessed the distributions and missingness (Additional file 1: Table S3) of candidate variables. Multiple imputations with chained equations (MICE) were used to account for missing data (detail in Additional file 1: Additional methods). Standardized transformation was used for the dataset, and non–normally distributed variables were log-transformed prior to standardized transformation. Longitudinal LCA was fit to the combined datasets of candidate variables from all patients across Days 0, 1, 2 and 3, while allowing phenotype transition across ICU days. We estimated models ranging from two to six classes, and the optimal number of latent classes were selected using the lowest ﻿AIC, SABIC and highest values of entropy. ﻿The minimum number of patients should be over 5% of the entire study population. The ﻿probability of class membership was used to evaluate the robustness of class membership, and the minimum probability should be over 80%. Sensitivity analysis included only the patients who remained on mechanical ventilation for more than 96 h. Longitudinal phenotypes were also validated in ARDSNet trials. LCA was performed using the tidyLPA package in R, ﻿and codes are available in Additional file 2.

We then employed group-based trajectory modeling (GBTM) [20] to assess if the trajectory of a single variable could be used to identify trajectory phenotypes with similar dynamics to those identified by longitudinal LCA. Since longitudinal phenotypes differed most on mechanical power and ventilatory ratio in present study, we applied GBTM on 24-hourly data from the first four days of invasive ventilation to identify trajectories for mechanical power and ventilatory ratio in CDIC (detail in Additional file 1: Additional methods). GBTM was performed using the traj package in Stata.

### Statistical analyses

Values are presented as proportions for categorical variables and means (standard deviations) or medians [interquartile ranges (IQRs)] for continuous variables. For comparisons, we used analysis of variance and the Kruskal–Wallis test for continuous data and the X^2^ test for categorical data.

After the derivation of phenotypes of ARDS, key variables between phenotypes were compared and ﻿visualized with rank plots, ﻿class membership transition over days 0, 1, 2, and 3 were visualized with ﻿alluvial plots. We then assessed the ﻿correlation of the longitudinal phenotypes with pre-selected respiratory variables in CDIC, which included mechanical power, ﻿ventilatory ratio and driving pressure. We employed the Cox proportional hazards model to estimate the effect of a time-varying parameter on a time-to-event outcome in longitudinal phenotypes [21]. Based on the prior knowledge, baseline variables were selected into the Cox model and included pH, PaO_2_/FiO_2_ ratio, PaCO_2_ and dynamic compliance. Since patients with different phenotypes might have different inflated status of lung parenchyma, we also compared the volumetric percentage of each mask across phenotypes on Day 0 in CDIC.

We then compared 28-day mortality or 60-day mortality of patients in different phenotypes (Day 0) in CDIC cohort and ARDSNet trials using Kaplan–Meier curves and log-rank tests; we also performed a multivariate Cox regression model to explore the association and adjusted for age, gender and BMI. ﻿In each of the four ARDSNet trials, ﻿heterogeneity of treatment effect (HTE) was also evaluated by the interaction test to determine if treatment effects were differential across phenotypes (Day 0) in existing trials. ﻿HTE was assessed by the interaction term (Class × Treatment strategy) of the Cox regression for mortality and Poisson regression for VFDs.

Finally, we attempted to construct two parsimonious models to predict phenotypes on Day 0 and Day 2 using baseline variables, respectively. Machine learning algorithms included extreme gradient boosting (XGBoost) and gradient boosted model (GBM) were used to identify the most critical classifier variables. To select the most important variables, variable importance was used for the XGBoost, relative influence factor of the variable was used for GBM (detail in Additional file 1: Additional methods). The common variables in the top five variables of the two machine learning models were selected, and therefore were used to develop final multinomial logistical regression models to identify phenotypes on Day 0 and Day 2, respectively. ﻿The ability of the final model to predict the phenotypes was determined by ﻿calculating the area under the receiver operating characteristic curves (AUROC) for the phenotypes, both in the CDIC and ARDSNet trials.

The p-value was calculated to evaluate the differences between phenotypes, and *P* < 0.05 was considered statistically significant. ﻿The level of significance for the test of interaction was adjusted to 0.0167 according to Bonferroni correction. All statistical analyses were performed using R (version 4.0.3), Stata (16.0) and Python (3.7).

## Results

### Patients in study

In the CDIC derivation cohort, a total of 605 patients met inclusion and exclusion criteria and were enrolled in the final analyses (Additional file 1: Fig. S2). The mean sofa score was 9 (IQR: 6–12) and the PaO_2_/FiO_2_ ratio was 159 mmHg (IQR: 117–210). Pneumonia was the leading cause of ARDS (66.1%). The 28-day all-cause mortality was 37.7%. In the four ARDSNet trials, a total of 3294 patients were included as validation cohorts (549 in the ALVEOLI trial, 1000 in the FACTT trial, 1000 in the EDEN trial and 745 in the SAILS trial), the 60-day mortality across the four trials ranged from 22.7 to 26.9%.

### Derivation of phenotypes for ARDS in CDIC

**Fig. 1:**
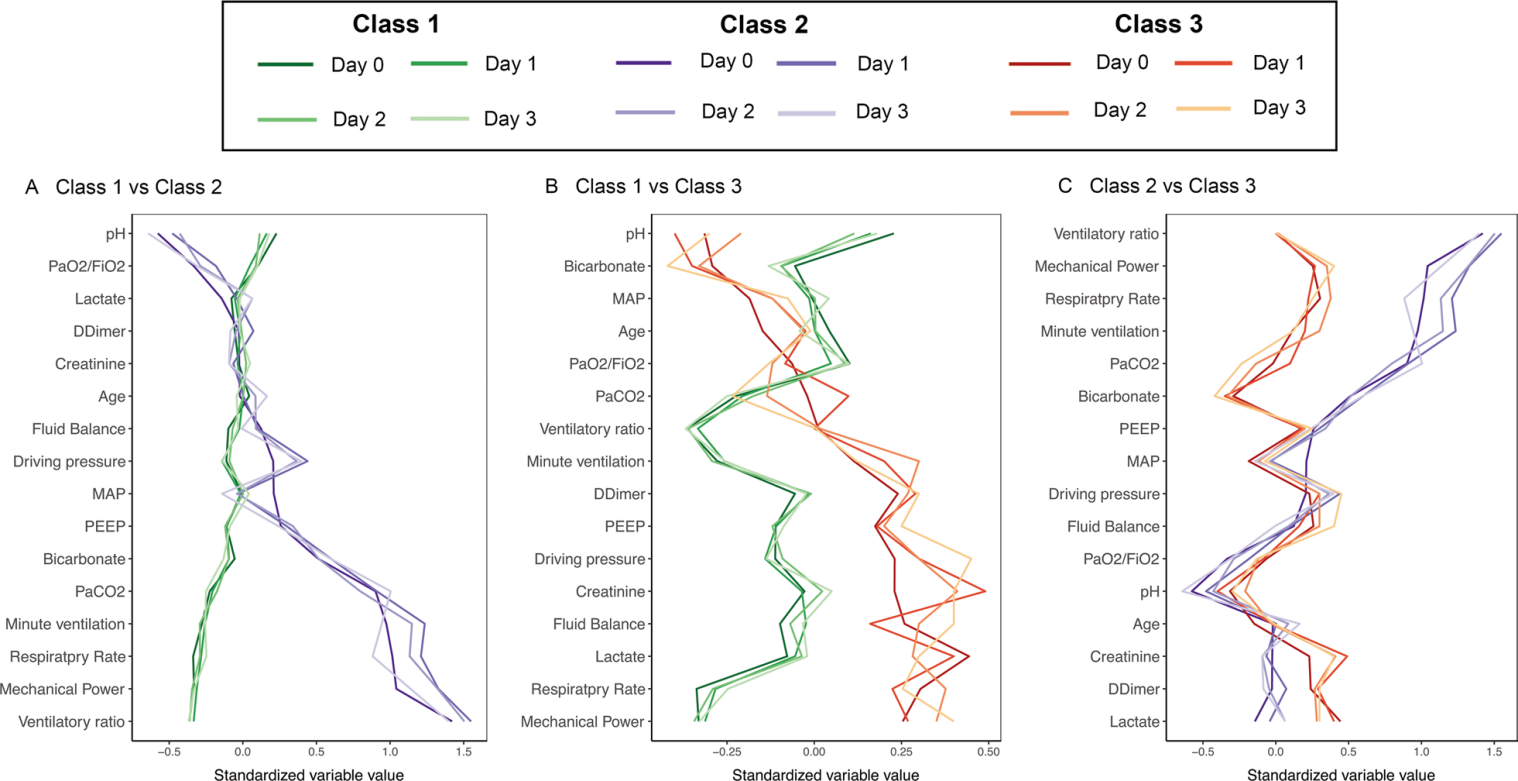
Standardized mean differences between three longitudinal phenotypes in CDIC cohort. **A** Class 1 vs. Class 2. **B** Class 1 vs. Class 3, **C** Class 2 vs Class 3. Fourteen variables were used of phenotyping, but seventeen variables are displayed to give a ﻿comprehensive clinical characteristic of the phenotypes. MAP = mean arterial blood pressure; PaCO_2_ = partial pressure of Carbon Dioxide; PaO_2_ = partial pressure of oxygen; FiO_2_ = fraction of inspired oxygen; PEEP = positive end-expiratory pressure

Using the longitudinal data from the first four days of mechanical ventilation, a three-class model was identified and had the optimal fit in the CDIC derivation cohort (Additional file 1: Table S4). Entropy was 90.6% and the probability of class membership ranged from 87.4 to 97.6%. The longitudinal phenotypes differed broadly in clinical characteristics and organ dysfunction patterns (Table 1 and Additional file 1: Table S5). The standardized mean differences of main clinical characteristics between phenotypes over time were shown in Fig. 1. Class 1 (*n* = 400, 66.1% of the cohort) was the largest phenotype, and had fewer abnormal values and less organ dysfunction. Class 2 (*n* = 102, 16.9% of the cohort) was characterized by the highest minute ventilation, driving pressure, mechanical power, ventilatory ratio and the lowest PaO_2_/FiO_2_ during the first four days of mechanical ventilation, which can be called pulmonary mechanical dysfunction phenotype. Class 3 (*n* = 103, 17% of the cohort) was characterized by the highest creatinine, lactate and the lowest bicarbonate, MAP, and a higher proportion of patients received vasopressors compared to other phenotypes, which can be called extra-pulmonary dysfunction phenotype (Additional file 1: Figs. S3–S5). Interleukin-6 (IL-6) was highest in Class 3 compared to other phenotypes (Additional file 1: Fig. S6). As for the causes of ARDS, Class 3 had the lowest proportion of pneumonia and the highest proportion of sepsis compared to other phenotypes.

**Table 1:** Clinical characteristics and outcomes of the longitudinal phenotypes on Day 0 in the CDIC

	All (*n* = 605)	Longitudinal phenotypes of ARDS			*P* value
		Class 1 (*n* = 400)	Class 2 (*n* = 102)	Class 3 (*n* = 103)	
Age (years)	65 (53–76)	66 (54–77)	65 (54–73)	63 (52–75)	0.18
Male (gender), *n* (%)	423 (69.9)	283 (70.8)	67 (65.7)	73 (70.9)	0.59
BMI (kg/m^2^)	23.5 (20.9–26.0)	23.7 (21.5–26.0)	23.4 (20.8–25.7)	22.9 (20.8–25.8)	0.35
ARDS Primary risk factor, n (%)					0.015
Pneumonia	400 (66.1)	276 (69.0)	71 (69.6)	53 (51.5)	
Sepsis	97 (16.0)	59 (14.8)	10 (9.8)	28 (27.2)	
Aspiration	49 (8.1)	28 (7.0)	11 (10.8)	10 (9.7)	
Other	59 (9.8)	37 (9.2)	10 (9.8)	12 (11.6)	
SOFA score	9 (6–12)	8 (6–11)	9 (6–13)	11 (9–14)	< 0.001
APACHE II score	22 (17–28)	21 (16–27)	24 (17–31)	26 (22–32)	< 0.001
Severity of ARDS at baseline, n (%)					< 0.001
Mild	175 (28.9)	133 (33.2)	15 (14.7)	27 (26.2)	
Moderate	321 (53.1)	208 (52.0)	57 (55.9)	56 (54.4)	
Severe	109 (18.0)	59 (14.8)	30 (29.4)	20 (19.4)	
Parameters of mechanical ventilation in the first 24 h					
Respiratory rate (breaths min^−1^)	25 (22–30)	24 (21–27)	32 (29–36)	28 (25–31)	< 0.001
Tidal volume (ml/kg PBW)	8.3 (7.1–9.7)	8.1 (7.0–9.4)	9.2 (7.5–10.5)	8.6 (7.3–10.0)	< 0.001
Minute ventilation (L/min)	12.8 (10.5–15.8)	11.8 (9.7–14.0)	16.9 (14.0–20.2)	13.7 (11.4–16.2)	< 0.001
PEEP (cmH_2_0)	9 (7–11)	9 (7–10)	10 (8–12)	10 (8–11)	< 0.001
Peak Pressure (cmH_2_0)	24 (21–27)	23 (21–26)	25 (22–29)	24 (22–29)	< 0.001
Driving pressure (cmH_2_0)	15 (12–19)	15 (12–18)	16 (13–20)	15 (13–19)	0.019
Mechanical power (J/min)	20.4 (15.8–26.1)	18.9 (14.4–22.4)	30.3 (25.4–35.5)	21.9 (18.4–28.1)	< 0.001
Compliance (ml/cmH_2_0)	33.8 (26.3–43.8)	33.9 (26.9–43.2)	33.9 (25.0–47.0)	33.5 (25.4–42.9)	0.87
Ventilatory ratio	1.90 (1.43–2.47)	1.69 (1.31–2.11)	3.26 (2.73–3.71)	1.97 (1.53–2.50)	< 0.001
PaCO_2_ (mmHg)	33.9 (28.5–40.0)	32.3 (28.1–37.9)	40.4 (35.5–53.4)	33.8 (27.5–41.1)	< 0.001
PaO_2_/FiO_2_ ratio (mmHg)	159 (117–210)	162 (124–220)	142 (90–186)	160 (113–202)	0.0012
Vasopressor use in the first 24 h, *n* (%)	481 (79.5)	301 (75.3)	87 (85.2)	93 (90.3)	< 0.001
Vital signs in the first 24 h					
Heart rate (beats min^−1^)	98 (94–99)	97 (92–98)	98 (95–99)	110 (101–120)	< 0.001
MAP (mmHg)	69 (65–75)	69 (65–75)	69 (65–80)	66 (62–71)	0.0092
Temperature (℃)	37.8 (37.0–38.4)	37.5 (37.0–38.2)	37.9 (37–38.5)	38.2 (37.7–38.8)	< 0.001
Laboratory data in the first 24 h					
pH	7.39 (7.33–7.43)	7.41 (7.36–7.45)	7.35 (7.25–7.40)	7.35 (7.29–7.41)	< 0.001
BUN (mg/dl)	9.6 (6.4–15.2)	9.2 (6.1–14.0)	10.5 (6.6–17.2)	11.7 (7.5–16.4)	0.013
Creatinine (mmol/L)	100 (69–165)	92 (66.5–147)	104.5 (70–170.8)	134 (84–193.5)	< 0.001
Bicarbonate (mmol/L)	20.7 (17.8–23.7)	20.6 (17.8–23.3)	23.0 (19.8–26.2)	19.8 (16.3–21.9)	< 0.001
Lactate (mmol/L)	1.9 (1.2–3.0)	1.9 (1.2–2.8)	1.7 (1.2–2.5)	2.6 (1.8–4.3)	< 0.001
Fluid balance in the first 24 h (L)	1.38 (-0.35–4.21)	1.05 (-0.56–3.78)	2.41 (-0.35–4.48)	2.89 (0.54–4.81)	< 0.001
Clinical outcomes					
Alive and VFDs at Day 28 (days)	5.9 (0–21.4)	13.1 (0–22.4)	0 (0–15.5)	0 (0–13.9)	< 0.001
ICU mortality, *n* (%)	176 (29.1)	92 (23.0)	35 (34.3)	49 (47.6)	< 0.001
Hospital mortality, *n* (%)	189 (31.2)	102 (25.5)	36 (35.3)	51 (49.6)	< 0.001
28-day mortality, *n* (%)	228 (37.7)	122 (30.5)	48 (47.1)	58 (56.3)	< 0.001

*BMI* body mass index, *ARDS* acute respiratory distress syndrome, *PBW* predicted body weight, *SOFA* sequential organ failure assessment, *APACHE* acute physiology and chronic health evaluation II, *PEEP* positive end-expiratory pressure, *PaCO*_2_ partial pressure of Carbon Dioxide, *PaO*_2_ partial pressure of oxygen, *MAP* mean arterial blood pressure, *BUN* blood urea nitrogen, *VFD* ventilator-free days, *ICU* intensive care unit

**Fig. 2:**
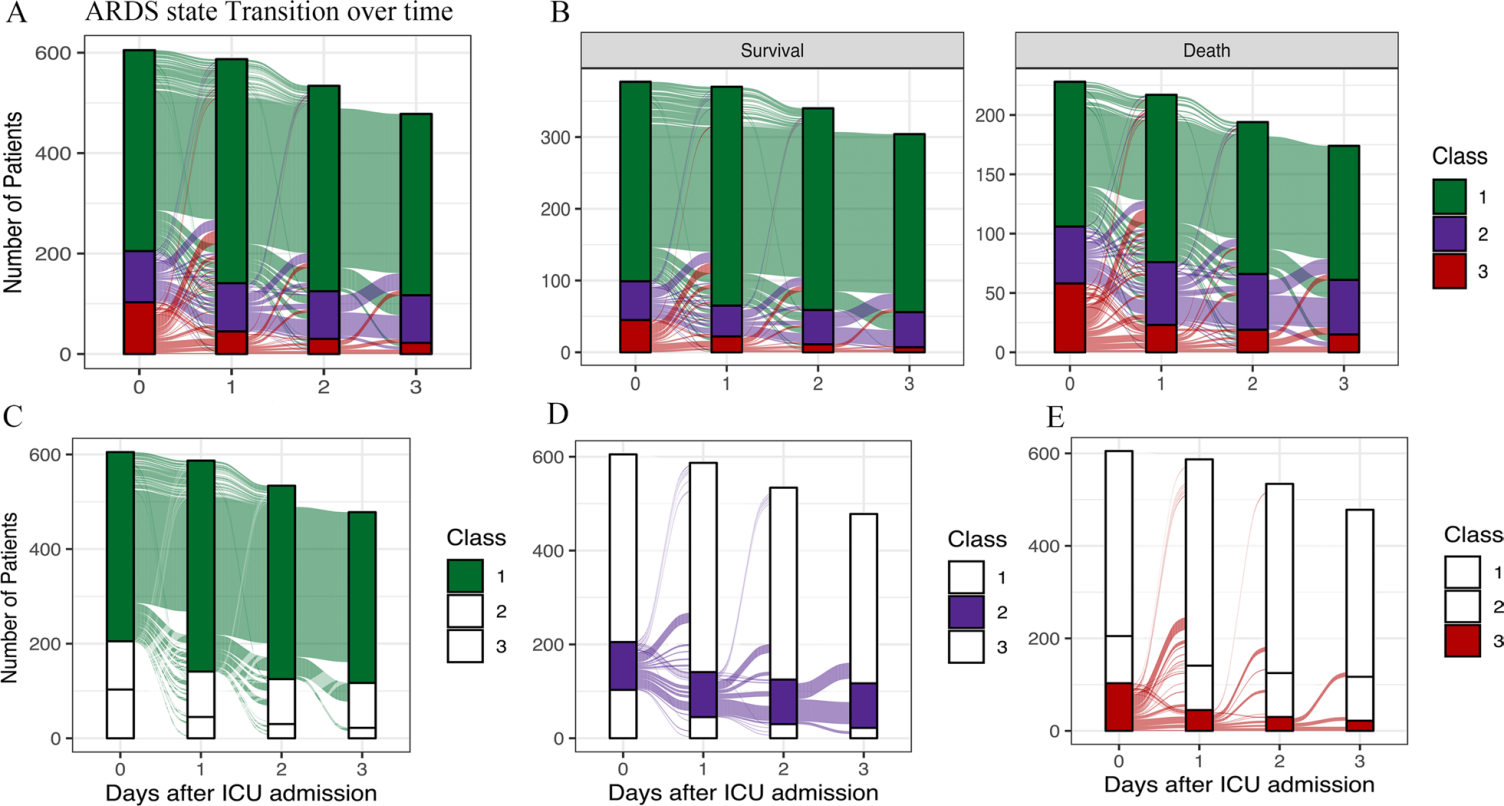
ARDS state transition over days 0, 1, 2 and 3 in CDIC cohort. **A** ARDS state transition over days in whole patients. **B** ARDS state transition over days based on the survival state on Day 28. **C** ARDS state transition over days for patients in Class 1; **D** ARDS state transition over days for patients in Class 2; **E** ARDS state transition over days for patients in Class 3. ARDS = acute respiratory distress syndrome; ICU = Intensive care unit

Most patients (56.9%) changed their phenotypes at least once during the first four days of mechanical ventilation (Fig. 2). Class 1 had the largest number over all study days, while the number of Class 3 gradually decreased. Sensitivity analysis including only the patients who remained on mechanical ventilation for more than 96 h (*n* = 459, 75.8% of the cohort) and yielded a similar result, with only 4.4% of patients on Day 0, 3.3% on Day 1, 4.3% on Day 2 and 4.8% on Day 3 changing class membership (Additional file 1: Fig. S7).

After GBTM, three distinct trajectories of mechanical power and ventilatory ratio were observed and had the optimal fit, as follows: a sustained low value, or a sustained moderate value, or a sustained high value for mechanical power or ventilatory ratio ﻿over the first four days of invasive ventilation (Additional file 1: Fig. S8). While the trajectories and longitudinal phenotypes did not overlap much in mechanical power and ventilatory ratio (Additional file 1: Fig. S9). The clinical characteristics and outcomes between trajectories were shown in Additional file 1: Table S6.

### Interaction between phenotypes and respiratory parameters on mortality

**Fig. 3:**
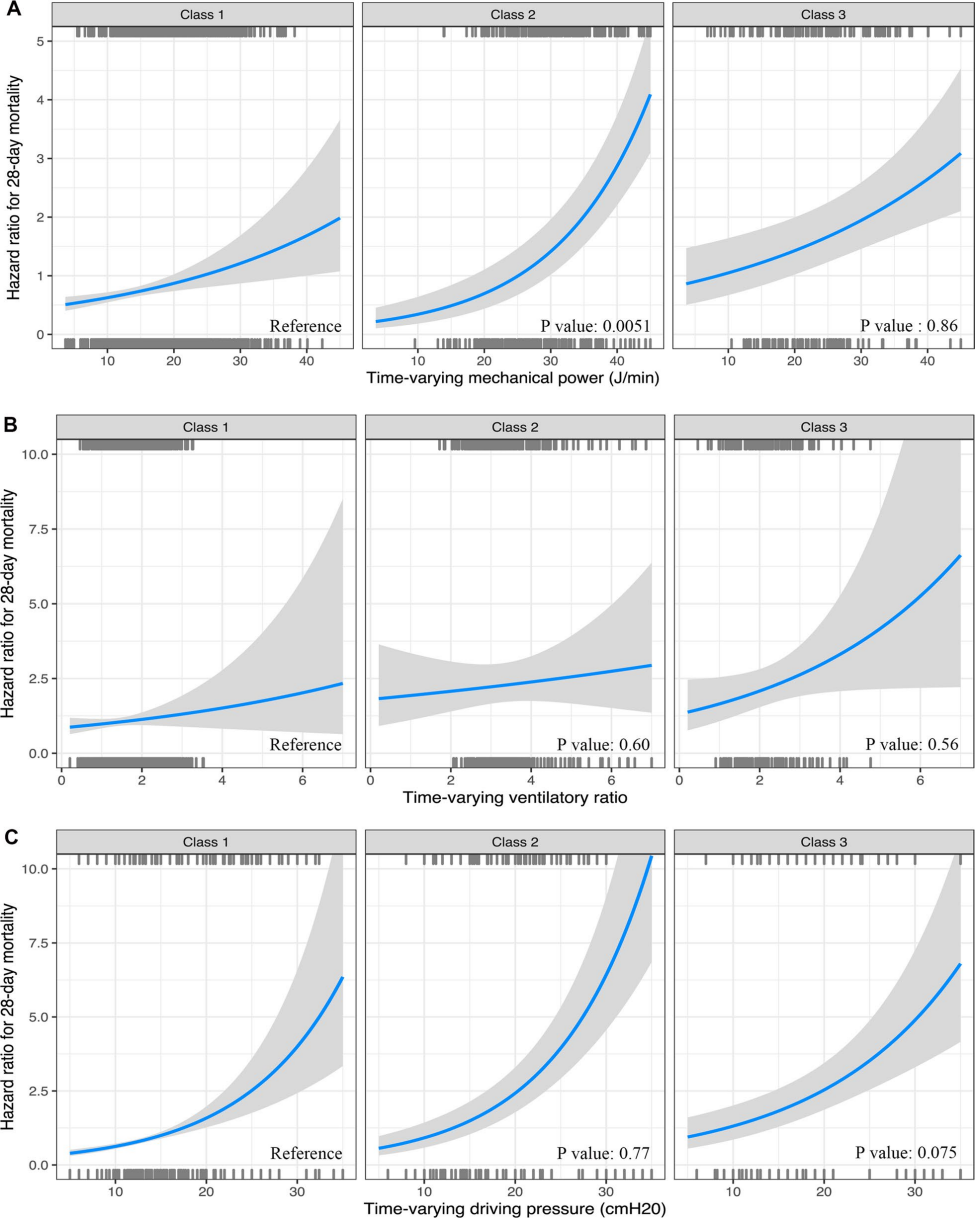
Interaction between longitudinal phenotypes of ARDS with time-varying mechanical power (**A**), ventilatory ratio (**B**) and driving pressure (**C**) on 28-day mortality in CDIC cohort. *P* values represent p values for interaction

In the Cox proportional hazards model, after adjusting for pH, PaCO_2_, PaO_2_/FiO_2_ ratio and respiratory system compliance, there was a significant interaction between mechanical power and phenotypes (Fig. 3 and Additional file 1: Tables S7–S9). While time-varying mechanical power was more significantly associated with 28-day mortality in Class 2 patients compared to other phenotypes in the CDIC cohort (HR 1.04, 95% CI 1.01–1.07; p for interaction = 0.0051). No significant interaction was detected between time-varying ventilatory ratio or driving pressure and phenotypes.

### Correlation of phenotypes with lung CT features in CDIC

A total of 427 patients performed lung CT within three days before the initiation of mechanical ventilation: 328 (82%) patients in Class 1, 72 (70.6%) patients in Class 2 and 27 (26.2%) patients in Class 3. Compared to other phenotypes, patients in Class 2 had the lowest ﻿proportion of ﻿normally aerated lung volume, and the highest proportion of ﻿poorly-aerated lung volume on Day 0 (Additional file 1: Table S10).

### Validation of longitudinal phenotypes for ARDS in ARDSNet trials

Longitudinal phenotypes were also validated in the four ARDSNet trials, and showed the same optimal phenotype numbers as observed in the derivation cohort (Additional file 1: Fig. S10). The phenotype sizes varied across the trials: Class 1 ranged from 41.8 to 62.0%, Class 2 ranged from 14.4 to 34.9%, and Class 3 ranged from 9.9 to 37.2%. The clinical characteristics of the phenotypes were largely ﻿similar to those of the derivation cohort (Additional file 1: Tables S11–S14 and Additional file 1: Figs. S11–S14). Specifically, Class 1 was characterized by less organ dysfunction, Class 2 was predominantly characterized by pulmonary mechanical dysfunction phenotype and Class 3 was mainly characterized by extra-pulmonary organ dysfunction. IL-6 and Soluble intercellular adhesion molecule-1 (sICAM-1) were assessed in the ALVEOLI trial, compared to Class 1 and Class 2, both IL-6 and sICAM-1 were highest in Class 3 (Additional file 1: Fig. S6).

### Relationship between phenotypes and clinical outcomes

**Fig. 4:**
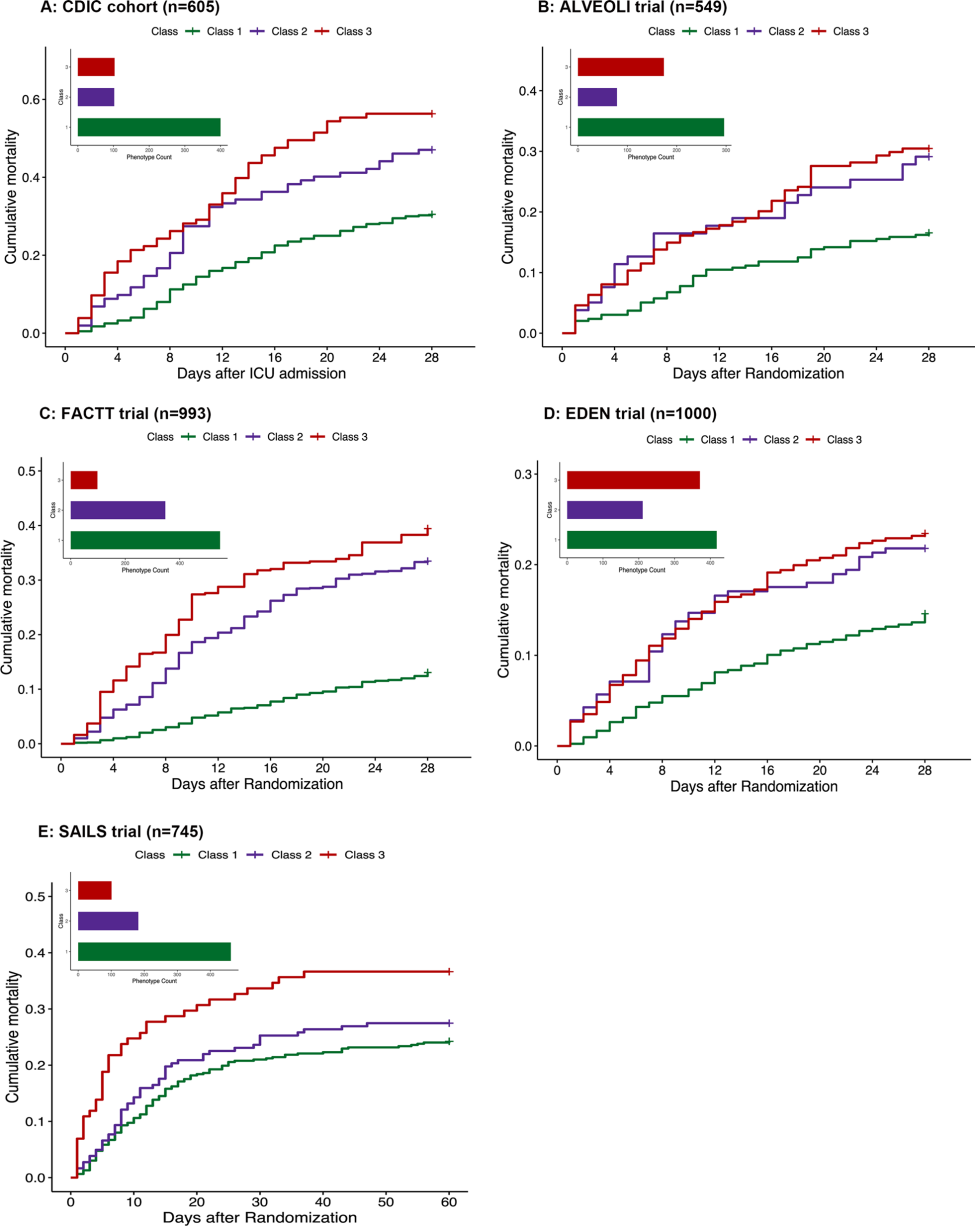
Mortality by longitudinal phenotypes on Day 0. **A** 28-day mortality stratified by phenotype assignment on Day 0 in CDIC. After adjusting for age, gender and BMI, compared with Class 1, the adjusted hazard ratio of 28-day mortality for Class 2 and Class 3 were 1.86 (95% CI 1.33–2.62), 2.48 (95% CI 1.81–3.41), respectively. **B**–**E** 60-day mortality stratified by phenotype assignment on Day 0 in ARDSNet Trials. In ALVEOLI trial, compared with Class 1, the adjusted hazard ratio of 60-day mortality for Class 2 and Class 3 were 2.58 (95% CI 1.61–5.12), 2.56 (95% CI 1.76–3.72), respectively. In FACTT trial, compared with Class 1, the adjusted hazard ratio of 60-day mortality for Class 2 and Class 3 were 2.72 (95% CI 2.05–3.61), 3.02 (95% CI 2.07–4.40), respectively. In EDEN trial, compared with Class 1, the adjusted hazard ratio of 60-day mortality for Class 2 and Class 3 were 1.83 (95% CI 1.26–2.64), 1.53 (95% CI 1.13–2.07), respectively. In SAILS trial, compared with Class 1, the adjusted hazard ratio of 60-day mortality for Class 2 and Class 3 were 1.29 (95% CI 0.92–1.81), 2.09 (95% CI 1.43–3.04), respectively

In the CDIC derivation cohort, the 28-day mortality rates were highest in Class 3 (56.3%), followed by Class 2 and Class 1 (47.1% and 30.5%, respectively). Kaplan–Meier survival curves showed the 28-day mortality was highest in Class 3 (*P* < 0.0001) compared with other phenotypes. Across all ARDSNet trials, the highest 60-day mortality occurred in Class 3 compared with other phenotypes (*P* < 0.001). In the ALVEOLI trial, 60-day mortality was 18.9% for Class 1, 32.9% for Class 2 and 35.6% for Class 3. In the FACTT trial, 60-day mortality was 17.7% for Class 1, 35.4% for Class 2 and 47.9% for Class 3. In the EDEN trial, the 60-day mortality was 18.2% for Class 1, 24.2% for Class 2 and 27% for Class 3. In the SAILS trial, the 60-day mortality was 24.2% for Class 1, 27.5% for Class 2 and 36.6% for Class 3 (Fig. 4). The derived phenotypes demonstrated significant differences in VFDs across the cohort and trials. Specifically, patients assigned to Class 1 had the most VFDs and patients assigned to Class 3 had the least VFDs (In the EDEN trial, patients in Class 2 had the least VFDs).

### Heterogeneity of treatment effect within phenotypes

We assessed HTE in four ARDSNet trials based on the phenotypes on Day 0. In the ALVEOLI trial, a significant interaction between phenotypes and PEEP strategy on 60-day mortality was detected, patients classified to Class 2 had a 60-day mortality of 23.6% when received lower PEEP strategy, versus 54.2% when received higher PEEP strategy. In contrast, patients classified to Class 3 had a 60-day mortality of 41.6% when received lower PEEP strategy, versus 30.9% when received higher PEEP strategy (P for interaction = 0.0016) (Additional file 1: Fig. S15 and Table 2). In the FACTT trial, we also identified a significant effect of the interaction between phenotypes and fluid management strategy on 60-day mortality. Specifically, mortality among Class 2 patients was 30.8% with the fluid conservative strategy compared to 39.3% with the fluid liberal strategy. While mortality among Class 3 patients was 58.5% with the fluid conservative strategy compared to 35.6% with the fluid liberal strategy (P for interaction = 0.0068) (Additional file 1: Fig. S16 and Table 3). No significant HTE was observed in the EDEN and SAILS trials (Additional file 1: Figs. S17–S18).

**Table 2:** Heterogeneity of treatment effect to PEEP strategy within phenotypes in ALVEOLI Trial

	Class 1		Class 2		Class 3	
	Lower PEEP (*n* = 141)	Higher PEEP (*n* = 151)	Lower PEEP (*n* = 55)	Higher PEEP (*n* = 24)	Lower PEEP (*n* = 77)	Higher PEEP (*n* = 97)
60-day mortality, *n* (%)	23 (16.3)	33 (21.3)	13 (23.6)	13 (54.2)	32 (41.6)	30 (30.9)

	Interaction term		HR (95% CI)		*P* value for interaction	
Interaction between phenotypes (D0) and PEEP strategy						
60-day mortality, *n* (%)	Class 1: Class 2		2.16 (0.85–5.50)		0.11	
	Class 1: Class 3		0.49 (0.23–1.02))		0.056	
	Class 2: Class 3		0.23 (0.09–0.57)		0.0016	

*PEEP* positive end-expiratory pressure, *HR* hazard ratio, *CI* Confidence interval

**Table 3:** Heterogeneity of treatment effect to Fluid management strategy within phenotypes in FACTT Trial

	Class 1		Class 2		Class 3	
	Conservative (*n* = 290)	Liberal (*n* = 258)	Conservative (*n* = 156)	Liberal (*n* = 191)	Conservative (*n* = 53)	Liberal (*n* = 45)
60-day mortality, *n* (%)	47 (16.2)	50 (19.4)	48 (30.8)	74 (39.3)	31 (58.5)	16 (35.6)

	Interaction term		HR (95% CI)		*P* value for interaction	
Interaction between phenotypes (D0) and Fluid management strategy						
60-day mortality, *n* (%)	Class 1: Class 2		0.90 (0.53–1.54)		0.71	
	Class 1: Class 3		2.39 (1.16–4.92)		0.018	
	Class 2: Class 3		2.64 (1.30–5.35))		0.0068	

*HR* hazard ratio, *CI* Confidence Interval

### Parsimonious probabilistic models to identify phenotypes

**Fig. 5:**
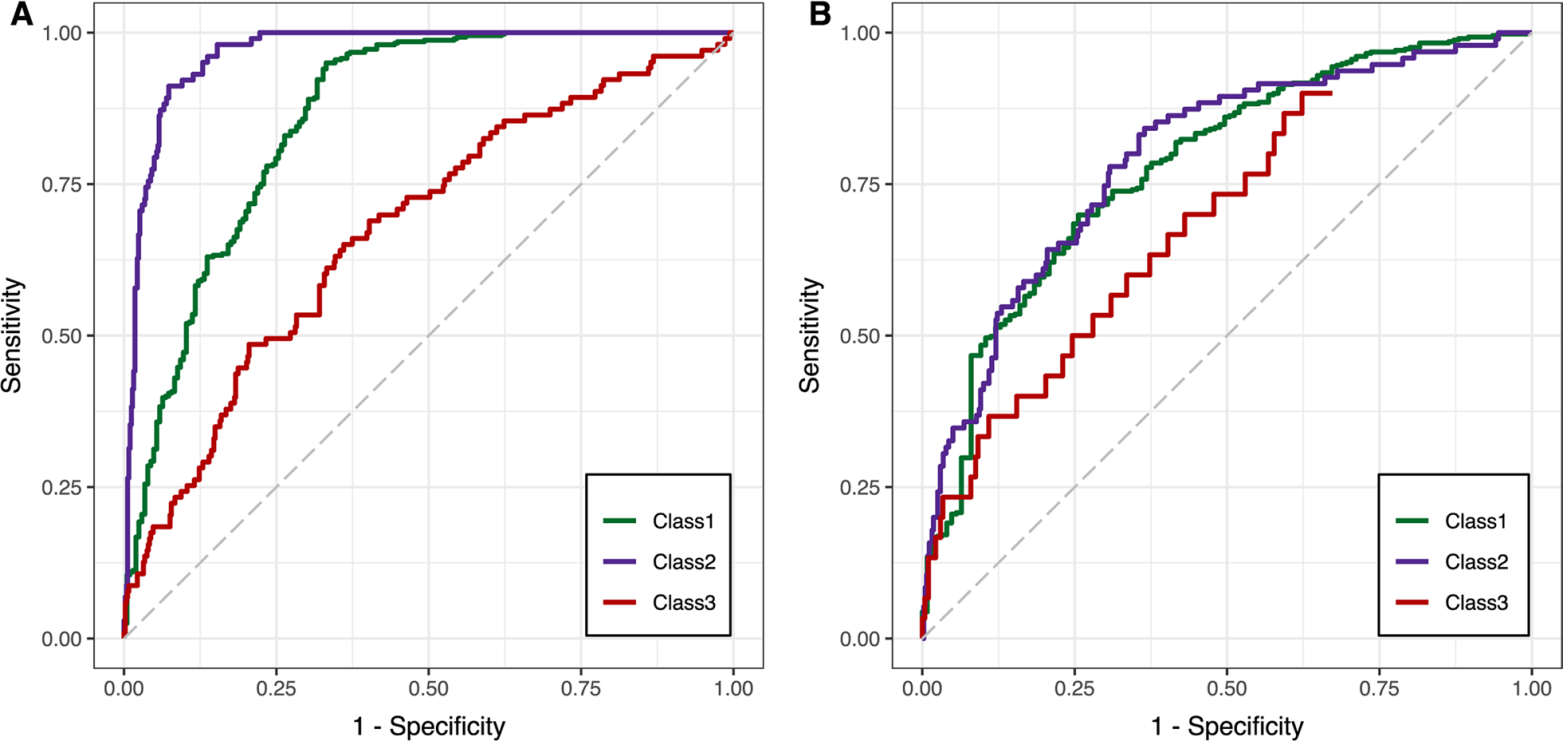
Receiver operating curves for the multinomial regression models for predicting phenotypes on Day 0 (**A**) and Day 2 (**B**) in CDIC cohort

The most important classifier variables for predicting phenotypes were shown in Additional file 1: Fig. S19–S20. Ultimately, model A included mechanical power, ventilatory ratio, respiratory rate and pH was constructed to predict phenotypes on Day 0, and model B included ventilatory ratio, mechanical power, and creatinine was constructed to predict phenotypes on Day 2 (Additional file 1: Table S15). The AUROC for model A to predict phenotypes on Day 0 was 0.86 (95% CI 0.82–0.90) for Class 1, 0.97 (95% CI 0.95–0.98) for Class 2, and 0.67 (95% CI 0.62–0.73) for Class 3. While for the prediction of phenotypes on Day 2, the AUROC of model B was 0.78 (95% CI 0.72–0.83) for Class 1, 0.80 (95% CI 0.74–0.86) for Class 2, and 0.70 (95% CI 0.60–0.79) for Class 3 (Fig. 5). For the external validation cohorts, the predictive ability of the parsimonious models was shown in Additional file 1: Table S16.

## Discussion

The novel findings of our analyses can be summarized as follows. First, based on a multi-database, we derived and validated three novel longitudinal phenotypes of ARDS, with different severity of pulmonary mechanics, organ dysfunction, chest CT features, and outcomes. Second, we detected a significant HTE between phenotypes and treatment strategies in the ALVEOLI and FACTT trials. Third, we developed two simplified probabilistic models to predict ARDS phenotypes, potentially applicable to other cohorts.

Longitudinal phenotypes in present study differed from the prior ARDS phenotypes in several critical aspects. First, different variables were applied to identify the phenotypes. Calfee and colleagues focused mainly on the degree of inflammatory conditions of ARDS, and first derived two inflammatory phenotypes using numerous inflammatory factors in the ALVEOLI trial [7]. The two inflammatory phenotypes were subsequently identified in other ARDSNet trials and cohorts [22, 23]. Recently, two studies declared that inflammatory phenotypes could also be accurately predicted without biomarker data using supervised learning approaches [24, 25]. Another study identified three phenotypes of ARDS based on routine medical data in the eICU ﻿database [26]. From another perspective, we concentrated primarily on pulmonary mechanics and organ function, including mechanical power and ventilatory ratio, which were both significantly associated with mortality in ARDS [27, 28], and had never been employed to identify phenotypes in traditional ARDS. Second, the previous ARDS phenotypes described above were largely used cross-sectional data, unlike in our study using longitudinal data. While longitudinal data might be more informative in identifying phenotypes. In a prospective cohort study including COVID-19 related ARDS patients, ﻿ Bos LDJ et al. [29] did not yield any latent classes using cross-sectional data, but found that a two-class model best fit the cohort using the longitudinal data.

Our phenotypes were partially consistent with the two longitudinal respiratory subphenotypes in COVID-19 patients [29]. Specifically, subphenotype 1 was characterized by the least abnormalities of pulmonary mechanics, while subphenotype 2 was characterized by increasing minute ventilation, mechanical power and ventilatory ratio. The characteristics of Class 1 and Class 2 in our study were similar to subphenotype 1 and subphenotype 2, respectively. Additionally, we discovered a novel longitudinal phenotype (Class 3) which was predominantly characterized by extra-pulmonary dysfunction, since we enrolled patients with other causes-related ARDS and the model included more markers of organ dysfunction.

Phenotypes of disease could change dynamically accompanied by treatment response and disease progression. Limited studies have addressed the dynamic change of ARDS phenotypes. ﻿Delucchi and colleagues [12] performed a secondary analysis of the ARMA and ALVEOLI trials to determine the stability of ARDS subphenotypes over time. LCA was conducted separately at day 0 and day 3 using inflammatory factors, and a two-class model was identified in both day 0 and day 3. Latent transition analysis demonstrated that most patients (> 94%) stayed in the same class from day 0 to day 3 in both trials. However, whether the levels of inflammatory factors in Class 2 on day 3 were similar to Class 2 on day 0 was unclear, since they performed LCA independently on days 0 and 3. That said, Class 2 on day 3 might differ from Class 2 on day 0. In comparison, we found that 56.9% of the patients changed their phenotypes at least once during the study days. Except for the different variables included in the model, the different approaches might explain the difference in proportions. In present study, LCA was employed using the longitudinal data, which was fit to the combined datasets from all patients across all study days. The values of pulmonary mechanics or organ dysfunction markers were similar in same class across all study days.

The radiographic severity differed in phenotypes in our study. Previous research provided conflicting evidence concerning the association between radiographic severity and disease severity of ARDS patients. A prospective cohort study employed ﻿a radiographic assessment of lung edema (RALE) score to reflect the radiographic severity, and found that the RALE score was neither associated with ARDS severity grouped by PaO_2_/FiO_2_ ratio nor pulmonary mechanics [30]. Whereas a secondary analysis of the FACTT trial showed that a lower baseline RALE score was independently associated with a higher PaO_2_/FiO_2_ ratio [31]. RALE score was calculated based on the chest X-ray, which might limit the diagnostic accuracy of radiographic severity. We employed quantitative CT analysis to assess the radiographic severity directly, and found that patients with pulmonary mechanical dysfunction phenotype had the lowest proportion of normally aerated lung volume and the highest proportion of poorly-aerated lung volume compared to other phenotypes.

Accurate and precise phenotypes will more effectively guide individualized treatment strategies. The original FACTT trial found no difference in 60-day mortality between conservative and liberal fluid management [14]. ﻿After that, Famous et al. discovered that the conservative strategy was associated with improved mortality in patients with hyperinflammatory phenotype but had the opposite effect in patients with hypoinflammatory phenotype [9]. Inconsistent with the previous study, we found that fluid management strategies had no effect in Class 1 patients, while Class 2 patients can particularly benefit from conservative fluid strategy and Class 3 patients (similar to hyperinflammatory phenotype) can more strongly benefit from the liberal fluid strategy. ﻿Several explanations exist. Compared to other phenotypes in the FACTT trial, Class 3 was more likely to use vasopressor, had the lowest pH, serum bicarbonate and mean arterial blood pressure. Together these observations strongly implied inadequate effective circulating blood volume in Class 3 patients, whose are required early aggressive fluid resuscitation. A retrospective study included septic patients with ARDS and declared that patients who received adequate early fluid administration followed by later conservative fluid management had the lowest mortality [32]. More research regarding the effect of fluid management strategy on mortality in various phenotypes of ARDS is needed.

We also detected a significant interaction between phenotypes and PEEP strategy on 60-day mortality in the ALVEOLI trial [13]. The effect of PEEP is primarily related to the balance between the number of alveoli that are recruited to participate in ventilation and the amount of lung that is overdistended when PEEP is applied. Unlike inflammatory response-guided PEEP strategy in the previous study [8], we chose PEEP strategy from a physiological perspective. Class 2 in the ALVEOLI trial was characterized by the highest driving pressure, ventilatory ratio and PaCO_2_, which suggested a higher proportion of dead space in Class 2 patients. This can interpret the beneficial effect of lower PEEP in Class 2. No significant interaction between phenotypes and treatment strategies was observed in the EDEN and SAILS trials. Possible reasons have been discussed elsewhere [33].

Our study is the first to identify longitudinal phenotypes for various etiologies ARDS based on comprehensive metrics and multi-database. Unlike previous inflammatory phenotypes, we also detected novel HTE between longitudinal phenotypes and treatment strategies (PEEP and Fluid management strategy). This study also has several limitations. First, although we identified and validated the longitudinal phenotypes in five separate cohorts, several factors might affect the robustness of phenotypes, such as the missing data, the treatments and ventilator settings. In four ARDSNet trials, the phenotypes can be affected by the randomization arms, although we excluded PEEP in the ALVEOLI trial and fluid balance in the FACTT trial when performing LCA, we cannot exclude the impact of the randomization arms since the respiratory variables are both mathematically and physiologically coupled. Meanwhile, such variations can create inconsistencies in the construct of phenotypes between studies. Second, most patients changed their states at least once during the study days, which may limit the translational premise of our approach for therapeutic targeting in future ARDS clinical trials. Third, the inflammatory factors are limited in CDIC and ARDSNet trials. Therefore, our phenotypes cannot compare to the inflammatory phenotypes directly and precisely. Meanwhile, the performance of parsimonious models was poor for predicting Class 3, since Class 3 was characterized as the pro-inflammatory ARDS, and we did not include any inflammatory factors in the model. More studies are required further to investigate the predictive performance of the model after including inflammatory factors. Fourth, we found that the longitudinal phenotypes responded differently to treatment, which was derived from a secondary analysis of ARDSNet trials. Whether the results reflect the true biology was unclear, and the treatment benefits need prospective validation. Additionally, the significant HTE was only against Day 0 phenotypes and may not against longitudinal assignments. Finally, the longitudinal phenotypes are dynamic and patients can switch classes during the first four days of invasive ventilation, although we developed models to predict phenotypes on Day 0 and Day 2, we can neither predict the phenotypes at any time nor the change of the phenotypes.

## Conclusion

In this retrospective analysis of a multi-database from patients with ARDS, three novel longitudinal phenotypes were identified, with various sites and severity of organ dysfunction, and different clinical outcomes. Most patients changed their phenotypes at least once during the first four days of invasive ventilation. Besides, the analysis suggested heterogeneity of treatment effects within phenotypes on Day 0 in the ALVEOLI and FACTT trials.

## Supplementary Information

**Additional file 1**. Supplemental methods, Figures and Tables.

**Additional file 2**. R code for data analysis.

## Data Availability

For data in CDIC cohort, data are available upon reasonable request and with the approval from the Department of Critical Care Medicine, Zhongda Hospital, School of Medicine, Southeast University. The datasets of ARDSNets are available in the BioLINCC website (https://biolincc.nhlbi.nih.gov).

## Abbreviations

ARDS: Acute respiratory distress syndrome
RCTs: Randomized controlled trials
CDIC: Chinese Database in Intensive Care
NHLBI: National Heart, Lung, and Blood Institute
ICU: Intensive Care Unit
VFDs: Ventilator-free days
NMBAs: Neuromuscular blocking agents
LCA: Latent class analysis
PEEP: Positive end-expiratory pressure
MICE: Multiple imputations with chained equations
GBTM: Group-based trajectory modeling
HTE: Heterogeneity of treatment effect
XGBoost: Extreme gradient boosting
GBM: Gradient boosted model
AUROC: Area under the receiver operating characteristic curves
IL-6: Interleukin-6
SICAM-1: Soluble intercellular adhesion molecule-1
