# Supplementary material for: Longitudinal phenotypes in patients with acute respiratory distress syndrome: a multi-database study
Source: Crit Care. 2022 Nov 4;26:340. doi: 10.1186/s13054-022-04211-w (PMC9635207; doi:10.1186/s13054-022-04211-w)
Supplement: Supplementary file 1 — Additional file 1. Supplemental methods, Figures and Tables. [file 13054_2022_4211_MOESM1_ESM.docx]

**Additional methods:**

1. **Approach to identify ARDS patients**

To identify ARDS patients in CDIC cohort, we first extracted all acute respiratory failure patients with mechanical ventilation from the database, then medical history, radiographs (lung CT or chest X-ray), blood gases and PEEP were reviewed by experienced intensivists and radiologist, and patients were diagnosed with ARDS if they met the Berlin definition. If there is a dispute, the diagnosis was determined by a more experienced intensivist.

**B. Data Collection**

Clinical and laboratory variables were collected on Day 0, 1, 2 and 3. In CDIC derivation cohort, Day 0 was defined as the first ﻿calendar day that a patient received invasive ventilation. In ARDSNet trials, Day 0 was defined as the first day of randomization. All demographic data including age, gender, primary lung injury of ARDS, height and weight on admission, chronic comorbidities were collected.

Longitudinal data of preselected variables were also collected. Respiratory variables included respiratory rate, tidal volume, minute ventilation, positive end–expiratory pressure (PEEP), peak pressure (Ppeak) and Plateau pressure (Pplat) were obtained. For patients who received ventilation in a volume–controlled assist mode, driving pressure (ΔP) was calculated as Pplat minus PEEP, ﻿if not specified, Ppeak was considered equal to Pplat in pressure-regulated modes other than pressure support ventilation. Mechanical power (MP) was calculated as [﻿0·098*respiratory rate*tidal volume*(Ppeak–(0·5*ΔP))]. ﻿Ventilatory ratio is defined as [minute ventilation * arterial PCO_2_/ [predicted body weight (PBW) * 100 * 37.5], PBW was calculated as equal to [50 + 0.91 (centimeters of height – 152.4)] in males, and [45.5 + 0.91 (centimeters of height – 152.4)] in females. Hemodynamic variables (heart rate, systolic blood pressure, diastolic blood pressure, use of vasopressor and lactate level (if available)), markers of organ dysfunction and injury (platelets, creatinine, bilirubin and PaO_2_/FiO_2_ ratio), inflammatory markers (Interleukin-6 and high-sensitive C-reactive protein in CDIC, IL-6 and Soluble intercellular adhesion molecule-1in ALVEOLI trial) and other variables (temperature, hemoglobin, sodium, glucose, bicarbonate, and pH) were also collected. All the longitudinal variables were obtained from Day 0 until death, ICU discharge, liberation from mechanical ventilation or Day 3 in the ICU, whichever occurred first.

The data in CDIC was extracted from the ICU Patient Data Management System which is used to collect patient health information, measurements of organ function parameters, results of laboratory tests and treatment parameters from ICU admission to discharge. We defined ΔP> 40 cmH_2_0, respiratory rates >60 (breaths min^-1^) and tidal volume > 20ml/Kg PBW as outliners, ﻿because these physiologically improbable values were assumed to be erroneous. We transformed outliers into missingness.

**C. Summary of the phenotype derivation.**

We first employed longitudinal LCA to identify phenotypes using time-dependent analysis with 24-hourly data from the first four days of invasive ventilation. Longitudinal LCA was fit to the combined datasets of candidate variables from all patients across Days 0, 1, 2 and 3, while allowing phenotype transition across ICU days. The optimal number of latent classes were selected using the lowest AIC, SABIC and highest values of entropy. LCA was performed using the tidyLPA package in R. We also applied group-based trajectory modeling (GBTM) on 24-hourly data from the first four days of invasive ventilation to identify trajectories for key variables between longitudinal phenotypes. GBTM was performed using the traj package in Stata.

**D. Group-based trajectory modeling (GBTM)**

GBTM is an application of finite mixture, which assumed that population is composed of a mixture of distinct groups defended by their developmental trajectories. In present study, GBTM was applied to longitudinal data of mechanical power or ventilatory ratio to fit individuals into latent clusters (or groups) based on the trajectories of daily mechanical power and ventilatory ratio during the first four days of invasive ventilation. The GBTM had three intrinsic assumptions in our study, first, an underlying censored normal distribution of mechanical power and ventilatory ratio; second, trajectories of mechanical power and ventilatory ratio that can be modeled as polynomials over time; third, the existence of distinct trajectory groups of mechanical power and ventilatory ratio. GBTM uses the maximum likelihood method to model the distinct trajectory of each group into unique polynomial functions, and the best-fit GBTM model was selected based on the Bayesian information criterion (BIC), which penalizes more complex models to ensure that a well-fitting yet parsimonious model is chosen, ﻿and the minimum number of patients should be over 5% of the entire study population. GBTM was performed using the traj package in Stata.

**E****. Approach to missing Data**

Missing data of class-defining variables were summarized in Table S3. Prior to longitudinal LCA and machine learning algorithms (XGBoost and GBM), we assumed that missing data was “missing at random”, We used multiple imputation by chained equation (MICE) which generated values for all missing data using the observed data for all patients. In total we imputed 5 different data sets (n = 5), the imputation method was weighted predictive mean matching. We used ‘mice’ package in R studio to impute the data (https://cran.rproject.org/web/packages/mice).

**F. Extreme gradient boosting (XGBoost)**

XGBoost combined with decision trees was employed to predict phenotypes. A classification tree was used as the weak learner, and the learning objective function was binary logistic. The boosting method works by iteratively refitting a weak classifier (decision tree) to residuals of previous models. Each successive classifier focused more on misclassified observations during the previous round of fitting. In our study, in order to identify the most important classifier variables, the importance of classifier variables was derived from XGBoost model (learning rate = 0.1, minimum loss reduction = 0, maximum tree depth =20, and number of trees = 250). We used ‘xgboost’ package (<https://cran.rproject.org/web/packages/xgboost>) in R studio to impute the data.

**G. Gradient boosted model (GBM)**

GBM is a machine learning algorithm that consecutively constructs new models and forms an ensemble of models to provide a more accurate estimate of the response variable. The principal idea is to construct the new base-learners to be maximally correlated with the negative gradient of the predefined loss function. In our study, regression tree was used as the base learner of the GBM. The initial settings included a bag fraction of 0.5, a tree complexity of 8, the number of tree was 300, and a learning rate of 0.01. We used GBM to generate the relative influence of class-defining variables. We used ‘gbm’ package (<https://cran.rproject.org/web/packages/gbm>) in R studio to impute the data.

**Table S1: Characteristics of Cohort and Clinical Trials**

|  | **Derivation Cohort** | **Validation Cohorts** | | | |
| --- | --- | --- | --- | --- | --- |
|  | CDIC | ALVEOLI | FACTT | EDEN | SAILS |
| No. of patients | 605 | 549 | 1000 | 1000 | 745 |
| Enrollment period | 2014-2021 | 1999-2002 | 2000-2005 | 2008-2011 | 2010-2013 |
| Inclusion criteria | Patients with ARDS and received invasive mechanical ventilation.  (Berlin definition) | Patients with ARDS and received invasive mechanical ventilation.  (AECC definition) | Patients with ARDS and received invasive mechanical ventilation.  (AECC definition) | Patients with ARDS and received invasive mechanical ventilation.  (AECC definition) | Patients with ARDS and received invasive mechanical ventilation.  (AECC definition) |
| Primary intervention | None | Lower PEEP vs Higher PEEP | Conservative vs Liberal Fluid Management | Initial Trophic vs Full Enteral Feeding | Rosuvastatin vs Placebo |
| Primary outcome | 28-day mortality | 60-day mortality | 60-day mortality | Ventilator-free days to 28 days | 60-day mortality |

**Table S2: Clinical variables for phenotyping in different datasets**

|  | **CDIC** | **ALVEOLI** | **FACTT** | **EDEN** | **SAILS** |
| --- | --- | --- | --- | --- | --- |
| Age | √ | √ | √ | √ | √ |
| Minute ventilation | √ | √ | √ | √ | √ |
| PEEP | √ | — | √ | √ | √ |
| Driving pressure | √ | √ | √ | √ | √ |
| Mechanical power | √ | √ | √ | √ | √ |
| ﻿Ventilatory ratio | √ | √ | √ | √ | √ |
| PaO_2_/FiO_2_ ratio | √ | √ | √ | √ | √ |
| Heart rate | √ | √ | √ | √ | √ |
| MAP | √ | √ | √ | √ | √ |
| pH | √ | √ | √ | √ | √ |
| Creatinine | √ | √ | √ | √ | √ |
| Bicarbonate | √ | √ | √ | √ | — |
| Lactate | √ | — | — | — | — |
| Fluid Balance | √ | √ | — | √ | √ |

PEEP= positive end-expiratory pressure; PaO2=partial pressure of oxygen; FiO2=fraction of inspired oxygen; MAP=mean arterial blood pressure.

*Lactate was missing in ARDSNet trials, and the longitudinal bicarbonate was missing in the SAILS trial. The ALVEOLI trial explored the impact of PEEP strategy on ARDS patients, and the FACTT explored the impact of fluid management strategy on ARDS patients. So, when performing the longitudinal LCA, we excluded PEEP and lactate in ALVEOLI trial, excluded lactate and fluid Balance in FACTT trial, excluded lactate in EDEN trial, and excluded bicarbonate and lactate in SAILS trial.

**Table S3:** **Percentage of missing data in the variables of interest**

|  | **CDIC** | **ALVEOLI** | **FACTT** | **EDEN** | **SAILS** |
| --- | --- | --- | --- | --- | --- |
| Age | 0% | 0% | 0% | 0% | 0% |
| Male | 0% | 0% | 0% | 0% | 0% |
| Height | 1.8% | 0% | 8.7% | 0.1% | 0% |
| Weight | 1.8% | 8.0% | 7.2% | 0.2% | 0.3% |
| PBW | 1.8% | 0% | 8.7% | 0.1% | 0% |
| BMI | 1.8% | 8.0% | 8.5% | 0.3% | 0.3% |
| ARDS Primary risk factor | 0% | 0% | 0% | 0% | 0% |
| SOFA score | 37.5% | — | — | — | — |
| APACHE II score | 17.2% | — | — | — | — |
| Severity of ARDS at baseline | 0% | 10% | 13.1% | 3.4% | 14.3% |
| Respiratory rate | 1.2% | 3.1% | 2.1% | 0.6% | 3.0% |
| Tidal volume | 1.8% | 8.6% | 8.7% | 12.5% | 9.6% |
| Minute ventilation | 4.0% | 3.8% | 4.8% | 12.5% | 10.5% |
| PEEP | 3.3% | 3.3% | 2.2% | 1.0% | 3.8% |
| Peak Pressure | 9.6% | 7.7% | 14.3% | 8.5% | 11.0% |
| Driving pressure | 10.9% | 10.7% | 14.6% | 21.8% | 19.5% |
| Mechanical power | 12.7% | 10.7% | 16.5% | 20.3% | 11.1% |
| Compliance | 11.6% | 11.3% | 16.1% | 12.5% | 10.5% |
| ﻿Ventilatory ratio | 1.8% | 11.5% | 15.8% | 15.3% | 18.1 |
| PaCO_2_ | 0.2% | 10.9% | 13.1% | 2.8% | 15.4% |
| PaO_2_/FiO_2_ ratio | 0% | 10.0% | 13.1% | 3.4% | 14.3% |
| Vasopressor use in the first 24h | 0% | 0% | 0% | 0% | 0% |
| Heart rate | 1.5% | 0.2% | 1.4% | 0% | 11.4% |
| MAP | 2.6% | 1.6% | 1.6% | 0.1% | 11.4% |
| Temperature | 7.1% | 0.2% | 1.6% | 0.1% | 11.7% |
| pH | 0% | 10.7% | 13.1% | 2.8% | 15.2% |
| BUN | 8.3% | 0.7% | 2.6% | — | 0.7% |
| Creatinine | 4.3% | 0.5% | 2.3% | 0.2% | 2.3% |
| Bicarbonate | 0.2% | 0.7% | 2.4% | 0.5% | 0.7% |
| Lactate | 0.3% | — | — | — | — |
| White blood cell count | 3.3% | 0.9% | 1.3% | 0.3% | 0.1% |
| Lymphocyte count | 4.1% | — | — | — | — |
| Platelet count | 5.4% | 1.5% | 0.8% | 0.5% | 0% |
| Hemoglobin | 3.3% | 0.7% | 2.3% | 1.9% | 2.1% |
| High-sensitive C-reactive protein | 32.1% | — | — | — | — |
| Interleukin-6 | 41.3% | 4.2% | — | — | — |

PBW=Predicted body weight; BMI= Body mass index; ARDS= acute respiratory distress syndrome; SOFA= Sequential organ failure assessment; APACHE= Acute Physiology and Chronic Health Evaluation II; PEEP= positive end-expiratory pressure; PaCO2=partial pressure of Carbon Dioxide; PaO2=partial pressure of oxygen; FiO2=fraction of inspired oxygen; MAP=mean arterial blood pressure; BUN= Blood urea nitrogen.

**Table S4: Metrics for choosing the best number of classes for longitudinal LCA in CIDC.**

| **Classes** | **AIC** | **SABIC** | **Entropy** | **prob_min** | **prob_max** | **n_min** | **n_max** | **BLRT_p** |
| --- | --- | --- | --- | --- | --- | --- | --- | --- |
| **CDIC (Full analysis)** | | | | | | | | |
| 2 | 85277 | 85385 | 0.848 | 0.898 | 0.974 | 0.222 | 0.778 | 0.01 |
| 3 | 83877 | 84023 | 0.906 | 0.874 | 0.976 | 0.091 | 0.733 | 0.01 |
| 4 | 83675 | 83859 | 0.789 | 0.841 | 0.912 | 0.041 | 0.47 | 0.01 |
| 5 | 79918 | 80140 | 0.797 | 0.81 | 1 | 0.001 | 0.418 | 0.01 |
| 6 | 81941 | 82201 | 0.836 | 0.727 | 0.975 | 0.029 | 0.439 | 0.01 |
| **CDIC (Sensitivity analysis*)** | | | | | | | | |
| 2 | 56049 | 56128 | 0.988 | 0.983 | 0.998 | 0.094 | 0.906 | 0.01 |
| 3 | 54631 | 54738 | 0.895 | 0.873 | 0.976 | 0.086 | 0.724 | 0.01 |
| 4 | 54322 | 54457 | 0.794 | 0.741 | 0.979 | 0.085 | 0.581 | 0.01 |
| 5 | 53710 | 53873 | 0.794 | 0.766 | 0.977 | 0.03 | 0.442 | 0.01 |
| 6 | 53071 | 53262 | 0.828 | 0.839 | 0.973 | 0.035 | 0.331 | 0.01 |

* For patients ﻿who remained on invasive mechanical ventilation for more than 96 hours in CDIC

**Table S5:** **Additional clinical characteristics at the start of mechanical ventilation (Day 0) in the CDIC**

|  | **All**  **(n=605)** | **Longitudinal phenotypes of ARDS** | | | **P value** |
| --- | --- | --- | --- | --- | --- |
|  |  | **Class 1**  **(n=400)** | **Class 2**  **(n=102)** | **Class 3**  **(n=103)** |  |
| Height (cm) | 170 (160-174.7) | 170 (160-174.5) | 170 (160-172) | 170 (160-175) | 0.18 |
| Weight (Kg) | 70 (60-75) | 70 (60-75) | 70 (60-70) | 65 (60-75) | 0.59 |
| PBW (Kg) | 65.8 (54.7-70.1) | 65.8 (56.3-69.9) | 65.8 (51.8-67.6) | 65.8 (54.1-70.3) | 0.68 |
| Comorbidities, n (%) | | | | | |
| Hypertension | 288 (47.6) | 204 (51) | 46 (45.1) | 38 (36.9) | 0.033 |
| Coronary heart disease | 97 (16.0) | 60 (15) | 20 (19.6) | 17 (16.5) | 0.52 |
| Chronic heart failure | 76 (12.6) | 52 (13) | 14 (13.7) | 10 (9.7) | 0.62 |
| COPD | 36 (5.9) | 21 (5.3) | 9 (8.8) | 6 (5.8) | 0.40 |
| Chronic renal diseases | 64 (10.6) | 40 (10) | 12 (11.8) | 12 (11.7) | 0.81 |
| Malignancy | 55 (9.1) | 33 (8.3) | 8 (7.8) | 14 (13.6) | 0.22 |
| Connective tissue disease | 16 (2.6)) | 6 (1.5) | 9 (8.8) | 1 (1.0) | <0.001 |
| Laboratory data in the first 24 h | | | | | |
| White blood cell count (10⁹ per L) | 11.1 (7.2-15.3) | 11.1 (7.3-15.0) | 12.8 (8.1-18.5) | 9.6 (5.5-13.6) | 0.0022 |
| Lymphocyte count (10⁹ per L) | 0.61 (0.38-0.95) | 0.60 (0.36-0.88) | 0.64 (0.41-1.09) | 0.67 (0.40-0.99) | 0.30 |
| Platelet count (10⁹ per L) | 144 (86-208) | 148 (89-209) | 151 (96-218) | 124 (70-185) | 0.074 |
| Hemoglobin (g/L) | 105 (88-123) | 106 (87-123) | 106 (90-121) | 101 (87-121) | 0.63 |
| High-sensitive C-reactive protein (mg/L) | 126 (63.5-194) | 115 (60.6-181.5) | 121 (47.1-205) | 166.4 (117-215) | 0.0082 |
| Interleukin-6 (pg/ml) | 181.2  (71.1-1093.5) | 153.3  (62.5-606.3) | 186.6  (79.5-556.5) | 1153.5  (225.9-5000) | <0.001 |
| ALT (U/L) | 37 (25-69.5) | 38 (24-67) | 40 (25.5-90.3) | 55 (34.5-118.5) | 0.47 |
| AST (U/L) | 55 (32.3-106.8) | 52.5 (31-98) | 65 (39.5-146.5) | 55 (34.5-118.5) | 0.064 |
| Albumin (g/L) | 29.4 (25-33) | 29.6 (25.3-33) | 29 (25.6-33) | 28.1 (23.2-32.3) | 0.22 |
| Total bilirubin (μmol/L) | 13.9 (8.7-24.4) | 13.7 (8.6-24) | 10.6 (7.1-18.2) | 18.7 (13.5-28.1) | 0.0072 |
| PT (s) | 13.7 (12.4-15.9) | 12.6 (12.3-15.7) | 13.3 (11.8-14.8) | 14.9 (13.3-17.5) | <0.001 |
| INR | 1.27 (1.15-1.48) | 1.26 (1.15-1.46) | 1.23 (1.1-1.36) | 1.38 (1.25, 1.61) | <0.001 |
| APTT (s) | 32.8 (28.9-38.2) | 32.2 (28.5-37.5) | 32 (30-39.2) | 34.8 (30.9-42.7) | 0.0041 |
| D-dimer (ng/ml) | 1946.5  (731-4446.5) | 1589.5  (650.5-3578.8) | 2461  (985.8-5939.8) | 2700.5  (942.5-4979.5) | 0.018 |
| Fib (g/L) | 4.33 (3.56-5.13) | 4.4 (3.62-5.12) | 4.11 (3.53-5.14) | 4.15 (3.46-5.11) | 0.34 |

PBW=Predicted body weight; COPD= Chronic obstructive pulmonary disease; ALT=Alanine transaminase; AST= Aspartate transaminase; PT=Prothrombin time; INR= International Normalized Ratio; APTT=Activated partial thromboplastin time; Fib= Fibrinogen.

**Table S6: Respiratory parameters and clinical outcomes for trajectories of ventilatory ratio and mechanical power in CDIC**

|  | ﻿**Ventilatory ratio trajectories** | | | P value | **﻿Mechanical power trajectories** | | | P value |
| --- | --- | --- | --- | --- | --- | --- | --- | --- |
|  | A  (n=150) | B  (n=342) | C  (n=113) |  | A  (n=131) | B  (n=377) | C  (n=97) |  |
| Age (years) | 63 (49-77) | 66.5 (55-77) | 65 (52-74) | 0.17 | 63 (51.5-74) | 67 (55-77) | 66 (53-75) | 0.17 |
| Male (gender), n (%) | 113 (75.3) | 239 (69.9) | 71 (62.8) | 0.091 | 76 (58) | 264 (70) | 83 (85.6) | <0.001 |
| **Parameters of mechanical ventilation in the first 24 h** | | | | | | | | |
| Respiratory rate (breaths min^-1^) | 23 (20-26) | 26 (23-30) | 30 (25-35) | < 0.001 | 22 (20-25.5) | 26 (23-30) | 32 (27-35) | <0.001 |
| Tidal volume (ml/kg PBW) | 7.3 (6.3-8.5) | 8.4 (7.3-9.7) | 9.2 (8.1-10.7) | < 0.001 | 7.8 (6.3-9.0) | 8.4 (7.3-9.7) | 8.9 (7.3-10.5) | < 0.001 |
| Minute ventilation (L/min) | 10.2 (7.8-11.6) | 13.4 (11.2-15.6) | 16.6 (13.2-19.3) | < 0.001 | 9.5 (7.6-11.0) | 13.1 (11.1-15.3) | 17.5 (14.7-20.4) | < 0.001 |
| PEEP (cmH_2_0) | 10 (6-11) | 9 (7-10) | 10 (8-12) | 0.063 | 8 (5.5-10) | 9 (7-10) | 10 (8-12) | < 0.001 |
| Peak Pressure (cmH_2_0) | 24 (21-27.75) | 24 (21-27) | 25 (22-29) | 0.12 | 22 (20-25.5) | 24 (21-26) | 28 (24-33) | < 0.001 |
| Driving pressure (cmH_2_0) | 15 (12-19) | 15 (12-18) | 15 (12-19) | 0.46 | 14 (12-17) | 15 (12-18) | 18 (14-21) | < 0.001 |
| Mechanical power (J/min) | 15.1 (12.2-19.0) | 20.9 (17.6-25.3) | 28.2 (23.0-35.3) | < 0.001 | 14.2 (11.8-16.4) | 20.9 (18.7-24.3) | 33.3 (28.9-37.2) | < 0.001 |
| Compliance (ml/cmH_2_0) | 30.3 (25.1-38.7) | 34.5 (27.4-44.6) | 33.9 (25.4-47.9) | 0.004 | 30.9 (26.2-40.4) | 34.8 (27.5-44.5) | 32.2 (23.3-44.6) | 0.016 |
| ﻿Ventilatory ratio | 1.28 (1.01-1.5) | 2 (1.66-2.39) | 3.03 (2.48-3.72) | < 0.001 | 1.37 (1.02-1.79) | 1.95 (1.53-2.43) | 2.72 (2.06-3.33) | < 0.001 |
| PaCO_2_ (mmHg) | 29.9 (26.2-35.1) | 33.9 (29.4-40.1) | 38.1 (33.3-48.5) | < 0.001 | 33.4 (28.3-38.55) | 33.9 (28.9-39.73) | 35.6 (28.4-41) | 0.46 |
| PaO_2_/FiO_2_ ratio (mmHg) | 153 (109-230) | 167 (126-210) | 144 (104-190) | 0.0092 | 163 (115.5-230) | 161 (124.5-210) | 142 (99.5-187.5) | 0.0052 |
| **Clinical Outcomes** | | | | | | | | |
| Alive and VFDs at Day 28 (days) | 6.7 (0-21.2) | 9.4 (0-22.3) | 0 (0-15.60) | 0.0032 | 15.8 (0-23.5) | 9.4 (0-21.6) | 0 (0-0) | < 0.001 |
| ICU mortality, n (%) | 44 (29.3) | 87 (25.4) | 45 (39.8) | 0.014 | 31 (23.7) | 93 (24.7) | 52 (53.6) | <0.001 |
| Hospital mortality, n (%) | 50 (33.3) | 93 (27.2) | 46 (40.7) | 0.022 | 35 (26.7) | 100 (26.5) | 54 (55.7) | <0.001 |
| 28-day mortality, n (%) | 58 (38.7) | 115 (33.6) | 55 (48.7) | 0.016 | 33 (25.2) | 130 (34.5) | 65 (67.0) | <0.001 |

PBW=Predicted body weight; PEEP= positive end-expiratory pressure; PaCO2=partial pressure of Carbon Dioxide; PaO2=partial pressure of oxygen; VFD=ventilator-free days; ICU=intensive care unit.

**Table S7: Interaction between** **longitudinal phenotypes of ARDS and time-varying mechanical power on ﻿28-day mortality in CDIC.**

|  | **HR (95% CI)** | **P value** |
| --- | --- | --- |
| Arterial pH | 0.055 (0.015-0.195) | <0.001 |
| PaCO_2_, mmHg | 0.98 (0.97-0.99) | 0.012 |
| PaO_2_/FiO_2_ ratio, (per 10 mmHg) | 0.82 (063-1.06) | 0.13 |
| Respiratory System Compliance, (per 10 ml/cmH_2_0) | 0.26 (0.19-0.37) | <0.001 |
| Mechanical power, J/min | 1.03 (1.01-1.05) | 0.00086 |
| **Longitudinal phenotypes (“Class 1” as reference)** | | |
| Class 2 | 0.37 (0.17-0.82) | 0.015 |
| Class 3 | 1.71 (0.91-3.23) | 0.097 |
| **﻿Interaction between phenotypes and time-varying mechanical power** | | |
| Mechanical power: Class 2 | 1.04 (1.01-1.07) | 0.0051 |
| Mechanical power: Class 3 | 0.99 (0.97-1.02) | 0.86 |

HR= hazard ratio; PaCO2=partial pressure of Carbon Dioxide; PaO_2_= partial pressure of oxygen; FiO_2_=fraction of inspired oxygen

**Table S8: Interaction between longitudinal phenotypes of ARDS and time-varying ventilatory ratio on ﻿28-day mortality in CDIC.**

|  | **HR (95% CI)** | **P value** |
| --- | --- | --- |
| Arterial pH | 0.034 (0.009-0.13) | <0.001 |
| PaCO_2_, mmHg | 0.97 (0.96-0.98) | <0.001 |
| PaO_2_/FiO_2_ ratio, (per 10 mmHg) | 0.75 (0.57-0.98) | 0.040 |
| Respiratory System Compliance, (per 10 ml/cmH_2_0) | 0.26 (0.18-0.37) | <0.001 |
| Ventilatory ratio | 1.16 (0.92-1.45) | 0.22 |
| **Longitudinal phenotypes (“Class 1” as reference)** | | |
| Class 2 | 2.12 (1.03-4.37) | 0.040 |
| Class 3 | 1.55 (0.81-2.96) | 0.60 |
| **﻿Interaction between phenotypes and time-varying mechanical power** | | |
| Ventilatory ratio: Class 2 | 0.93 (0.70-1.22) | 0.60 |
| Ventilatory ratio: Class 3 | 1.09 (0.81-1.46) | 0.56 |

HR= hazard ratio; PaCO2=partial pressure of Carbon Dioxide; PaO_2_= partial pressure of oxygen; FiO_2_=fraction of inspired oxygen

**Table S9: Interaction between longitudinal phenotypes of ARDS and time-varying driving pressure on ﻿28-day mortality in CDIC.**

|  | **HR (95% CI)** | **P value** |
| --- | --- | --- |
| Arterial pH | 0.077 (0.021-0.287) | 0.00013 |
| PaCO_2_, mmHg | 0.98 (0.97-0.99) | 0.0012 |
| PaO_2_/FiO_2_ ratio, (per 10 mmHg) | 0.71 (0.54-0.94) | 0.015 |
| Respiratory System Compliance, (per 10 ml/cmH_2_0) | 0.71 (0.47-1.07) | 0.10 |
| Driving pressure | 1.09 (1.06-1.13) | <0.001 |
| **Longitudinal phenotypes (“Class 1” as reference)** | | |
| Class 2 | 1.39 (0.70-2.75) | 0.34 |
| Class 3 | 2.73 (1.42-5.26) | 0.0026 |
| **﻿Interaction between phenotypes and time-varying mechanical power** | | |
| Driving pressure: Class 2 | 1.004 (0.97-1.04) | 0.77 |
| Driving pressure: Class 3 | 0.97 (0.94-1.003) | 0.075 |

HR= hazard ratio; PaCO2=partial pressure of Carbon Dioxide; PaO_2_=partial pressure of oxygen; FiO_2_=fraction of inspired oxygen

**Table S10: Quantitative lung CTs analysis between phenotypes on Day 0 in CDIC.**

|  | **Class 1**  **(n=328)** | **Class 2**  **(n=72)** | **Class 3**  **(n=27)** | **P value** |
| --- | --- | --- | --- | --- |
| Proportion of ﻿hyper-inflated lung volume, % | 5.3 (1.6-12.9) | 6.2 (1.8-13.9) | 5.2 (1.3-11.2) | 0.63 |
| ﻿Proportion of ﻿normally aerated lung volume, % | 55.5 (47.3-64.6) | 50.5 (38.3-60.7) | 54.7 (43.0-58.8) | 0.008 |
| ﻿Proportion of ﻿poorly-aerated lung volume, % | 17.1 (11.7-23.9) | 20.1 (15.5-27.6) | 19.7 (13.6-24.4) | 0.025 |
| ﻿﻿Proportion of ﻿non-aerated lung volume, % | 15.9 (9.1-24.6) | 16.8 (9.8-26.5) | 17.6 (12.6-26.4) | 0.50 |

**Table S11: Clinical characteristics at the start of mechanical ventilation (Day 0) and outcomes for the time-dependent phenotypes in the ALVEOLI.**

|  | All  (n=549) | Time-dependent phenotypes | | | P value |
| --- | --- | --- | --- | --- | --- |
|  |  | Class 1  (n=296) | Class 2  (n=79) | Class 3  (n=174) |  |
| Age (years) | 50 (39-65) | 53 (39-68) | 46 (37-61) | 48 (39-58) | 0.039 |
| Male (gender), n (%) | 302 (55.0) | 153 (51.7) | 38 (48.1) | 111 (63.8) | 0.016 |
| BMI (kg/m^2^) | 26.7 (22.5-30.7) | 26.6 (22.2-30.4) | 27.9 (23.3-33.6) | 26.5 (22.8-30.4) | 0.12 |
| ARDS Primary risk factor, n (%) | | | | | 0.0020 |
| Pneumonia | 221 (40.3) | 107 (36.2) | 26 (32.9) | 88 (50.6) |  |
| Sepsis | 120 (21.9) | 60 (20.3) | 17 (21.5) | 43 (24.7) |  |
| Aspiration | 84 (15.3) | 56 (18.9) | 12 (15.2) | 16 (9.2) |  |
| Other | 124 (22.6) | 73 (24.7) | 24 (30.4) | 27 (15.5) |  |
| Severity of ARDS at baseline, n (%) | | | | | <0.001 |
| Mild | 100 (20.2) | 77 (30.0) | 5 (6.9) | 18 (10.9) |  |
| Moderate | 288 (58.3) | 156 (60.7) | 39 (54.2) | 93 (56.4) |  |
| Severe | 106 (21.5) | 24 (9.3) | 28 (38.9) | 54 (32.7) |  |
| Parameters of mechanical ventilation in the first 24 h | | | | | |
| Respiratory rate (breaths min^-1^) | 29 (23.8-35) | 24 (20-28) | 35 (27.3-35) | 35 (30-35) | < 0.001 |
| Tidal volume (ml/kg PBW) | 6.0 (5.9-6.1) | 6.0 (6.0-6.1) | 6.0 (5.9-6.9) | 6.0 (5.9-6.1) | 0.28 |
| Minute ventilation (L/min) | 11.5 (9.3-13.7) | 9.6 (8.4-11.1) | 12.7 (10.6-14.8) | 14.1 (12.7-16.4) | <0.001 |
| PEEP (cmH_2_0) | 12 (8-14) | 10 (8-14) | 10 (8-14) | 14 (10-16) | <0.001 |
| Peak Pressure (cmH_2_0) | 32 (26-37) | 28 (23-32) | 40 (35.75-45) | 35 (31-40) | <0.001 |
| Driving pressure (cmH_2_0) | 13 (10-16) | 11 (9-14) | 22 (20-25) | 13 (11-16) | <0.001 |
| Mechanical power (J/min) | 27.4 (20.1-37.9) | 20.9 (16.6-25.7) | 35.0 (26.0-44.1) | 40.5 (33.8-45.0) | <0.001 |
| Compliance (ml/cmH_2_0) | 30 (22.6-39.9) | 34.4 (27.3-44.6) | 17.3 (14.7-19.3) | 29.9 (24.7-37.0) | <0.001 |
| ﻿Ventilatory ratio | 1.91 (1.5-2.42) | 1.57 (1.32-1.88) | 2.52 (2.05-3.07) | 2.35 (1.95-2.68) | <0.001 |
| PaCO_2_ (mmHg) | 39 (34-45) | 38 (34-43) | 45 (38-54) | 40 (34-44) | <0.001 |
| PaO_2_/FiO_2_ ratio (mmHg) | 142 (104.3-187) | 163 (123-210) | 116 (79-168) | 120 (86-160) | <0.001 |
| Vasopressor use in the first 24h, n (%) | 113 (21.04) | 35 (12.11) | 20 (25.32) | 58 (34.32) | <0.001 |
| Vital signs in the first 24 h | | | | | |
| Heart rate (beats min^-1^) | 97.8 (18.4) | 93.5 (17.6) | 103.2(18.0) | 102.7 (18.2) | <0.001 |
| MAP (mmHg) | 78.7 (70-89.1) | 80.7 (71.7-91.2) | 81.7 (70.3-88.3) | 73.5 (67.1-84.6) | <0.001 |
| Temperature (℃) | 37.5 (36.9-38.1) | 37.4 (36.9-37.9) | 37.6 (36.9-38.1) | 37.7 (37.1-38.3) | <0.001 |
| Laboratory data in the first 24 h | | | | | |
| pH | 7.39 (7.32-7.43) | 7.41 (7.37-7.44) | 7.35 (7.27-7.4) | 7.35 (7.29-7.41) | <0.001 |
| BUN (mg/dl) | 20 (13-34) | 19 (13-33) | 17 (12-28.5) | 22 (14-40.5) | 0.041 |
| Creatinine (mg/dL) | 1 (0.72-1.7) | 1 (0.7-1.8) | 1 (0.8-1.6) | 1 (0.7-1.7) | 0.86 |
| Bicarbonate (mmol/L) | 23 (20-26) | 24 (21-27) | 26 (21-29) | 21 (17-25) | <0.001 |
| Platelet count (10⁹ per L) | 149 (77-230.5) | 151.5 (88-230) | 168 (77-247.75) | 136 (56.5-222.5) | 0.16 |
| IL-6 (pg/ml) | 244 (94-752) | 163.5  (75.8-404.8) | 337  (118.75-1264) | 464  (140.5-1470.5) | <0.001 |
| sICAM (ng/ml) | 923.9  (604.4-1384.1) | 787.2  (543-1216.2) | 948.6  (676-1378.8) | 1098  (765-1634.7) | <0.001 |
| Fluid balance in the first 24h (L) | 1.88 (0.51-3.68) | 1.51 (0.27-2.82) | 2.23 (1.03-4.75) | 2.49 (0.81-4.60) | <0.001 |
| Use of NMBAs, n (%) | 113 (21.0) | 35 (12.1) | 20 (25.3) | 58 (34.3) | <0.001 |
| Alive and VFDs at Day 28 (days) | 19 (0-23) | 22 (12.5-24) | 14 (0-20) | 13 (0-20) | <0.001 |
| 28-day mortality, n (%) | 125 (22.77) | 49 (16.55) | 23 (29.11) | 53 (30.46) | <0.001 |
| 60-day mortality, n (%) | 144 (26.2) | 56 (18.9) | 26 (32.9) | 62 (35.6) | <0.001 |

BMI= Body mass index; ARDS= acute respiratory distress syndrome; PBW=Predicted body weight; PEEP= positive end-expiratory pressure; PaCO2=partial pressure of Carbon Dioxide; PaO2=partial pressure of oxygen; FiO_2_=fraction of inspired oxygen; MAP=mean arterial blood pressure; BUN= Blood urea nitrogen; IL-6= ﻿Interleukin-6; sICAM= ﻿Soluble intercellular adhesion molecule-1; NMBAs= neuromuscular blocking agents.

**Table S12: Clinical characteristics at the start of mechanical ventilation (Day 0) and outcomes for the time-dependent phenotypes in the FACTT.**

|  | All  (n=993) | Time-dependent phenotypes | | | P value |
| --- | --- | --- | --- | --- | --- |
|  |  | Class 1  (n=548) | Class 2  (n=347) | Class 3  (n=98) |  |
| Age (years) | 49.0 (38.0-61.0) | 50.0 (39.8-61.0) | 46.0(36.0-58.0) | 53.5 (43.2-68.0) | <0.001 |
| Male (gender), n (%) | 463 (46.6) | 245 (44.7) | 179 (51.6) | 39 (39.8) | 0.048 |
| BMI (kg/m^2^) | 27.4 (23.1- 32.7) | 27.4 (23.5- 32.2) | 27.4 (22.8- 33.2) | 26.9 (23.0- 33.8) | 0.85 |
| ARDS Primary risk factor, n (%) | | | | | <0.001 |
| Pneumonia | 469 (47.2) | 237 (43.2) | 186 (53.6) | 46 (46.9) |  |
| Sepsis | 232 (23.4) | 108 (19.7) | 84 (24.2) | 40 (40.8) |  |
| Aspiration | 149 (15.0) | 104 (19.0) | 38 (11.0) | 7 (7.1) |  |
| Other | 143 (14.4) | 99 (18.1) | 39 (11.2) | 5 (5.1) |  |
| Charlson comorbidity index | 0.00 (0.00-2.00) | 0.00 (0.00-2.00) | 0.00 (0.00-2.00) | 2.00 (0.00-2.75) | 0.004 |
| Severity of ARDS at baseline, n (%) | | | | | <0.001 |
| Mild | 241 (27.9) | 185 (40.0) | 33 (10.4) | 23 (27.4) |  |
| Moderate | 461 (53.4) | 250 (54.1) | 166 (52.4) | 45 (53.6) |  |
| Severe | 161 (18.7) | 27 (5.8) | 118 (37.2) | 16 (19.0) |  |
| Parameters of mechanical ventilation in the first 24 h | | | | | |
| Respiratory rate (breaths min^-1^) | 28.0 (23.0-35.0) | 24.0 (20.0-28.0) | 35.0 (30.0-35.0) | 30.5 (27.3-35.0) | <0.001 |
| Tidal volume (ml/kg PBW) | 6.03 (5.96-6.56) | 6.03 (5.96-6.34) | 6.03 (5.95-6.73) | 6.03 (5.95-6.97) | 0.20 |
| Minute ventilation (L/min) | 11.5 (9.4-14.0) | 10.2 (8.4-12.0) | 13.6 (11.4-15.6) | 14.0 (11.3-16.8) | <0.001 |
| PEEP (cmH_2_0) | 8.00 (5.0-10.0) | 8.0 (5.0-10.0) | 12.0 (10.0-14.0) | 8.0 (5.0-12.0) | <0.001 |
| Plate Pressure (cmH_2_0) | 24.0 (20.0-29.0) | 21.0 (18.0-25.0) | 28.0 (25.0-32.0) | 25.0 (20.0-29.0) | <0.001 |
| Driving pressure (cmH_2_0) | 15.0 (12.0-18.0) | 14.0 (11.0-17.0) | 16.0 (13.0-20.0) | 15.0 (13.0-18.0) | <0.001 |
| Mechanical power (J/min) | 24.5 (17.3-33.4) | 18.8 (14.0-24.5) | 32.6 (26.5-39.3) | 31.5 (20.7-42.3) | <0.001 |
| Compliance (ml/cmH_2_0) | 27.3 (20.8-35.0) | 29.6 (22.2-36.8) | 24.1 (18.8-31.3) | 28.7 (21.2-34.5) | <0.001 |
| ﻿Ventilatory ratio | 2.00 (1.59-2.47) | 1.68 (1.39-1.99) | 2.52 (2.19-3.06) | 2.06 (1.63-2.47) | <0.001 |
| PaCO_2_ (mmHg) | 41.0 (35.0-48.0) | 39.0 (34.0-45.0) | 45.0 (38.0-54.0) | 37.5 (32.0-42.3) | <0.001 |
| PaO_2_/FiO_2_ ratio (mmHg) | 154 (112-208) | 183 (144-230) | 115 (86-153) | 155 (110-201) | <0.001 |
| Vasopressor use in the first 24h, n (%) | 328 (33.0) | 138 (25.2) | 141 (40.6) | 49 (50.0) | <0.001 |
| Vital signs in the first 24 h | | | | | |
| Heart rate (beats min^-1^) | 98.0 (84.0-111.0) | 94.0 (80.0-107.0) | 105.0(91.0-116.0) | 100.5 (84.0-115.5) | <0.001 |
| MAP (mmHg) | 75 (68-86) | 78 (70-89) | 73 (66-84) | 72 (66-82) | <0.001 |
| Temperature (℃) | 37.5 (36.9-38.1) | 37.5 (36.9-38.0) | 37.6 (37.0-38.2) | 37.3 (36.6-38.1) | 0.057 |
| Laboratory data in the first 24 h | | | | | |
| pH | 7.38 (7.31-7.43) | 7.42 (7.37-7.45) | 7.33 (7.27-7.38) | 7.30 (7.25-7.34) | <0.001 |
| BUN (mg/dl) | 19.0 (12.5-31.5) | 17.0 (11.0-25.0) | 20.0 (13.0-30.0) | 60.0 (47.0-75.5) | <0.001 |
| Creatinine (mg/dL) | 1.00 (0.70-1.60) | 0.90 (0.70-1.20) | 1.10 (0.80-1.60) | 3.35 (2.82-4.30) | <0.001 |
| Bicarbonate (mmol/L) | 24.0 (20.0-27.0) | 25.0 (22.0-28.0) | 23.0 (19.0-27.0) | 17.0 (15.0-21.0) | <0.001 |
| Fluid balance in the first 24h (L) | 1.48 (-0.17-3.50) | 0.83 (-0.70-2.52) | 2.33 (0.77-4.17) | 3.90 (1.35-6.67) | <0.001 |
| Alive and VFDs at Day 28 (days) | 18 (0-23) | 21 (14-24) | 9 (0-19) | 3 (0-21) | <0.001 |
| 28-day mortality, n (%) | 231 (23.3) | 79 (14.4) | 112 (32.3) | 40 (40.8) | <0.001 |
| 60-day mortality, n (%) | 267 (26.9) | 97 (17.7) | 123 (35.4) | 47 (47.9) | <0.001 |

BMI= Body mass index; ARDS= acute respiratory distress syndrome; PBW=Predicted body weight; PEEP= positive end-expiratory pressure; PaCO2=partial pressure of Carbon Dioxide; PaO2=partial pressure of oxygen; FiO_2_= fraction of inspired oxygen; MAP=mean arterial blood pressure; BUN= Blood urea nitrogen.

**Table S13: Clinical characteristics at the start of mechanical ventilation (Day 0) and outcomes for the time-dependent phenotypes in the EDEN.**

|  | All  (n=1000) | Time-dependent phenotypes | | | P value |
| --- | --- | --- | --- | --- | --- |
|  |  | Class 1  (n=418) | Class 2  (n=211) | Class 3  (n=371) |  |
| Age (years) | 52 (42-63) | 53 (43-64) | 47 (34-55) | 55 (45-66) | < 0.001 |
| Male (gender), n (%) | 510 (51) | 204 (48.8) | 120 (56.9) | 186 (50.1) | 0.15 |
| BMI (kg/m^2^) | 28.8 (24.0-34.8) | 28.3 (23.7-33.1) | 30.01 (25.0-38.1) | 28.9 (23.9-35.5) | < 0.001 |
| ARDS Primary risk factor, n (%) | | | | | 0.096 |
| Pneumonia | 650 (65) | 263 (62.9) | 135 (64) | 252 (67.9) |  |
| Sepsis | 147 (14.7) | 54 (12.9) | 39 (18.5) | 54 (14.6) |  |
| Aspiration | 96 (9.6) | 48 (11.5) | 14 (6.6) | 34 (9.2) |  |
| Other | 107 (10.7) | 53 (12.7) | 23 (10.9) | 31 (8.4) |  |
| Severity of ARDS at baseline, n (%) | | | | | <0.001 |
| Mild | 232 (24) | 160 (40.7) | 28 (13.5) | 44 (12) |  |
| Moderate | 495 (51.2) | 194 (49.4) | 103 (49.8) | 198 (54.1) |  |
| Severe | 239 (24.7) | 39 (9.9) | 76 (36.7) | 124 (33.9) |  |
| Parameters of mechanical ventilation in the first 24 h | | | | | |
| Respiratory rate (breaths min^-1^) | 25 (20-30) | 20 (17-25) | 33 (29-37) | 26 (22-30) | < 0.001 |
| Tidal volume (ml/kg PBW) | 6.06 (5.96-6.77) | 6.06 (5.96-6.86) | 6.06 (5.97-6.85) | 6.06 (5.96-6.61) | 0.74 |
| Minute ventilation (L/min) | 9.99 (8-12) | 8.14 (7-9.56) | 13.5 (12.1-15.23) | 10.2 (8.64-11.49) | < 0.001 |
| PEEP (cmH_2_0) | 10 (5-12) | 8 (5-10) | 10 (9.5-14) | 10 (8-12) | <0.001 |
| Plate Pressure (cmH_2_0) | 23 (19-27) | 20 (17-24.75) | 26 (22-29) | 24 (20-28) | < 0.001 |
| Driving pressure (cmH_2_0) | 13 (10-17) | 13 (10-16) | 14 (11-18) | 13 (10-17) | 0.008 |
| Mechanical power (J/min) | 20.3 (14.5-27.8) | 14.8 (10.9-18.8) | 35.6 (30.4-42.1) | 21.0 (16.8-25.5) | < 0.001 |
| ﻿Ventilatory ratio | 1.61 (1.29-2.07) | 1.34 (1.1-1.63) | 2.21 (1.75-2.66) | 1.7 (1.41-2.12) | < 0.001 |
| PaCO_2_ (mmHg) | 38 (34-45) | 37 (33-43) | 38 (32.25-44) | 40 (34-48) | < 0.001 |
| PaO_2_/FiO_2_ ratio (mmHg) | 138 (100-196) | 177 (131-233) | 114 (85-168) | 118 (89-162) | < 0.001 |
| Vasopressor use in the first 24h, n (%) | 383 (38.3) | 119 (28.5) | 83 (39.3) | 181 (48.8) | <0.001 |
| Vital signs in the first 24 h | | | | | |
| Heart rate (beats min^-1^) | 94 (81-108) | 91 (79.25-104) | 100 (86-114) | 94 (80-108) | < 0.001 |
| MAP (mmHg) | 75.7 (68.7-84) | 78.2 (70.1-87.6) | 76.7 (68.7-84.7) | 73 (67-80.3) | < 0.001 |
| Temperature (℃) | 37.3 (36.8-37.9) | 37.2 (36.7-37.8) | 37.3 (36.9-38.1) | 37.3 (36.8-37.9) | 0.099 |
| Laboratory data in the first 24 h | | | | | |
| pH | 7.36 (7.3-7.42) | 7.41 (7.36-7.44) | 7.35 (7.28-7.4) | 7.32 (7.27-7.37) | < 0.001 |
| Creatinine (mg/dL) | 1.2 (0.8-2) | 1.1 (0.7-1.7) | 1.3 (0.85-2.2) | 1.4 (0.9-2.3) | < 0.001 |
| Bicarbonate (mmol/L) | 22 (19-26) | 23 (20-26) | 22 (19-26) | 21 (18.5-24) | < 0.001 |
| Fluid balance in the first 24h (L) | 2.04 (0.43-3.95) | 1.27 (-0.05-2.92) | 2.57 (1.03-4.32) | 2.75 (1.01-4.69) | <0.001 |
| Alive and VFDs at Day 28 (days) | 21 (7-24) | 23 (18-25) | 18 (0-22) | 18 (0-23) | < 0.001 |
| 28-day mortality, n (%) | 194 (19.4) | 61 (14.6) | 46 (21.8) | 87 (23.5) | 0.004 |
| 60-day mortality, n (%) | 227 (22.7) | 76 (18.2) | 51 (24.2) | 100 (27) | 0.011 |
| 90-day mortality, n (%) | 233 (23.3) | 77 (18.4) | 52 (24.6) | 104 (28) | 0.005 |

BMI= Body mass index; ARDS= acute respiratory distress syndrome; PBW=Predicted body weight; PEEP= positive end-expiratory pressure; PaCO2=partial pressure of Carbon Dioxide; PaO2=partial pressure of oxygen; FiO_2_= fraction of inspired oxygen; MAP=mean arterial blood pressure; VFDs=ventilator free days.

**Table S14: Clinical characteristics at the start of mechanical ventilation (Day 0) and outcomes for the time-dependent phenotypes in the** **SAILS.**

|  | All  (n=745) | Time-dependent phenotypes | | | P value |
| --- | --- | --- | --- | --- | --- |
|  |  | Class 1  (n=462) | Class 2  (n=182) | Class 3  (n=101) |  |
| Age (years) | 55 (42-66) | 57 (45-67) | 52 (40-63) | 53 (37-62) | < 0.001 |
| Male (gender), n (%) | 365 (48.99) | 210 (45.45) | 115 (63.19) | 40 (39.6) | <0.001 |
| BMI (kg/m^2^) | 28.6 (23.8-34.6) | 28.4 (23.7-34.4) | 28.6 (24.0-34.9) | 30.5 (24.6-35.8) | 0.28 |
| ARDS Primary risk factor, n (%) | | | | | 0.24 |
| Pneumonia | 529 (71.01) | 329 (71.21) | 121 (66.48) | 79 (78.22) |  |
| Sepsis | 145 (19.46) | 87 (18.83) | 44 (24.18) | 14 (13.86) |  |
| Aspiration | 49 (6.58) | 31 (6.71) | 14 (7.69) | 4 (3.96) |  |
| Other | 22 (2.95) | 15 (3.25) | 3 (1.65) | 4 (3.96) |  |
| Severity of ARDS at baseline, n (%) | | | | | <0.001 |
| Mild | 152 (26.95) | 124 (37.13) | 26 (17.81) | 2 (2.38) |  |
| Moderate | 305 (54.08) | 180 (53.89) | 85 (58.22) | 40 (47.62) |  |
| Severe | 107 (18.97) | 30 (8.98) | 35 (23.97) | 42 (50) |  |
| Parameters of mechanical ventilation in the first 24 h | | | | | |
| Respiratory rate (breaths min^-1^) | 25 (20-30) | 22 (18-26) | 32 (28-35) | 28 (23-32) | < 0.001 |
| Tidal volume (ml/kg PBW) | 6.06 (5.96-6.65) | 6.06 (5.96-6.73) | 6.03 (5.97-6.61) | 6.04 (5.91-6.51) | 0.58 |
| Minute ventilation (L/min) | 10.3 (8.3-12.4) | 8.9 (7.5-10.6) | 13.95 (12.57-15.4) | 10.6 (9.65-12) | < 0.001 |
| PEEP (cmH_2_0) | 8 (5-10) | 8 (5-10) | 8 (6-10) | 14 (12-16) | < 0.001 |
| Plate Pressure (cmH_2_0) | 22 (18-27) | 20 (16-24) | 24 (21-28) | 26 (21.5-29.5) | < 0.001 |
| Driving pressure (cmH_2_0) | 13 (10-17) | 12 (9-16) | 16 (12-20) | 12 (8-16) | < 0.001 |
| Mechanical power (J/min) | 20.6 (14.4-28.0) | 15.2 (11.5-19.8) | 30.2 (25.8-38.0) | 23.7 (21.0-29.0) | < 0.001 |
| ﻿Ventilatory ratio | 1.75 (1.39-2.24) | 1.53 (1.22-1.85) | 2.09 (1.72-2.53) | 2.2 (1.8-2.67) | < 0.001 |
| PaCO_2_ (mmHg) | 39 (34-45) | 39 (34-45) | 37 (32-43) | 45 (38-53.5) | < 0.001 |
| PaO_2_/FiO_2_ ratio (mmHg) | 153 (108-203.3) | 178 (131-227.8) | 140.5 (101.3-180) | 99.5 (76.3-120) | < 0.001 |
| Vasopressor use in the first 24h, n (%) | 320 (43.78) | 169 (36.98) | 96 (53.93) | 55 (57.29) | <0.001 |
| Vital signs in the first 24 h | | | | | |
| Heart rate (beats min^-1^) | 94 (81-106) | 91 (80-103) | 99 (86-113) | 95 (80.75-108) | < 0.001 |
| MAP (mmHg) | 77.7 (70-87.3) | 79 (71-90) | 78 (70-86.6) | 72 (67-79.3) | < 0.001 |
| Temperature (℃) | 37.1 (36.7-37.7) | 37.1 (36.7-37.7) | 37.2 (36.7-37.8) | 37.1 (36.7-37.5) | 0.29 |
| Laboratory data in the first 24 h | | | | | |
| pH | 7.38 (7.33-7.43) | 7.39 (7.34-7.44) | 7.38 (7.32-7.45) | 7.35 (7.27-7.4) | < 0.001 |
| Creatinine (mg/dL) | 1.1 (0.8-1.0) | 1.2 (0.9-1.8) | 1.1 (0.6-2) | 1.1 (0.7-1.83) | 0.009 |
| Bicarbonate (mmol/L) | 21 (18-24) | 20 (17-24) | 22 (19-25) | 21 (18-25) | <0.001 |
| Fluid balance in the first 24h (L) | 1.23 (-0.44-2.87) | 0.99 (-0.62-2.43) | 1.54 (-0.08-3.30) | 2.06 (0.55-3.42) | < 0.001 |
| Alive and VFDs at Day 28 (days) | 20 (8-24) | 22 (13-24) | 18 (7.25-22) | 16 (0-21) | < 0.001 |
| 28-day mortality, n (%) | 172 (23.09) | 96 (20.78) | 42 (23.08) | 34 (33.66) | 0.021 |
| 60-day mortality, n (%) | 199 (26.7) | 112 (24.2) | 50 (27.5) | 37 (36.6) | 0.037 |
| 90-day mortality, n (%) | 205 (27.52) | 113 (24.46) | 53 (29.12) | 39 (38.61) | 0.013 |

BMI= Body mass index; ARDS= acute respiratory distress syndrome; PBW=Predicted body weight; PEEP= positive end-expiratory pressure; PaCO2=partial pressure of Carbon Dioxide; PaO2=partial pressure of oxygen; FiO_2_= fraction of inspired oxygen; MAP=mean arterial blood pressure; VFDs=ventilator free days.

**Table S15: Multinomial logistical regression models for the prediction of phenotypes in CDIC.**

|  | Class 2 vs Class 1 | | Class 3 vs Class 1 | |
| --- | --- | --- | --- | --- |
|  | OR (95% CI) | P value | OR (95% CI) | P value |
| Phenotypes on Day 0 (Model A) | | | | |
| ﻿Ventilatory ratio | 14.3 (7.52-27.36) | <0.001 | 1.18 (0.76-1.85) | 0.45 |
| Mechanical power | 1.05 (1.00-1.11) | 0.049 | 1.07 (1.03-1.13) | 0.001 |
| Respiratory rate | 1.28 (1.18-1.38) | <0.001 | 1.10 (1.04-1.16) | <0.001 |
| Arterial pH, per 0.1 | 0.58 (0.52-0.65) | <0.001 | 0.5 (0.40-0.63) | <0.001 |
| Phenotypes on Day 2 (Model B) | | | | |
| ﻿Ventilatory ratio | 1.87 (1.36-2.59) | <0.001 | 0.99 (0.54-1.80) | 0.96 |
| Mechanical power | 1.08 (1.04-1.12) | <0.001 | 1.08 (1.02-1.14) | 0.010 |
| Creatinine | 0.998 (0.994-1.001) | 0.19 | 1.002 (0.999-1.006) | 0.22 |

OR=odds ratio; PaCO2=partial pressure of Carbon Dioxide

**Table S16:** **The predictive value of parsimonious probabilistic models**

|  | **AUROC** | | |
| --- | --- | --- | --- |
|  | Class 1 | Class 2 | Class 3 |
| Phenotypes on Day 0 (Model A) | | | |
| CDIC | 0.86 (0.82-0.90) | 0.97 (0.95-0.98) | 0.67 (0.62-0.73) |
| ALVEOLI | 0.96 (0.94-0.98) | 0.76 (0.70-0.82) | 0.93 (0.91-0.95) |
| FACTT | 0.95 (0.94-0.97) | 0.94 (0.92-0.95) | 0.80 (0.76-0.85) |
| EDEN | 0.93 (0.92-0.95) | 0.98 (0.97-0.99) | 0.89 (0.87-0.91) |
| SAILS | 0.92 (0.90-0.94) | 0.90 (0.87-0.93) | 0.82 (0.79-0.86) |
| Phenotypes on Day 2 (Model B) | | | |
| CDIC | 0.78 (0.72-0.83) | 0.80 (0.74-0.86) | 0.70 (0.60-0.79) |
| ALVEOLI | 0.79 (0.75-0.84) | 0.76 (0.72-0.81) | 0.65 (0.59-0.72) |
| FACTT | 0.84 (0.82-0.97) | 0.93 (0.90-0.96) | 0.81 (0.79-0.84) |
| EDEN | 0.75 (0.71-0.79) | 0.81 (0.79-0.84) | 0.63 (0.59-0.68) |
| SAILS | 0.71 (0.67-0.76) | 0.83 (0.81-0.86) | 0.66 (0.59-0.74) |

AUROC= area under the receiver operating characteristic curve.

**Figure S1: Example of original lung CT (A), lung segmentations (B) and** **CT attenuation value segmentation results (C).**

In Figure S1C, different color represents the different inflated status of lung parenchyma, with green indicate the over inflated lung tissue, and red indicate the non-aerated lung tissue.

**Figure S2: Patients selection on the CDIC cohort.**

**Figure S3: Trajectory of variables overtime between phenotypes in CDIC cohort (mechanical power, ventilatory ratio, PaO_2_/FiO_2_, serum lactate, serum creatinine, serum bicarbonate).**

**
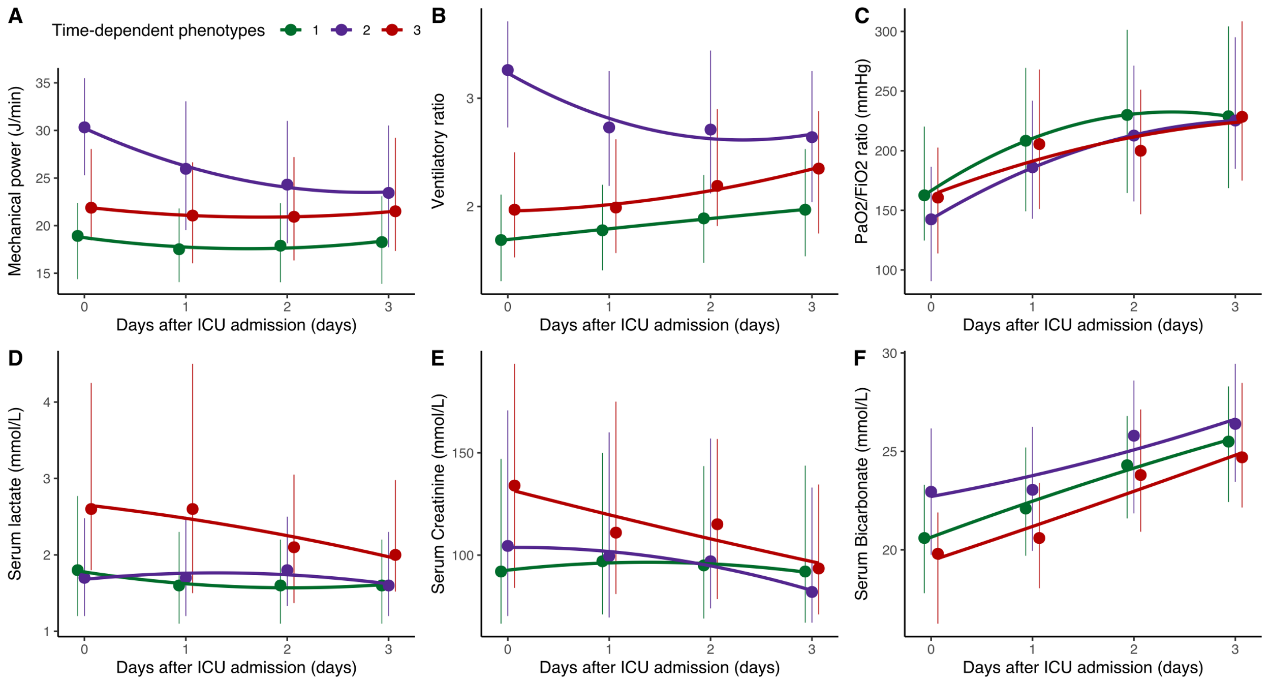
**

PaO_2_=partial pressure of oxygen; FiO_2_= fraction of inspired oxygen.

**Figure S4:** **Trajectory of variables overtime between phenotypes in CDIC cohort (driving pressure, PEEP, Respiratory rate).**

**
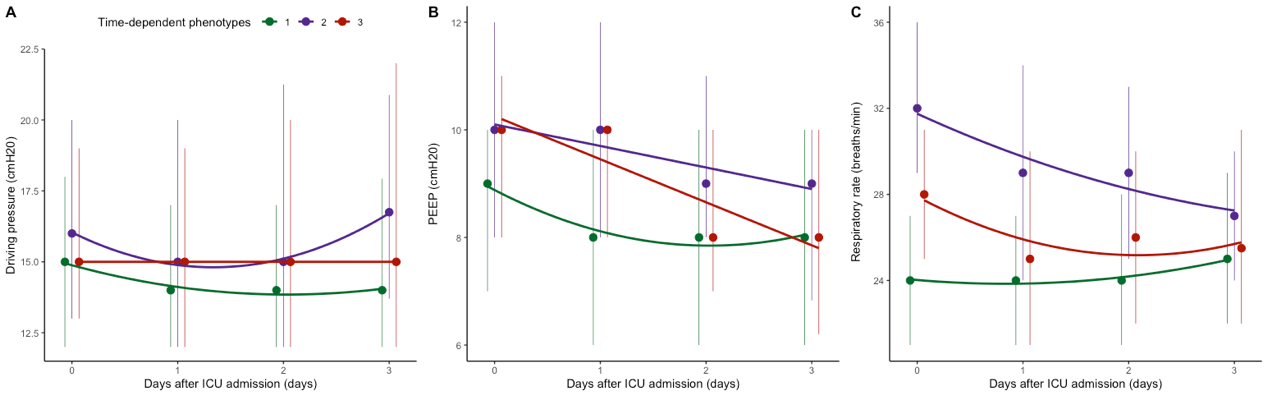
**

PEEP= positive end-expiratory pressure.

**Figure S5: Trajectory of variables overtime between phenotypes in CDIC cohort (pH, PaCO2, Platelets, D-dimer, Blood urea nitrogen, Bilirubin, hs-CRP, MAP).**

**
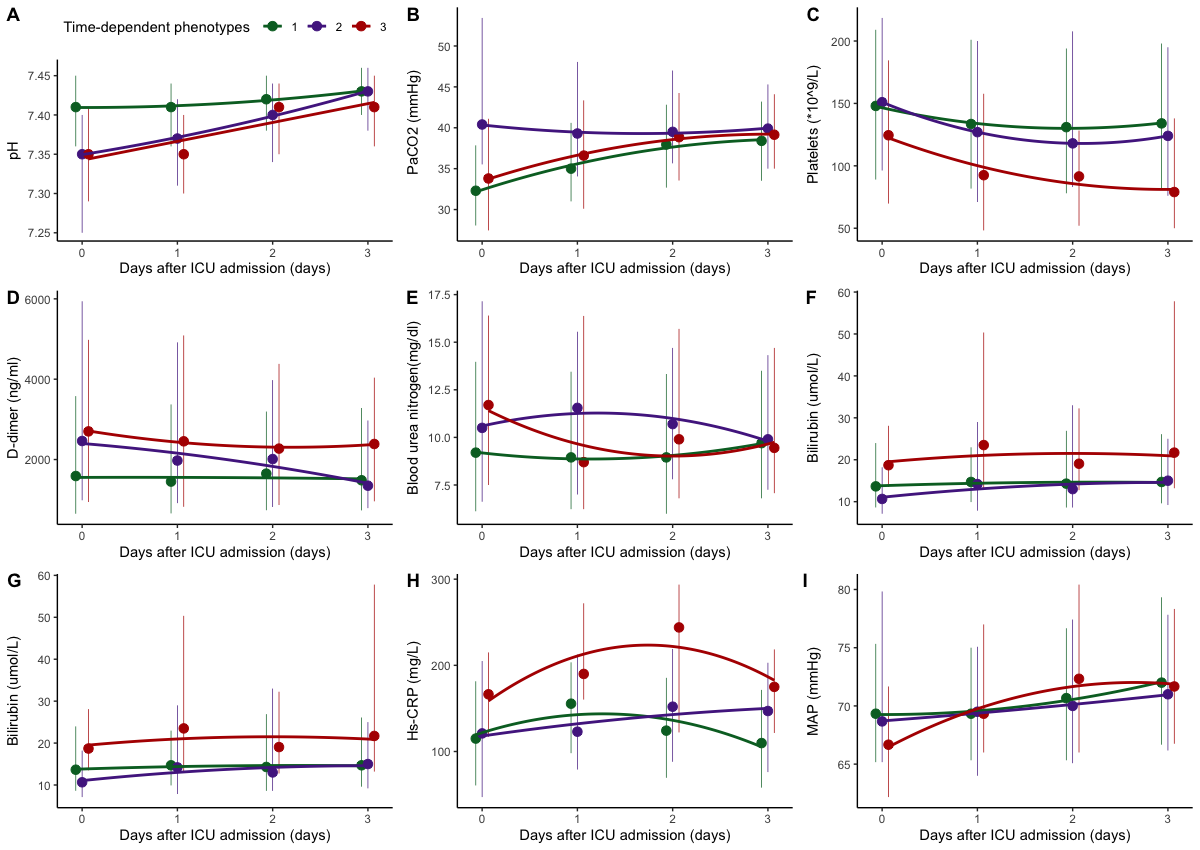
**

PaCO_2_=partial pressure of Carbon Dioxide; MAP=mean arterial blood pressure; Hs-CRP: High-sensitive C-reactive protein

**Figure S6: Comparisons of inflammation markers between phenotypes (IL-6 in CDIC cohort and ALVEOLI trial, sICAM in ALVEOLI trial).**

**
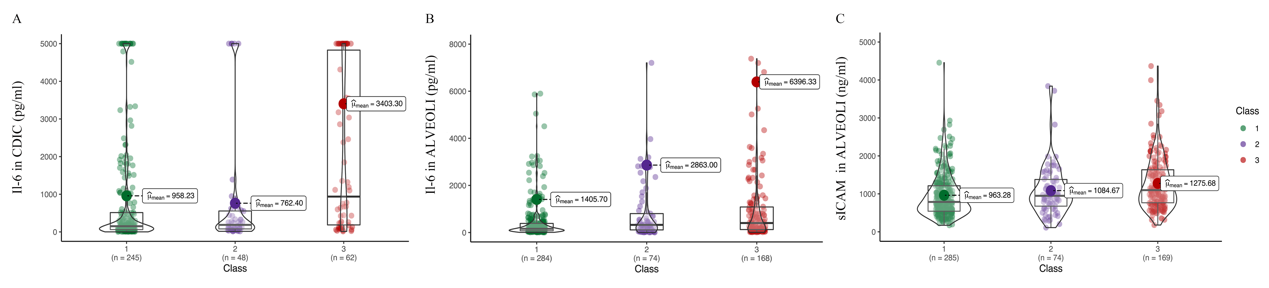
**

IL-6= ﻿Interleukin-6; sICAM= ﻿Soluble intercellular adhesion molecule-1.

**Figure S7: Derivation of** **longitudinal phenotypes for ARDS in CDIC including only the patients who remained on mechanical ventilation for more than 96 hours.**

**
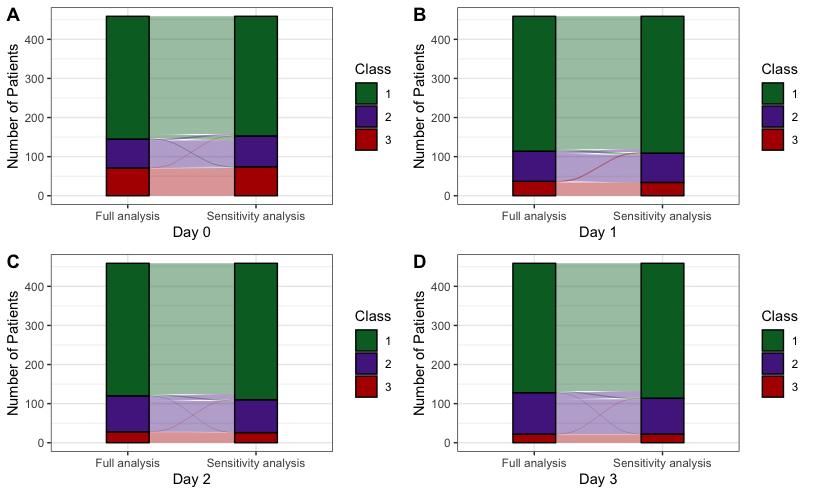
**

**Figure S8: Trajectories of mechanical power (A) and ventilatory ratio (B) in CDIC.**

**
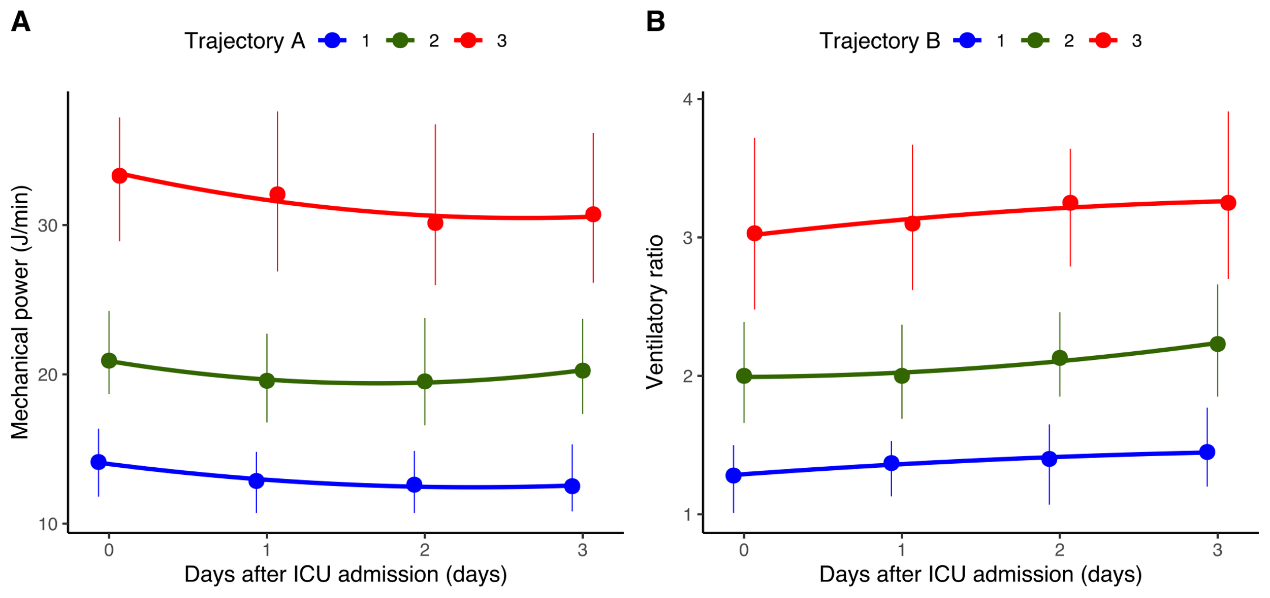
**

**Figure S9:** **﻿Comparison of dynamic changes of longitudinal phenotypes and trajectory analysis**

**
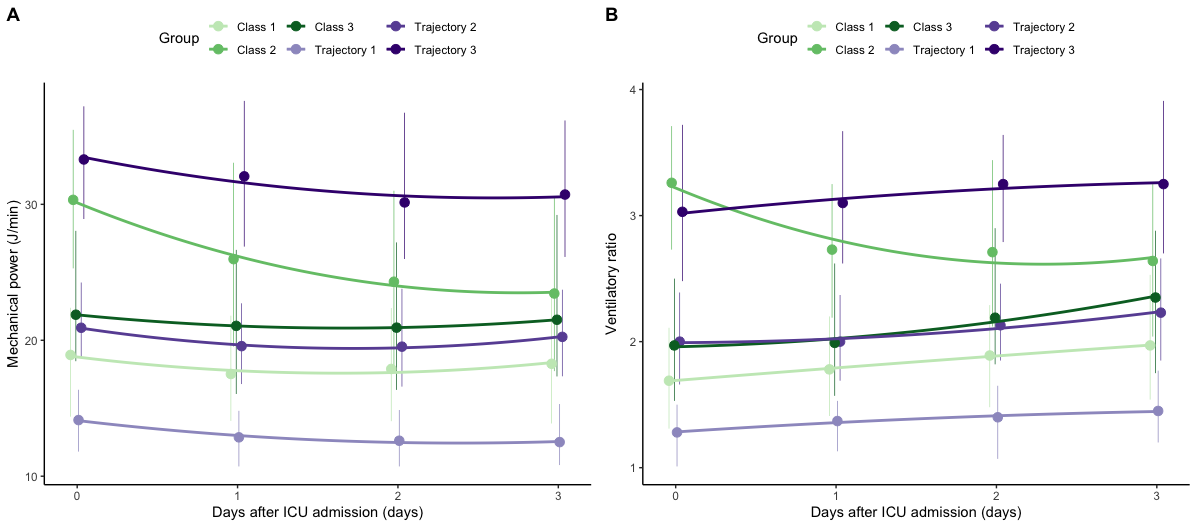
**

**Figure S10:** **ARDS state transition over days 0,1,2 and 3 in ARDSNet trials.**

**Figure S11: Clinical characteristics of the three longitudinal phenotypes in ALVEOLI (PEEP was not included in the longitudinal latent class analysis).**

**
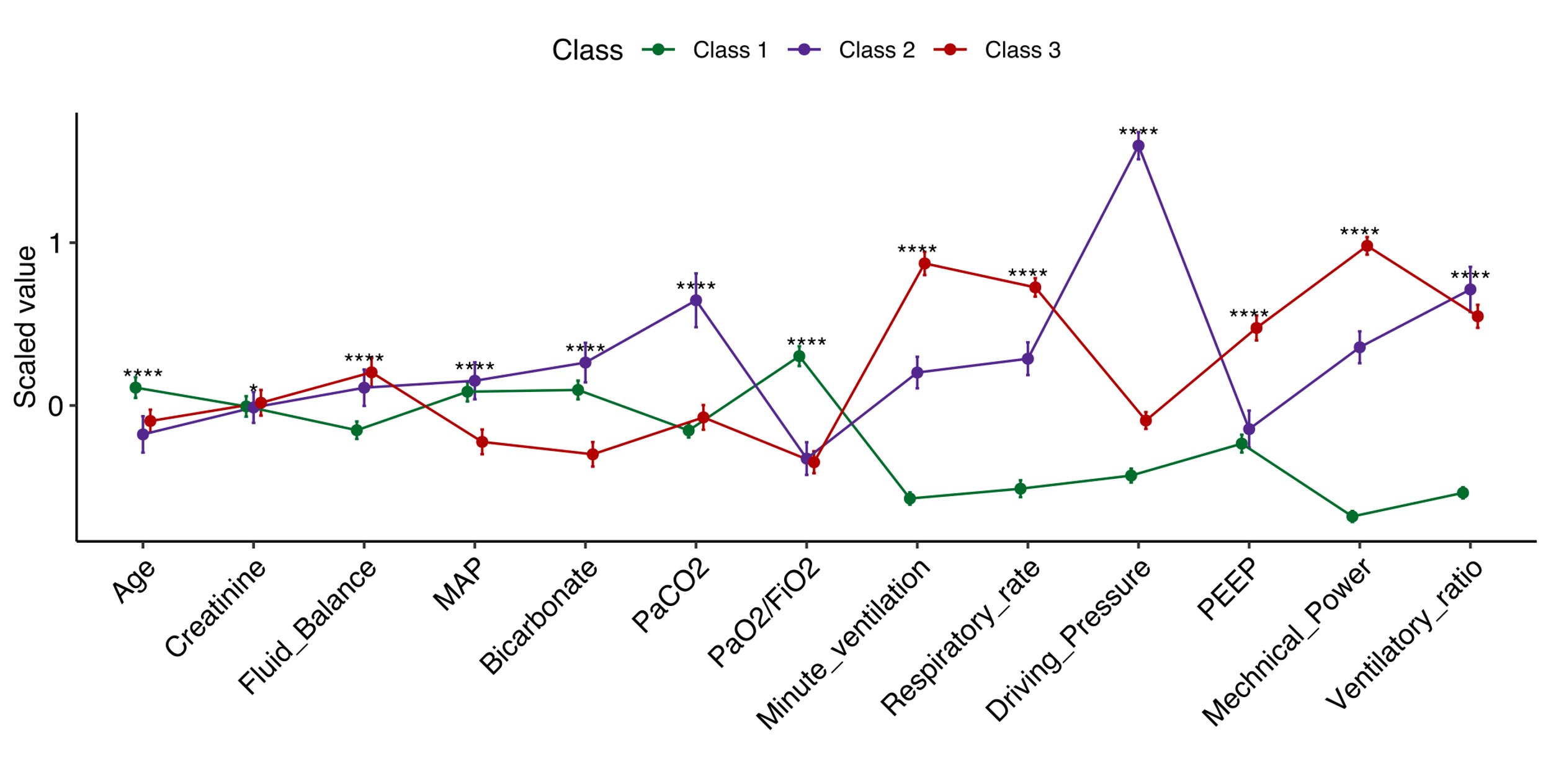
**

MAP=mean arterial blood pressure; PaCO_2_=partial pressure of Carbon Dioxide; PaO_2_=partial pressure of oxygen; FiO_2_= fraction of inspired oxygen; PEEP= positive end-expiratory pressure. ﻿****< 0.001.

**Figure S12:** **Clinical characteristics of the three longitudinal phenotypes in FACTT (fluid balance was not included in the longitudinal latent class analysis).**

**
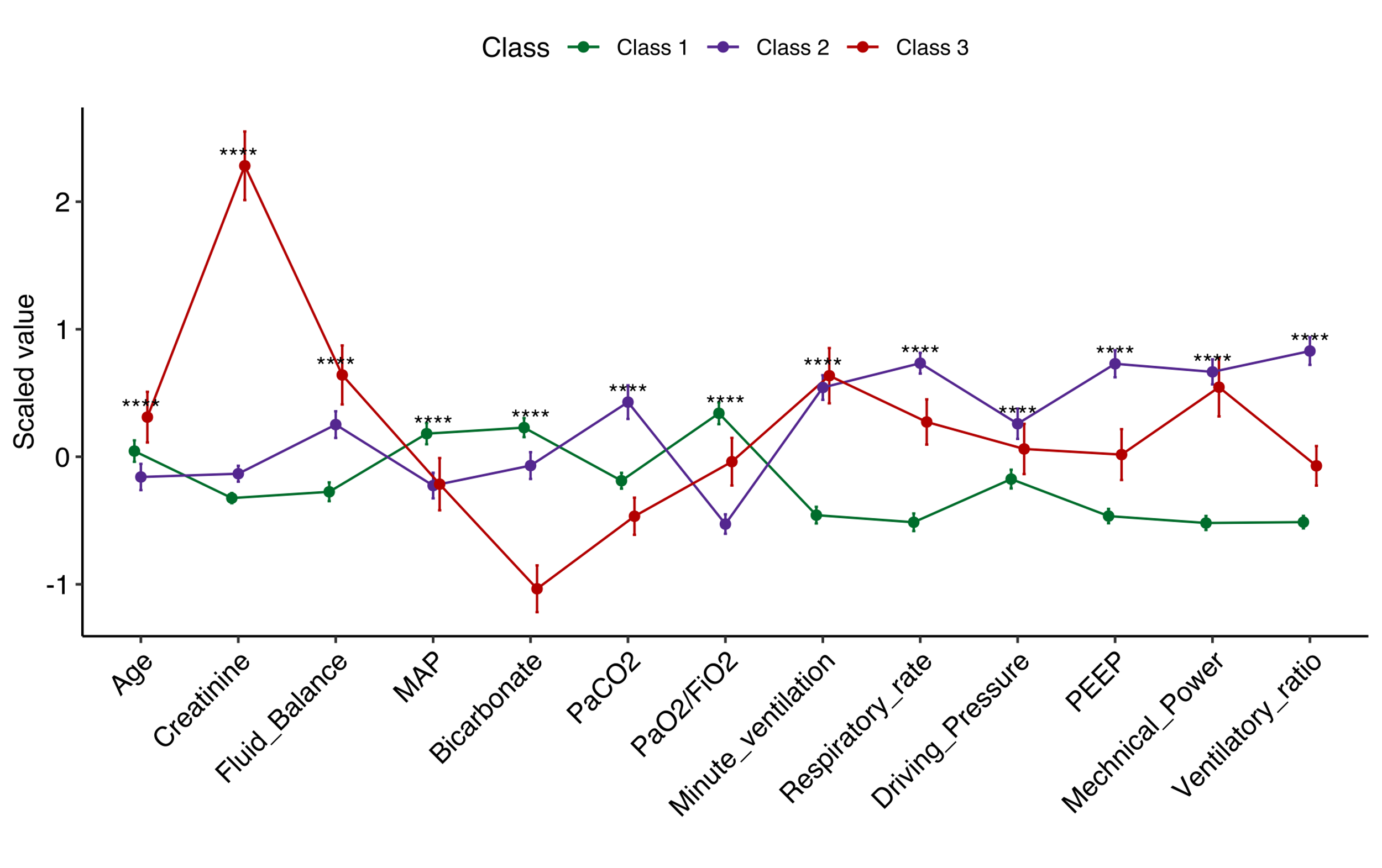
**

MAP=mean arterial blood pressure; PaCO_2_=partial pressure of Carbon Dioxide; PaO_2_=partial pressure of oxygen; FiO_2_= fraction of inspired oxygen; PEEP= positive end-expiratory pressure. ﻿****< 0.001.

**Figure S13: Clinical characteristics of the three longitudinal phenotypes in EDEN.**

**
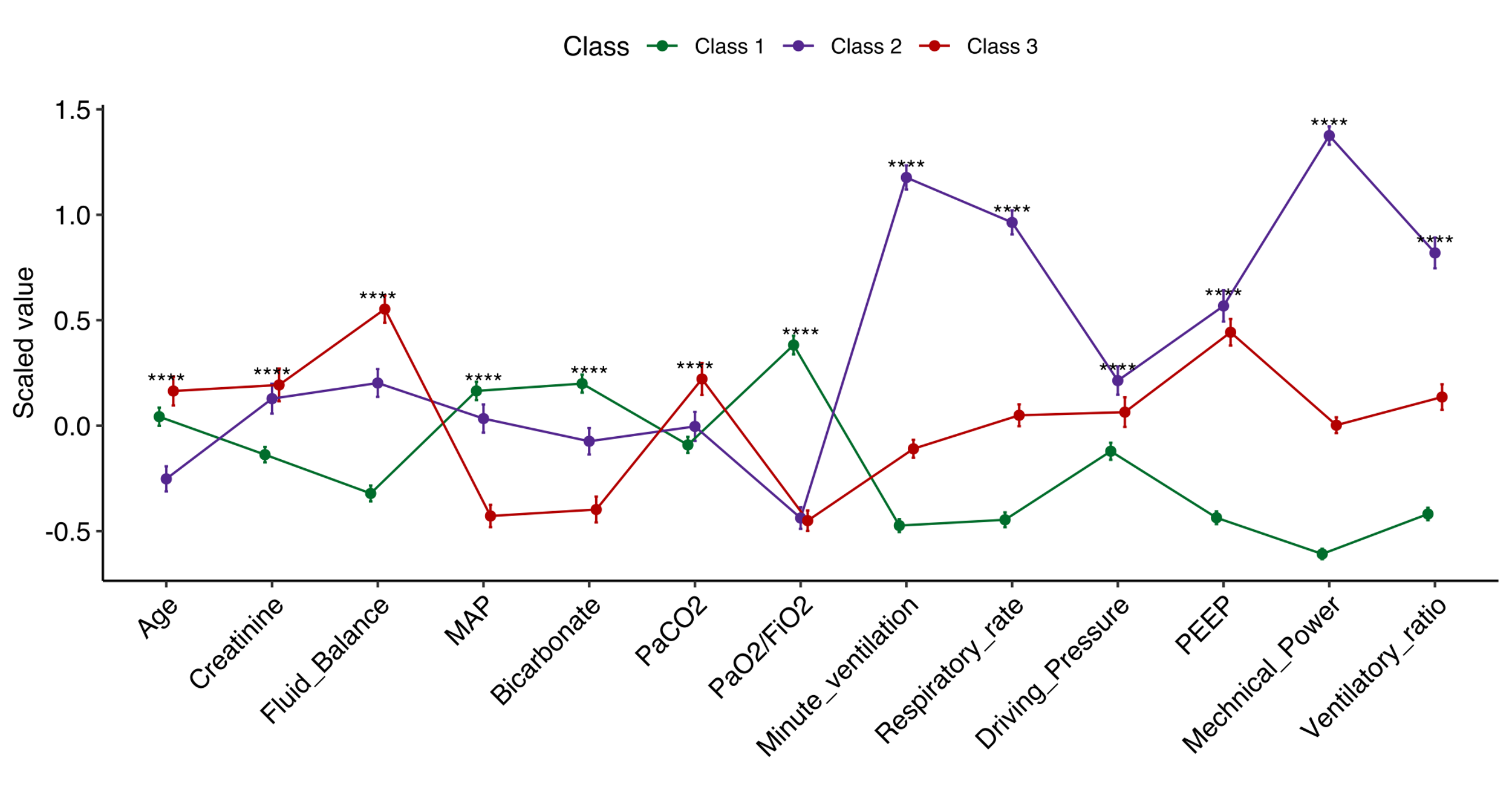
**

MAP=mean arterial blood pressure; PaCO_2_=partial pressure of Carbon Dioxide; PaO_2_=partial pressure of oxygen; FiO_2_= fraction of inspired oxygen; PEEP= positive end-expiratory pressure. ﻿****< 0.001.

**Figure S14: Clinical characteristics of the three longitudinal phenotypes in SAILS (Bicarbonate was not included in the longitudinal latent class analysis).**

**
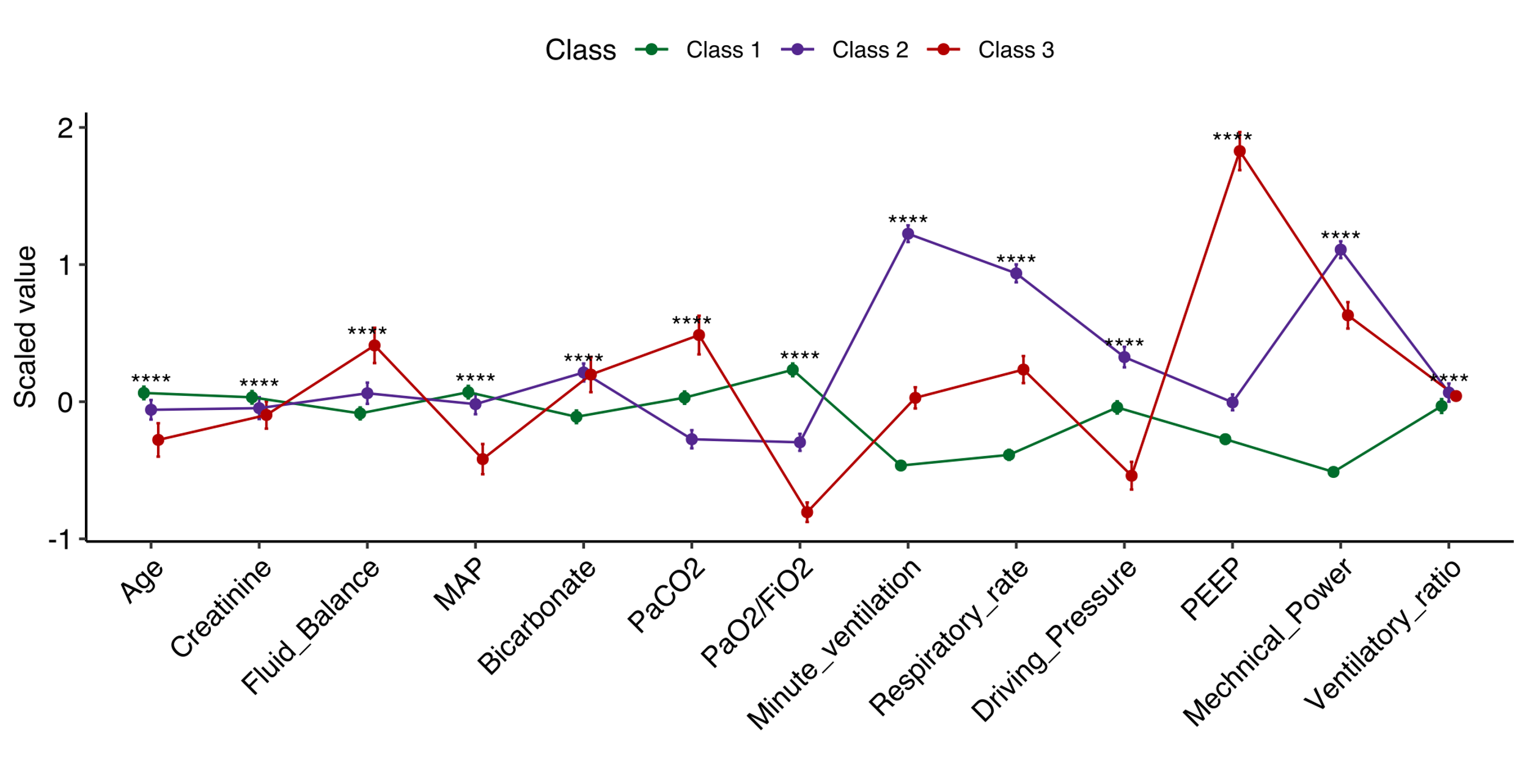
**

MAP=mean arterial blood pressure; PaCO_2_=partial pressure of Carbon Dioxide; PaO_2_=partial pressure of oxygen; FiO_2_= fraction of inspired oxygen; PEEP= positive end-expiratory pressure. ﻿****< 0.001.

**Figure S15:** **Heterogeneity of treatment effect to PEEP strategy within phenotypes on Day 0 in ALVEOLI Trial.**

**
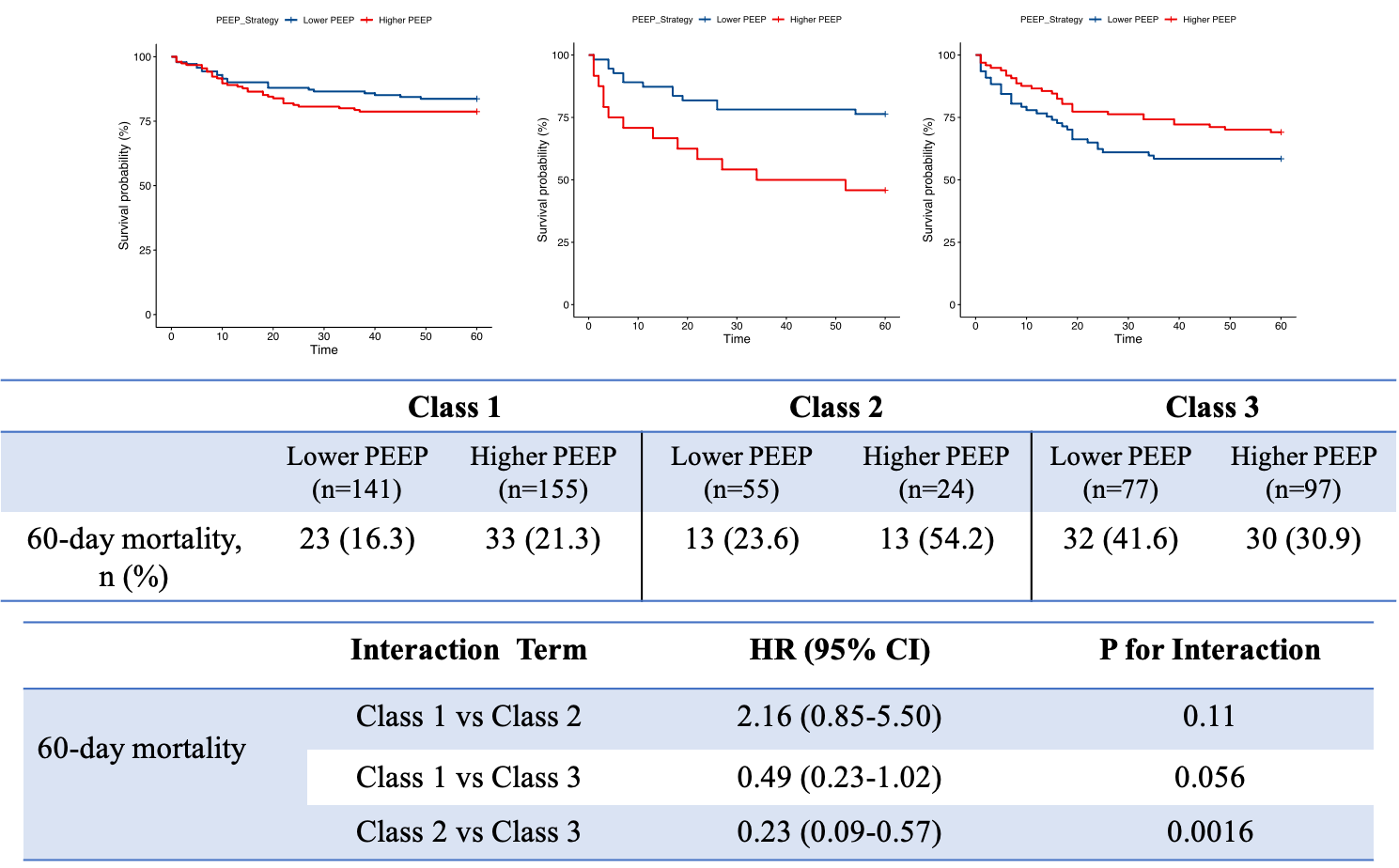
**

HR=Hazard ratio; PEEP= positive end-expiratory pressure.

**Figure S16: Heterogeneity of treatment effect to Fluid management strategy within phenotypes on Day 0 in FACTT Trial.**

**
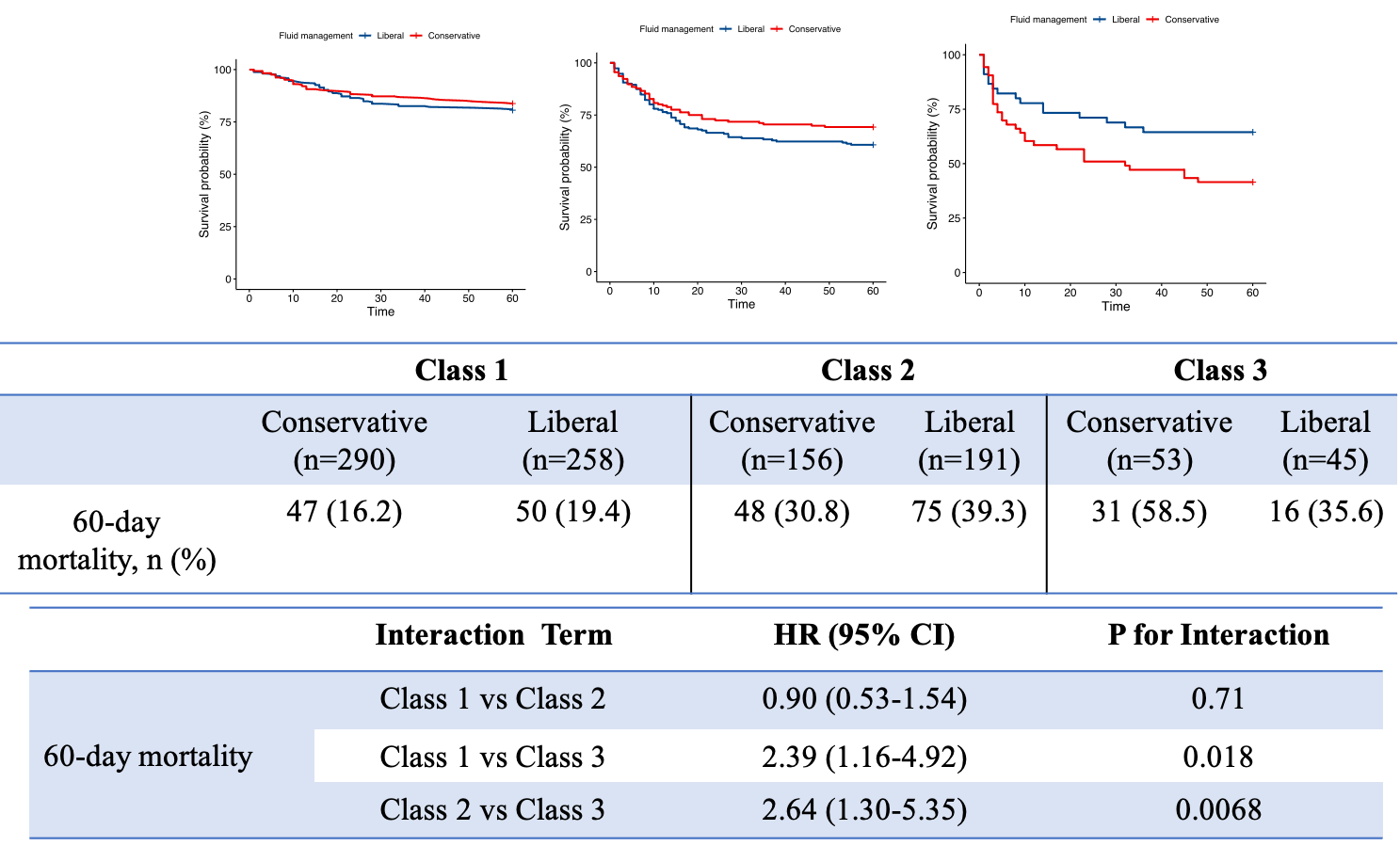
**

HR=Hazard ratio.

**Figure S17: Heterogeneity of treatment effect to Feeding strategy within phenotypes on Day 0 in EDEN Trial.**

**
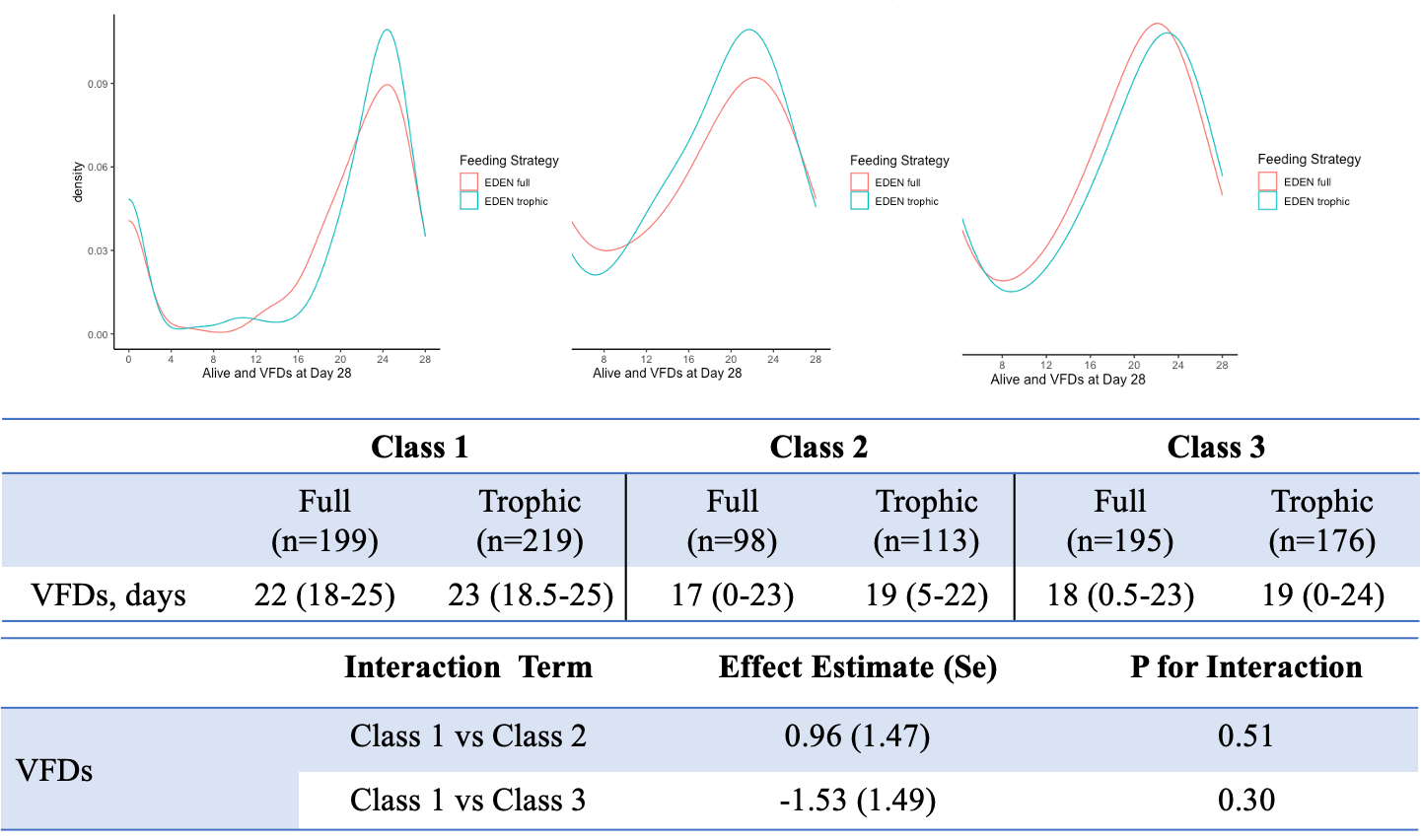
**

VFDs= ventilator free days; HR=Hazard ratio.

**Figure S18: Heterogeneity of treatment effect to Rosuvastatin within phenotypes on Day 0 in EDEN Trial.**

**
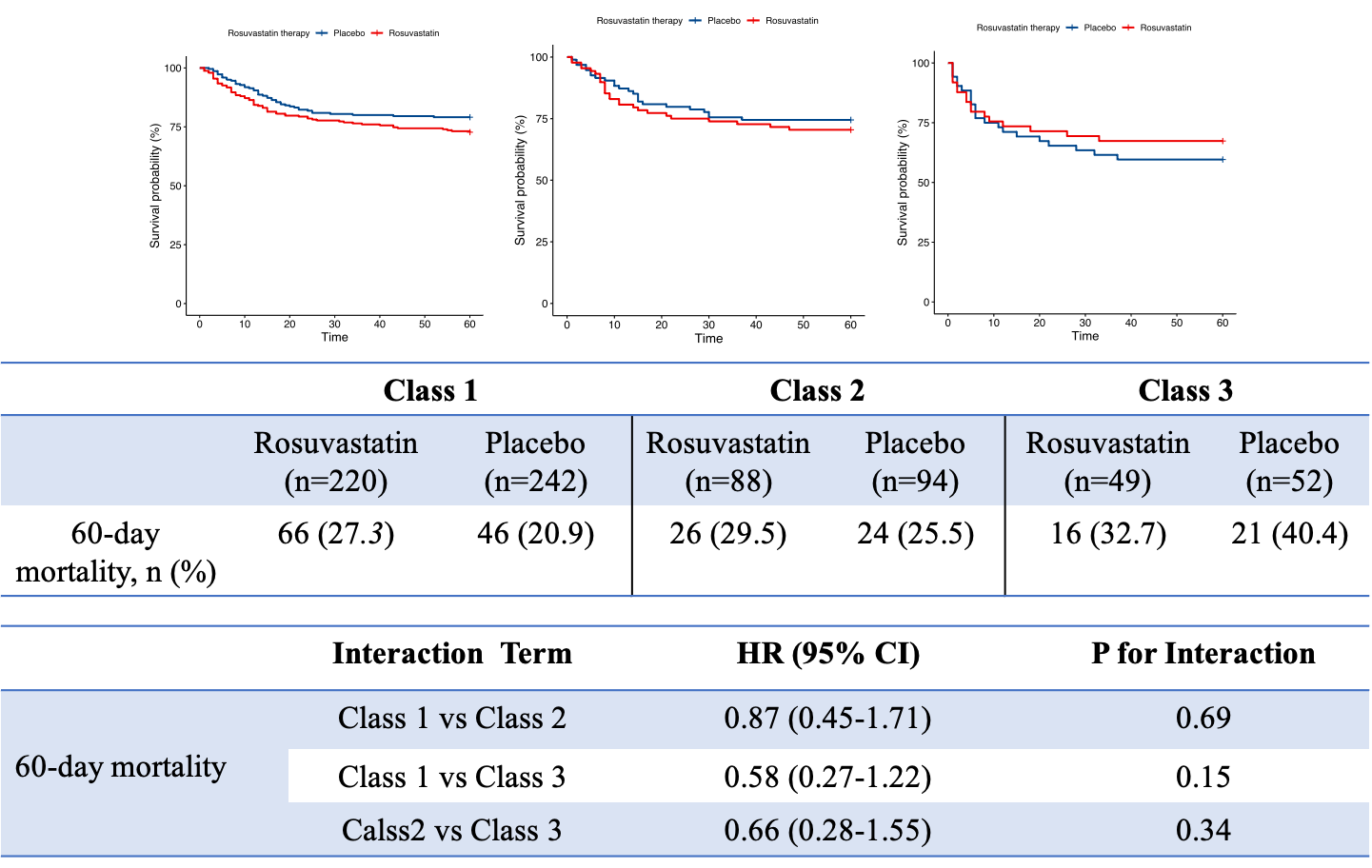
**

HR=Hazard ratio.

**Figure S19: The most important classifier variables on Day 0 from XGBoost (A) and GBM (B) to predict phenotypes on Day 0 in CDIC**

MAP=mean arterial blood pressure; PaCO_2_=partial pressure of Carbon Dioxide; PaO_2_=partial pressure of oxygen; FiO_2_= fraction of inspired oxygen; PEEP= positive end-expiratory pressure; BMI=body mass index; PBW=predicted body weight.

**Figure S20: The most important classifier variables on Day 0 from XGBoost (A) and GBM (B) to predict phenotypes on Day 2 in CDIC.**

**
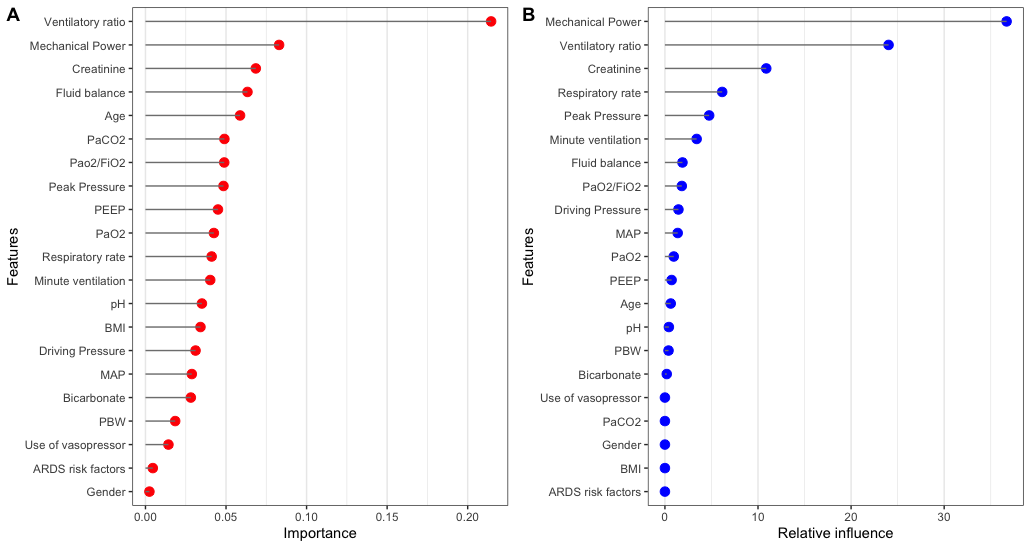
**

MAP=mean arterial blood pressure; PaCO_2_=partial pressure of Carbon Dioxide; PaO_2_=partial pressure of oxygen; FiO_2_= fraction of inspired oxygen; PEEP= positive end-expiratory pressure; BMI=body mass index; PBW=predicted body weight.
