# Supplementary material for: Longitudinal phenotypes in patients with acute respiratory distress syndrome: a multi-database study
Source: Crit Care. 2022 Nov 4;26:340. doi: 10.1186/s13054-022-04211-w (PMC9635207; doi:10.1186/s13054-022-04211-w)
Supplement: Supplementary file 2 — Additional file 2. R code for data analysis. [file 13054_2022_4211_MOESM2_ESM.pdf]

```
library(readxl)
library(tidyverse)
library(lubridate)
library(factoextra)
library(DataExplorer)
library(missForest)
library(cluster)
library(ggalluvial)
library(ggpubr)
library(cowplot)
library(gridExtra)
library(ggsci)
library(CBCgrps)
library(Publish)
library(tidyLPA)
library(data.table)
library(openxlsx)
library(survminer)
library(mice)
library(tableone)
library(survival)
```

#Phenotypes identification

```
dyn_ards<-new_ards%>%
  select(number,d1_map_min,d2_map_min,d3_map_min,d4_map_min,
         d1_temp_max,d2_temp_max,d3_temp_max,d4_temp_max,
         d1_hr_max,d2_hr_max,d3_hr_max,d4_hr_max,
         d1_ph,d2_ph,d3_ph,d4_ph,
         d1_pao2,d2_pao2,d3_pao2,d4_pao2,
         d1_pf,d2_pf,d3_pf,d4_pf,
         d1_paco2,d2_paco2,d3_paco2,d4_paco2,
         d1_bica,d2_bica,d3_bica,d4_bica,
         d1_lac,d2_lac,d3_lac,d4_lac,
         d1_wbc,d2_wbc,d3_wbc,d4_wbc,
         d1_hb,d2_hb,d3_hb,d4_hb,
         d1_plt,d2_plt,d3_plt,d4_plt,
         d1_lym,d2_lym,d3_lym,d4_lym,
         d1_pt,d2_pt,d3_pt,d4_pt,
         d1_inr,d2_inr,d3_inr,d4_inr,
         d1_aptt,d2_aptt,d3_aptt,d4_aptt,
         d1_fib,d2_fib,d3_fib,d4_fib,
         d1_ddimer,d2_ddimer,d3_ddimer,d4_ddimer,
         d1_alb,d2_alb,d3_alb,d4_alb,
         d1_tbil,d2_tbil,d3_tbil,d4_tbil,
```

```
           d1_alt,d2_alt,d3_alt,d4_alt,
           d1_ast,d2_ast,d3_ast,d4_ast,
           d1_cr,d2_cr,d3_cr,d4_cr,
           d1_bun,d2_bun,d3_bun,d4_bun,
           d1_hscrp,d2_hscrp,d3_hscrp,d4_hscrp,
           d1_input,d2_input,d3_input,d4_input,
           d1_output,d2_output,d3_output,d4_output,
           d1_fb,d2_fb,d3_fb,d4_fb,
           d1_mvrr,d2_mvrr,d3_mvrr,d4_mvrr,
           d1_vt,d2_vt,d3_vt,d4_vt,
           d1_mv,d2_mv,d3_mv,d4_mv,
           d1_peep,d2_peep,d3_peep,d4_peep,
           d1_peak_,d2_peak,d3_peak,d4_peak,
           d1_dp,d2_dp,d3_dp,d4_dp,
           d1_vr,d2_vr,d3_vr,d4_vr,
           d1_mp,d2_mp,d3_mp,d4_mp)%>%
set_names("number","mapmin_d1","mapmin_d2","mapmin_d3","mapmin_d4",
            "tempmax_d1","tempmax_d2","tempmax_d3","tempmax_d4",
            "hrmax_d1","hrmax_d2","hrmax_d3","hrmax_d4",
            "ph_d1","ph_d2","ph_d3","ph_d4",
            "pao2_d1","pao2_d2","pao2_d3","pao2_d4",
            "pf_d1","pf_d2","pf_d3","pf_d4",
            "paco2_d1","paco2_d2","paco2_d3","paco2_d4",
            "bica_d1","bica_d2","bica_d3","bica_d4",
            "lac_d1","lac_d2","lac_d3","lac_d4",
            "wbc_d1","wbc_d2","wbc_d3","wbc_d4",
            "hb_d1","hb_d2","hb_d3","hb_d4",
            "plt_d1","plt_d2","plt_d3","plt_d4",
            "lym_d1","lym_d2","lym_d3","lym_d4",
            "pt_d1","pt_d2","pt_d3","pt_d4",
            "inr_d1","inr_d2","inr_d3","inr_d4",
            "aptt_d1","aptt_d2","aptt_d3","aptt_d4",
            "fib_d1","fib_d2","fib_d3","fib_d4",
            "ddimer_d1","ddimer_d2","ddimer_d3","ddimer_d4",
            "alb_d1","alb_d2","alb_d3","alb_d4",
            "tbil_d1","tbil_d2","tbil_d3","tbil_d4",
            "alt_d1","alt_d2","alt_d3","alt_d4",
            "ast_d1","ast_d2","ast_d3","ast_d4",
            "cr_d1","cr_d2","cr_d3","cr_d4",
            "bun_d1","bun_d2","bun_d3","bun_d4",
            "hscrp_d1","hscrp_d2","hscrp_d3","hscrp_d4",
            "input_d1","input_d2","input_d3","input_d4",
            "output_d1","output_d2","output_d3","output_d4",
            "fb_d1","fb_d2","fb_d3","fb_d4",
```

```
                  "mvrr_d1","mvrr_d2","mvrr_d3","mvrr_d4",
                  "vt_d1","vt_d2","vt_d3","vt_d4",
                  "mv_d1","mv_d2","mv_d3","mv_d4",
                  "peep_d1","peep_d2","peep_d3","peep_d4",
                  "peak_d1","peak_d2","peak_d3","peak_d4",
                  "dp_d1","dp_d2","dp_d3","dp_d4",
                  "vr_d1","vr_d2","vr_d3","vr_d4",
                  "mp_d1","mp_d2","mp_d3","mp_d4")%>%
  pivot_longer(-number,names_to=c(".value","days"),names_sep = "_")%>%
  mutate(days=as.numeric(as.numeric(gsub(pattern = "d",replacement = "",days))))

lpa_ards<- dyn_ards %>%
  select(age,mapmin,hrmax,ph,pf,bica,lac,cr,fb,mv,peep,dp,vr,mp)
lpa_ards_1<-complete(mice(lpa_ards,method = "midastouch"))

lpa_ards_2<-lpa_ards_1%>%
  scale()%>%
  estimate_profiles(n_profiles=2:6, verbose=T)

PLAcriteria <- lpa_ards_2 %>% get_fit() %>%
  mutate(across(.cols = everything(),~round(.x,3))) %>%
  select(Classes,AIC,SABIC,Entropy,prob_min,prob_max,n_min,n_max,BLRT_p)%>%
  mutate(AIC=round(AIC),SABIC=round(SABIC))%>%
  ggtexttable(rows = NULL, theme = ttheme("mBlue")) %>%
  tab_add_title(text = "Best Number of Classes for FMM",
                face = "bold", padding = unit(1.5, "line"),
                just=c(-0.8,1))
#####n=3
lpa_ards_2<-lpa_ards_1%>%
  scale()%>%
  estimate_profiles(n_profiles=3, verbose=T)

dtClass_zd<-new_ards%>%
  bind_cols(Class=get_data(lpa_ards_2)$Class)%>%
  dplyr::filter(days%in%c(1,2,3,4))%>%
  as.data.frame()%>%
  dplyr::mutate(Class=if_else(
    Class==1,3,
    if_else(
      Class==2,2,
      if_else(Class==3,1,Class))))
```

####Transition of ARDS phenotypes across study days

```
dtClass_zd$days<-factor(dtClass_zd$days,levels = c(1,2,3,4),labels = c(0,1,2,3))
dtClass_zd$event<-factor(dtClass_zd$event,levels = c(0,1),labels = c("Survival","Death"))
dtClass_zd$Class<-factor(dtClass_zd$Class,levels = c(1,2,3))

plotAlluvia_1<-ggplot(dtClass_zd,aes(x = days, stratum = Class, alluvium = number,
                                     label = Class,fill=Class)) +
  geom_flow(stat = "alluvium",
            lode.guidance = "frontback",
  ) +
  geom_stratum() +scale_fill_manual(values = c("#006D2C","#54278F","#B30000"))+
  theme(legend.position = "none") +
  labs(title="ARDS State Transition Over Time",
       x="Days after ICU admission",
       ylab="Number of Patients")+theme_bw()

plotAlluvia_2<-ggplot(dtClass_zd,aes(x = days, stratum = Class, alluvium = number,
                                                  label = Class,fill=Class)) +
  geom_flow(stat = "alluvium",
            lode.guidance = "frontback",
  ) +
  geom_stratum() +scale_fill_manual(values = c("#006D2C","#54278F","#B30000"))+
  theme(legend.position = NULL) +
  labs(title="ARDS State Transition Over Time",
       x="Days after ICU admission",
       ylab="Number of Patients")+theme_bw()+
  facet_wrap(~ event, scales = "free_y")

plotAlluvia_3<-ggplot(dtClass_zd,aes(x = days, stratum = Class, alluvium = number,
                                                    label = Class,fill=Class)) +
  geom_flow(stat = "alluvium",
            lode.guidance = "frontback",
  ) +
  geom_stratum() +scale_fill_manual(values = c("#006D2C","#FFFFFF","#FFFFFF"))+
  theme(legend.position = NULL) +
  labs(title="ARDS State Transition Over Time",
       x="Days after ICU admission",
       ylab="Number of Patients")+theme_bw()

plotAlluvia_4<-ggplot(dtClass_zd,aes(x = days, stratum = Class, alluvium = number,
                                                    label = Class,fill=Class)) +
  geom_flow(stat = "alluvium",
            lode.guidance = "frontback",
  ) +
  geom_stratum() +scale_fill_manual(values = c("#FFFFFF","#54278F","#FFFFFF"))+
```

```
  theme(legend.position = NULL) +
  labs(title="ARDS State Transition Over Time",
       x="Days after ICU admission",
       ylab="Number of Patients")+theme_bw()
str(dtClass_zd$Class)

plotAlluvia_5<-ggplot(dtClass_zd,aes(x = days, stratum = Class, alluvium = number,
                                      label = Class,fill=Class)) +
  geom_flow(stat = "alluvium",
            lode.guidance = "frontback",
  ) +
  geom_stratum() +scale_fill_manual(values = c("#FFFFFF","#FFFFFF","#B30000"))+
  theme(legend.position = NULL) +
  labs(title="ARDS State Transition Over Time",
       x="Days after ICU admission",
       ylab="Number of Patients")+theme_bw()

fig_2<-ggdraw()+
  draw_plot(plotAlluvia_1, 0,0, 0.3, 0.45)+
  draw_plot(plotAlluvia_2, 0.3,0, 0.4, 0.45)+
  draw_plot(PCA2, 0.6,0.5, 0.4, 0.4)+
  draw_plot_label(c("A", "B"),
                  x=c(0,0.6), y=c(0.95,0.95), size = 10)

dev.copy(tiff,"fig2.tif", width=10, height=8, res=1200,units ="cm", compression = "lzw")


#####baselie characteristics between groups
tab2_ards<-multigrps(dtClass_zd[dtClass_zd$days==1,],gvar = "Class",varlist =
vars<-c("age","gender","height","pbw","weight","imv_dur",
          "ecmo","hypertension","diabetes","chf","CAD","CKD","cirrhosis","he","COPD",
          "tb","solid_tumor","cbd","hematopathy","ctd","immunosuppression",
          "icu_survival","hos_survival","time","sofa_d1","apacheii_d1",
          "vaso_d1", "mapmin","tempmax","hrmax","ph","pao2",
          "pf","paco2","bica","lac","wbc","hb",
          "plt","lym","pt","inr","aptt","fib",
          "ddimer","alb","tbil","alt",
          "ast","cr","bun","hscrp",
          "input","output","fb","mvrr","vt",
          "mv","peep","peak","dp","vr","mp","ARDS_risk_factors","vt_pbw","cstat","ela_sta
t","ela_dyn","bmi",
          "il6","CD3T","CD4T","CD8T","CD4/CD8","NK")

factorVars<-c("gender","hypertension","diabetes","chf","CAD","CKD","cirrhosis","he","COP
```

```
D",
                    "tb","solid_tumor","cbd","hematopathy","ctd","immunosuppression",
                    "icu_survival","hos_survival","ARDS_risk_factors","ecmo","vaso_d1")

table <- CreateTableOne(vars = vars, strata = "Class",data = dtClass_zd[dtClass_zd$day
s==1,], factorVars=factorVars,test = TRUE,includeNA = FALSE,
                              testApprox = chisq.test, argsApprox = list(correct = TRU
E), testExact = fisher.test,
                              argsExact = list(workspace = 2 * 10^5), testNormal = on
eway.test,
                              argsNormal = list(var.equal = TRUE),
                              testNonNormal = kruskal.test,
                              argsNonNormal = list(NULL), smd = TRUE)

print(table, nonnormal=c("age","height","pbw","weight","imv_dur","sofa_d1","apacheii_d1",
                              "mapmin","tempmax","hrmax","ph","pao2",
                              "pf","paco2","bica","lac","wbc","hb",
                              "plt","lym","pt","inr","aptt","fib",
                              "ddimer","alb","tbil","alt",
                              "ast","cr","bun","hscrp",
                              "input","output","fb","mvrr","vt",
                              "mv","peep","peak","dp","vr","mp","vt_pbw","cstat","ela_stat
","ela_dyn","bmi",
                              "il6","CD3T","CD4T","CD8T","CD4/CD8","NK"),quote = T
RUE, noSpaces = TRUE)
```

########SMD between phenotypes across days

```
diff<-dtClass_zd<-lpa_ards_1%>%
  as.data.frame()%>%
  select(age,mapmin,hrmax,ph,pf,paco2,bica,lac,ddimer,cr,fb,mvrr,mv,peep,dp,vr,mp)%>%
  set_names("Age","MAP","Heart rate","pH","PaO2/FiO2","PaCO2","Bicarbonate","Lactat
e","DDimer","Creatinine","Fluid Balance","Respiratpry Rate","Minute ventilation","PEEP",
"Driving pressure","Ventilatory ratio","Mechanical Power")%>%
  bind_cols(Class=get_data(lpa_ards_3)$Class)%>%
  bind_cols(Days=total$days)%>%
  dplyr::filter(Days%in%c(1,2,3,4))%>%
  as.data.frame()%>%
  dplyr::mutate(Class=if_else(
    Class==1,3,
    if_else(
      Class==2,2,
      if_else(Class==3,1,Class)
    )
  ))
```

```
diff_1<-diff%>%
   filter(Days==4)%>%
   select("Age":"Mechanical Power")%>%
   scale()
diff_2<-cbind(diff_1,diff[diff$Days==4,c(18,19)])%>%
   as.data.frame()

diff_class1<-subset(diff_2,diff_2$Class==1)
diff_class2<-subset(diff_2,diff_2$Class==2)
diff_class3<-subset(diff_2,diff_2$Class==3)

###difference between Class 1and Class 2
new_data1<-c(mean(diff_class1$Age),mean(diff_class1$MAP),mean(diff_class1$`Heart rate
`),mean(diff_class1$pH),mean(diff_class1$`PaO2/FiO2`),mean(diff_class1$PaCO2),mean(dif
f_class1$Bicarbonate),mean(diff_class1$Lactate),mean(diff_class1$DDimer),mean(diff_class
1$Creatinine),mean(diff_class1$`Fluid Balance`),mean(diff_class1$`Respiratpry Rate`),mea
n(diff_class1$`Minute ventilation`),mean(diff_class1$PEEP),mean(diff_class1$`Driving pre
ssure`),mean(diff_class1$`Ventilatory ratio`),mean(diff_class1$`Mechanical Power`))

new_data2<-c(mean(diff_class2$Age),mean(diff_class2$MAP),mean(diff_class2$`Heart rate
`),mean(diff_class2$pH),mean(diff_class2$`PaO2/FiO2`),mean(diff_class2$PaCO2), mean(d
iff_class2$Bicarbonate),mean(diff_class2$Lactate),mean(diff_class2$DDimer), mean(diff_cl
ass2$Creatinine),mean(diff_class2$`Fluid Balance`),mean(diff_class2$`Respiratpry Rate`),
mean(diff_class2$`Minute ventilation`),mean(diff_class2$PEEP),mean(diff_class2$`Driving
pressure`),mean(diff_class2$`Ventilatory ratio`),mean(diff_class2$`Mechanical Power`))

new_data3<-c(mean(diff_class3$Age),mean(diff_class3$MAP),mean(diff_class3$`Heart rate
`),mean(diff_class3$pH),mean(diff_class3$`PaO2/FiO2`),mean(diff_class3$PaCO2), mean(d
iff_class3$Bicarbonate),mean(diff_class3$Lactate),mean(diff_class3$DDimer), mean(diff_cl
ass3$Creatinine),mean(diff_class3$`Fluid Balance`),mean(diff_class3$`Respiratpry Rate`),
mean(diff_class3$`Minute ventilation`),mean(diff_class3$PEEP),mean(diff_class3$`Driving
pressure`),mean(diff_class3$`Ventilatory ratio`),mean(diff_class3$`Mechanical Power`))

new_data4_1<-as.data.frame(cbind(new_data1,new_data2))
new_data4_1$name<-c("Age","MAP","Heart rate","pH","PaO2/FiO2","PaCO2","Bicarbonat
e","Lactate","DDimer","Creatinine","Fluid Balance","Respiratpry Rate","Minute ventilation
","PEEP","Driving pressure","Ventilatory ratio","Mechanical Power")
arrange(new_data4_1, desc(new_data2))

new_data4_2<-as.data.frame(cbind(new_data1,new_data2))
new_data4_2$name<-c("Age","MAP","Heart rate","pH","PaO2/FiO2","PaCO2","Bicarbonat
e","Lactate","DDimer","Creatinine","Fluid Balance","Respiratpry Rate","Minute ventilation
","PEEP","Driving pressure","Ventilatory ratio","Mechanical Power")
```

```
new_data4_3<-as.data.frame(cbind(new_data1,new_data2))
new_data4_3$name<-c("Age","MAP","Heart rate","pH","PaO2/FiO2","PaCO2","Bicarbonat
e","Lactate","DDimer","Creatinine","Fluid Balance","Respiratpry Rate","Minute ventilation
","PEEP","Driving pressure","Ventilatory ratio","Mechanical Power")

new_data4_4<-as.data.frame(cbind(new_data1,new_data2))
new_data4_4$name<-c("Age","MAP","Heart rate","pH","PaO2/FiO2","PaCO2","Bicarbonat
e","Lactate","DDimer","Creatinine","Fluid Balance","Respiratpry Rate","Minute ventilation
","PEEP","Driving pressure","Ventilatory ratio","Mechanical Power")

new_data4_total<-rbind(new_data4_1,new_data4_2,new_data4_3,new_data4_4)%>%
  set_names("Class 1","Class 2","Variable","Days")
new_data4_total$Variable<-factor(new_data4_total$Variable,levels = c("Ventilatory ratio","
Mechanical Power","Respiratpry Rate","Minute ventilation","PaCO2","Bicarbonate","PEE
P","MAP","Driving pressure","Fluid Balance","Age","Creatinine","DDimer","Lactate","Hea
rt rate","PaO2/FiO2","pH"))

new_data4_total_1<-gather(new_data4_total,group,value,"Class 1":"Class 2")%>%
  set_names("Variable","Days","Phenotypes","value")

new_data4_total_1$Days<-factor(new_data4_total_1$Days)
new_data4_total_1$Phenotypes<-factor(new_data4_total_1$Phenotypes)

new_data4_total_1_1<-subset(new_data4_total_1,new_data4_total_1$Days==1)%>%
  filter(Variable%in%c("Ventilatory ratio","Mechanical Power","Respiratpry Rate","Minut
e ventilation","PaCO2","PEEP","Driving pressure","MAP","DDimer","Bicarbonate","Creati
nine","Lactate","Age","PaO2/FiO2","pH","Fluid Balance"))

new_data4_total_1_2<-subset(new_data4_total_1,new_data4_total_1$Days==2)%>%
  filter(Variable%in%c("Ventilatory ratio","Mechanical Power","Respiratpry Rate","Minut
e ventilation","PaCO2","PEEP","Driving pressure","MAP","DDimer","Bicarbonate","Creati
nine","Lactate","Age","PaO2/FiO2","pH","Fluid Balance"))


new_data4_total_1_3<-subset(new_data4_total_1,new_data4_total_1$Days==3)%>%
  filter(Variable%in%c("Ventilatory ratio","Mechanical Power","Respiratpry Rate","Minut
e ventilation","PaCO2","PEEP","Driving pressure","MAP","DDimer","Bicarbonate","Creati
nine","Lactate","Age","PaO2/FiO2","pH","Fluid Balance"))


new_data4_total_1_4<-subset(new_data4_total_1,new_data4_total_1$Days==4)%>%
  filter(Variable%in%c("Ventilatory ratio","Mechanical Power","Respiratpry Rate","Minut
e ventilation","PaCO2","PEEP","Driving pressure","MAP","DDimer","Bicarbonate","Creati
nine","Lactate","Age","PaO2/FiO2","pH","Fluid Balance"))
```

```
new_data4_total_2<-cbind(new_data4_total_1_1[,c(1,3,4)],new_data4_total_1_2[,c(3,4)],
                                new_data4_total_1_3[,c(3,4)],new_data4_total_1_4[c(3,
4)])%>%
  as.data.frame()%>%
  set_names("Variable","Phenotypes","Day1","Phenotypes2","Day2","Phenotypes3","Day3",
            "Phenotypes4","Day4")

ggplot(data = new_data4_total_2, mapping = aes(y= Day2, x = Variable))+coord_flip()
+geom_line(new_data4_total_2,mapping=aes(y= Day2, x = Variable,color=Phenotypes3,gr
oup=Phenotypes3),size=0.7)+theme_bw()+
  theme(axis.line = element_line(colour = "black"))+
  theme(panel.grid.major = element_blank(),panel.grid.minor = element_blank())+
  theme(legend.key = element_blank())+ylab("Standardized variable value")+
  theme(axis.title.x=element_text(size=12))+
  theme(legend.position = "top")+ theme(axis.title.y=element_text(size=14,colour = "wh
ite"))+theme(axis.text.y=element_text(size=12))+scale_color_manual(values = c("#BAE4B
3","#CBC9E2"))+geom_line(mapping=aes(y= Day2, x = Variable,color=Phenotypes2,grou
p=Phenotypes2), colour = c("#31A354","#31A354","#31A354","#31A354","#31A354","#3
1A354","#31A354","#31A354","#31A354","#31A354","#31A354","#31A354","#31A354","
#31A354","#31A354","#31A354","#756BB1","#756BB1","#756BB1","#756BB1","#756BB
1","#756BB1","#756BB1","#756BB1","#756BB1","#756BB1","#756BB1","#756BB1","#75
6BB1","#756BB1","#756BB1","#756BB1"),size=0.7)+ geom_line(mapping=aes(y= Day3,
x = Variable,color=Phenotypes3,group=Phenotypes3), colour = c("#74C476","#74C476","
#74C476","#74C476","#74C476","#74C476","#74C476","#74C476","#74C476","#74C476",
"#74C476","#74C476","#74C476","#74C476","#74C476","#74C476","#9E9AC8","#9E9AC
8","#9E9AC8","#9E9AC8","#9E9AC8","#9E9AC8","#9E9AC8","#9E9AC8","#9E9AC8","#
9E9AC8","#9E9AC8","#9E9AC8","#9E9AC8","#9E9AC8","#9E9AC8","#9E9AC8"),size=0.
7)+ geom_line (mapping=aes(y= Day4, x = Variable,color=Phenotypes4,group=Phenotyp
es4), colour = c("#BAE4B3","#BAE4B3","#BAE4B3","#BAE4B3","#BAE4B3","#BAE4B
3","#BAE4B3","#BAE4B3","#BAE4B3","#BAE4B3","#BAE4B3","#BAE4B3","#BAE4B3",
"#BAE4B3","#BAE4B3","#BAE4B3","#CBC9E2","#CBC9E2","#CBC9E2","#CBC9E2","#
CBC9E2","#CBC9E2","#CBC9E2","#CBC9E2","#CBC9E2","#CBC9E2","#CBC9E2","#CB
C9E2","#CBC9E2","#CBC9E2","#CBC9E2","#CBC9E2"),size=0.7)
```

######comparision between phenotypes

```
color1<-c("#006D2C","#54278F","#B30000")
names(lca_group)[1]<-"Time-dependent phenotypes"
lca_group$`Time-dependent phenotypes`<-factor(lca_group$`Time-dependent phenotypes`,l
evels = c(1,2,3))
pd<-position_dodge(.2)
lca_p1<-ggplot(data = lca_group,aes(x=days,y=mp,group=`Time-dependent phenotypes`,co
lor=`Time-dependent phenotypes`))+
  geom_errorbar(data=lca_group,aes(ymin=mp_1,ymax=mp_2),width=.0,size=0.25,position
= pd)+geom_point(aes(color=`Time-dependent phenotypes`),size=3,position = pd)+
```

```
  geom_smooth(method = "lm",formula = y~poly(x,2),size=1,se=FALSE,position = pd)+
  theme(axis.title.y=element_text(size=15))+theme(axis.title.x =element_text(size=20))+
  theme(axis.text.x.bottom =element_text(size=13))+theme(axis.text.y=element_text(size=2
0))+theme_classic()+
  theme(legend.key = element_blank())+scale_color_manual(values = color1)+
  theme(legend.position = "top")+
  ylab("Mechanical power (J/min)")+xlab("Days after ICU admission (days)")


lca_p2<-ggplot(data = lca_group,aes(x=days,y=vr,group=`Time-dependent phenotypes`,col
or=`Time-dependent phenotypes`))+
  geom_errorbar(data=lca_group,aes(ymin=vr_1,ymax=vr_2),width=.0,size=0.25,position =
 pd)+geom_point(aes(color=`Time-dependent phenotypes`),size=3,position = pd)+
  geom_smooth(method = "lm",formula = y~poly(x,2),size=1,se=FALSE,position = pd)+
  theme(axis.title.y=element_text(size=15))+theme(axis.title.x =element_text(size=20))+
  theme(axis.text.x.bottom =element_text(size=13))+theme(axis.text.y=element_text(size=2
0))+theme_classic()+
  theme(legend.key = element_blank())+scale_color_manual(values = color1)+
  theme(legend.position = "null")+
  ylab("Ventilatory ratio")+xlab("Days after ICU admission (days)")

lca_p3<-ggplot(data = lca_group,aes(x=days,y=pf,group=`Time-dependent phenotypes`,col
or=`Time-dependent phenotypes`))+
  geom_errorbar(data=lca_group,aes(ymin=pf_1,ymax=pf_2),width=.0,size=0.25,position =
 pd)+geom_point(aes(color=`Time-dependent phenotypes`),size=3,position = pd)+
  geom_smooth(method = "lm",formula = y~poly(x,2),size=1,se=FALSE,position = pd)+
  theme(axis.title.y=element_text(size=15))+theme(axis.title.x =element_text(size=20))+
  theme(axis.text.x.bottom =element_text(size=13))+theme(axis.text.y=element_text(size=2
0))+theme_classic()+
  theme(legend.key = element_blank())+scale_color_manual(values = color1)+
  theme(legend.position = "null")+
  ylab("PaO2/FiO2 ratio (mmHg)")+xlab("Days after ICU admission (days)")

lca_p4<-ggplot(data = lca_group,aes(x=days,y=lac,group=`Time-dependent phenotypes`,co
lor=`Time-dependent phenotypes`))+
  geom_errorbar(data=lca_group,aes(ymin=lac_1,ymax=lac_2),width=.0,size=0.25,position
= pd)+geom_point(aes(color=`Time-dependent phenotypes`),size=3,position = pd)+
  geom_smooth(method = "lm",formula = y~poly(x,2),size=1,se=FALSE,position = pd)+
  theme(axis.title.y=element_text(size=15))+theme(axis.title.x =element_text(size=20))+
  theme(axis.text.x.bottom =element_text(size=13))+theme(axis.text.y=element_text(size=2
0))+theme_classic()+
  theme(legend.key = element_blank())+scale_color_manual(values = color1)+
  theme(legend.position = "null")+
  ylab("Serum lactate (mmol/L)")+xlab("Days after ICU admission (days)")
```

```
lca_p5<-ggplot(data = lca_group,aes(x=days,y=cr,group=`Time-dependent phenotypes`,col
or=`Time-dependent phenotypes`))+
  geom_errorbar(data=lca_group,aes(ymin=cr_1,ymax=cr_2),width=.0,size=0.25,position =
pd)+geom_point(aes(color=`Time-dependent phenotypes`),size=3,position = pd)+
  geom_smooth(method = "lm",formula = y~poly(x,2),size=1,se=FALSE,position = pd)+
  theme(axis.title.y=element_text(size=15))+theme(axis.title.x =element_text(size=20))+
  theme(axis.text.x.bottom =element_text(size=13))+theme(axis.text.y=element_text(size=2
0))+theme_classic()+
  theme(legend.key = element_blank())+scale_color_manual(values = color1)+
  theme(legend.position = "null")+
  ylab("Serum Creatinine (mmol/L)")+xlab("Days after ICU admission (days)")


lca_p6<-ggplot(data = lca_group,aes(x=days,y=bica,group=`Time-dependent phenotypes`,c
olor=`Time-dependent phenotypes`))+
  geom_errorbar(data=lca_group,aes(ymin=bica_1,ymax=bica_2),width=.0,size=0.25,positio
n = pd)+geom_point(aes(color=`Time-dependent phenotypes`),size=3,position = pd)+
  geom_smooth(method = "lm",formula = y~poly(x,2),size=1,se=FALSE,position = pd)+
  theme(axis.title.y=element_text(size=15))+theme(axis.title.x =element_text(size=20))+
  theme(axis.text.x.bottom =element_text(size=13))+theme(axis.text.y=element_text(size=2
0))+theme_classic()+
  theme(legend.key = element_blank())+scale_color_manual(values = color1)+
  theme(legend.position = "null")+
  ylab("Serum Bicarbonate (mmol/L)")+xlab("Days after ICU admission (days)")

lca_p7<-plot_grid(lca_p1,lca_p2,lca_p3,lca_p4,lca_p5,lca_p6,ncol=3,labels=LETTERS[1:6],
align=c("v"))

names(lca_group1)[1]<-"Time-dependent phenotypes"
lca_group1$`Time-dependent phenotypes`<-factor(lca_group1$`Time-dependent phenotypes
`,levels = c(1,2,3))

lca_f1<-ggplot(data = lca_group1,aes(x=days,y=dp,group=`Time-dependent phenotypes`,co
lor=`Time-dependent phenotypes`))+
  geom_errorbar(data=lca_group1,aes(ymin=dp_1,ymax=dp_2),width=.0,size=0.25,position
= pd)+geom_point(aes(color=`Time-dependent phenotypes`),size=3,position = pd)+
  geom_smooth(method = "lm",formula = y~poly(x,2),size=1,se=FALSE,position = pd)+
  theme(axis.title.y=element_text(size=15))+theme(axis.title.x =element_text(size=20))+
  theme(axis.text.x.bottom =element_text(size=13))+theme(axis.text.y=element_text(size=2
0))+theme_classic()+
  theme(legend.key = element_blank())+scale_color_manual(values = color1)+
  theme(legend.position = "top")+
  ylab("Driving pressure (cmH20)")+xlab("Days after ICU admission (days)")
```

```
lca_f2<-ggplot(data = lca_group1,aes(x=days,y=peep,group=`Time-dependent phenotypes`, color=`Time-dependent phenotypes`))+
  geom_errorbar(data=lca_group1,aes(ymin=peep_1,ymax=peep_2),width=.0,size=0.25,position = pd)+geom_point(aes(color=`Time-dependent phenotypes`),size=3,position = pd)+
  geom_smooth(method = "lm",formula = y~poly(x,2),size=1,se=FALSE,position = pd)+
  theme(axis.title.y=element_text(size=15))+theme(axis.title.x =element_text(size=20))+
  theme(axis.text.x.bottom =element_text(size=13))+theme(axis.text.y=element_text(size=20))+theme_classic()+
  theme(legend.key = element_blank())+scale_color_manual(values = color1)+
  theme(legend.position = "null")+
  ylab("PEEP (cmH20)")+xlab("Days after ICU admission (days)")


lca_f3<-ggplot(data = lca_group1,aes(x=days,y=rr,group=`Time-dependent phenotypes`,color=`Time-dependent phenotypes`))+
  geom_errorbar(data=lca_group1,aes(ymin=rr_1,ymax=rr_2),width=.0,size=0.25,position = pd)+geom_point(aes(color=`Time-dependent phenotypes`),size=3,position = pd)+
  geom_smooth(method = "lm",formula = y~poly(x,2),size=1,se=FALSE,position = pd)+
  theme(axis.title.y=element_text(size=15))+theme(axis.title.x =element_text(size=20))+
  theme(axis.text.x.bottom =element_text(size=13))+theme(axis.text.y=element_text(size=20))+theme_classic()+
  theme(legend.key = element_blank())+scale_color_manual(values = color1)+
  theme(legend.position = "null")+
  ylab(" Respiratory rate (breaths/min)")+xlab("Days after ICU admission (days)")


lca_f4<-plot_grid(lca_f1,lca_f2,lca_f3,ncol=3,labels=LETTERS[1:3],align=c("v"))


lca_f5<-ggplot(data = lca_group1,aes(x=days,y=ph,group=`Time-dependent phenotypes`,color=`Time-dependent phenotypes`))+
  geom_errorbar(data=lca_group1,aes(ymin=ph_1,ymax=ph_2),width=.0,size=0.25,position = pd)+geom_point(aes(color=`Time-dependent phenotypes`),size=3,position = pd)+
  geom_smooth(method = "lm",formula = y~poly(x,2),size=1,se=FALSE,position = pd)+
  theme(axis.title.y=element_text(size=15))+theme(axis.title.x =element_text(size=20))+
  theme(axis.text.x.bottom =element_text(size=13))+theme(axis.text.y=element_text(size=20))+theme_classic()+
  theme(legend.key = element_blank())+scale_color_manual(values = color1)+
  theme(legend.position = "top")+
  ylab("pH")+xlab("Days after ICU admission (days)")
```

```
lca_f6<-ggplot(data = lca_group1,aes(x=days,y=pco2,group=`Time-dependent phenotypes`,
color=`Time-dependent phenotypes`))+
  geom_errorbar(data=lca_group1,aes(ymin=pco2_1,ymax=pco2_2),width=.0,size=0.25,posi
tion = pd)+geom_point(aes(color=`Time-dependent phenotypes`),size=3,position = pd)+
  geom_smooth(method = "lm",formula = y~poly(x,2),size=1,se=FALSE,position = pd)+
  theme(axis.title.y=element_text(size=15))+theme(axis.title.x =element_text(size=20))+
  theme(axis.text.x.bottom =element_text(size=13))+theme(axis.text.y=element_text(size=2
0))+theme_classic()+
  theme(legend.key = element_blank())+scale_color_manual(values = color1)+
  theme(legend.position = "null")+
  ylab(" PaCO2 (mmHg)")+xlab("Days after ICU admission (days)")

lca_f7<-ggplot(data = lca_group1,aes(x=days,y=plt,group=`Time-dependent phenotypes`,co
lor=`Time-dependent phenotypes`))+
  geom_errorbar(data=lca_group1,aes(ymin=plt_1,ymax=plt_2),width=.0,size=0.25,position
= pd)+geom_point(aes(color=`Time-dependent phenotypes`),size=3,position = pd)+
  geom_smooth(method = "lm",formula = y~poly(x,2),size=1,se=FALSE,position = pd)+
  theme(axis.title.y=element_text(size=15))+theme(axis.title.x =element_text(size=20))+
  theme(axis.text.x.bottom =element_text(size=13))+theme(axis.text.y=element_text(size=2
0))+theme_classic()+
  theme(legend.key = element_blank())+scale_color_manual(values = color1)+
  theme(legend.position = "null")+
  ylab("Platelets (*10^9/L)")+xlab("Days after ICU admission (days)")


lca_f8<-ggplot(data = lca_group1,aes(x=days,y=ddimer,group=`Time-dependent phenotype
s`,color=`Time-dependent phenotypes`))+
  geom_errorbar(data=lca_group1,aes(ymin=ddimer_1,ymax=ddimer_2),width=.0,size=0.25,
position = pd)+geom_point(aes(color=`Time-dependent phenotypes`),size=3,position = pd)
+
  geom_smooth(method = "lm",formula = y~poly(x,2),size=1,se=FALSE,position = pd)+
  theme(axis.title.y=element_text(size=15))+theme(axis.title.x =element_text(size=20))+
  theme(axis.text.x.bottom =element_text(size=13))+theme(axis.text.y=element_text(size=2
0))+theme_classic()+
  theme(legend.key = element_blank())+scale_color_manual(values = color1)+
  theme(legend.position = "null")+
  ylab("D-dimer (ng/ml)")+xlab("Days after ICU admission (days)")

lca_f9<-ggplot(data = lca_group1,aes(x=days,y=bun,group=`Time-dependent phenotypes`,c
olor=`Time-dependent phenotypes`))+
  geom_errorbar(data=lca_group1,aes(ymin=bun_1,ymax=bun_2),width=.0,size=0.25,positi
on = pd)+geom_point(aes(color=`Time-dependent phenotypes`),size=3,position = pd)+
  geom_smooth(method = "lm",formula = y~poly(x,2),size=1,se=FALSE,position = pd)+
```

```
  theme(axis.title.y=element_text(size=15))+theme(axis.title.x =element_text(size=20))+
  theme(axis.text.x.bottom =element_text(size=13))+theme(axis.text.y=element_text(size=2
0))+theme_classic()+
  theme(legend.key = element_blank())+scale_color_manual(values = color1)+
  theme(legend.position = "null")+
  ylab("Blood urea nitrogen(mg/dl)")+xlab("Days after ICU admission (days)")


lca_f10<-ggplot(data = lca_group1,aes(x=days,y=tbil,group=`Time-dependent phenotypes`,
color=`Time-dependent phenotypes`))+
  geom_errorbar(data=lca_group1,aes(ymin=tbil_1,ymax=tbil_2),width=.0,size=0.25,positio
n = pd)+geom_point(aes(color=`Time-dependent phenotypes`),size=3,position = pd)+
  geom_smooth(method = "lm",formula = y~poly(x,2),size=1,se=FALSE,position = pd)+
  theme(axis.title.y=element_text(size=15))+theme(axis.title.x =element_text(size=20))+
  theme(axis.text.x.bottom =element_text(size=13))+theme(axis.text.y=element_text(size=2
0))+theme_classic()+
  theme(legend.key = element_blank())+scale_color_manual(values = color1)+
  theme(legend.position = "null")+
  ylab("Bilirubin (umol/L)")+xlab("Days after ICU admission (days)")


lca_f11<-ggplot(data = lca_group1,aes(x=days,y=tbil,group=`Time-dependent phenotypes`,
color=`Time-dependent phenotypes`))+
  geom_errorbar(data=lca_group1,aes(ymin=tbil_1,ymax=tbil_2),width=.0,size=0.25,positio
n = pd)+geom_point(aes(color=`Time-dependent phenotypes`),size=3,position = pd)+
  geom_smooth(method = "lm",formula = y~poly(x,2),size=1,se=FALSE,position = pd)+
  theme(axis.title.y=element_text(size=15))+theme(axis.title.x =element_text(size=20))+
  theme(axis.text.x.bottom =element_text(size=13))+theme(axis.text.y=element_text(size=2
0))+theme_classic()+
  theme(legend.key = element_blank())+scale_color_manual(values = color1)+
  theme(legend.position = "null")+
  ylab("Bilirubin (umol/L)")+xlab("Days after ICU admission (days)")


lca_f12<-ggplot(data = lca_group1,aes(x=days,y=hscrp,group=`Time-dependent phenotype
s`,color=`Time-dependent phenotypes`))+
  geom_errorbar(data=lca_group1,aes(ymin=hscrp_1,ymax=hscrp_2),width=.0,size=0.25,po
sition = pd)+geom_point(aes(color=`Time-dependent phenotypes`),size=3,position = pd)+
  geom_smooth(method = "lm",formula = y~poly(x,2),size=1,se=FALSE,position = pd)+
  theme(axis.title.y=element_text(size=15))+theme(axis.title.x =element_text(size=20))+
  theme(axis.text.x.bottom =element_text(size=13))+theme(axis.text.y=element_text(size=2
0))+theme_classic()+
  theme(legend.key = element_blank())+scale_color_manual(values = color1)+
  theme(legend.position = "null")+
```

```
  ylab("Hs-CRP (mg/L)")+xlab("Days after ICU admission (days)")

lca_f13<-ggplot(data = lca_group1,aes(x=days,y=map,group=`Time-dependent phenotypes`,color=`Time-dependent phenotypes`))+
  geom_errorbar(data=lca_group1,aes(ymin=map_1,ymax=map_2),width=.0,size=0.25,position = pd)+geom_point(aes(color=`Time-dependent phenotypes`),size=3,position = pd)+
  geom_smooth(method = "lm",formula = y~poly(x,2),size=1,se=FALSE,position = pd)+
  theme(axis.title.y=element_text(size=15))+theme(axis.title.x =element_text(size=20))+
  theme(axis.text.x.bottom =element_text(size=13))+theme(axis.text.y=element_text(size=20))+theme_classic()+
  theme(legend.key = element_blank())+scale_color_manual(values = color1)+
  theme(legend.position = "null")+
  ylab("MAP (mmHg)")+xlab("Days after ICU admission (days)")


lca_f14<-plot_grid(lca_f5,lca_f6,lca_f7,lca_f8,
                   lca_f9,lca_f10,lca_f11,lca_f12,lca_f13,ncol=3,labels=LETTERS[1:12],align=c("v","h"))

######三个表型 D0 的 IL6 的比较（CDIC，alveoli）
dtClass_zd_infla<-dtClass_zd%>%
  filter(days==1)
inflam_zd$Class<-factor(inflam_zd$Class,levels = c(1,2,3))
lca_f15<-ggplot(data = inflam_zd,aes(x=Class,y=il6,group=Class,color=Class))+
  geom_errorbar(data=inflam_zd,aes(ymin=il6_1,ymax=il6_2),width=.0,size=0.25)+geom_point(aes(color=Class),size=3,position = pd)+
  theme(axis.title.y=element_text(size=15))+theme(axis.title.x =element_text(size=20))+
  theme(axis.text.x.bottom =element_text(size=13))+theme(axis.text.y=element_text(size=20))+theme_classic()+
  theme(legend.key = element_blank())+scale_color_manual(values = color1)+
  theme(legend.position = "null")+
  ylab("MAP (mmHg)")+xlab("Days after ICU admission (days)")

str(dtClass_zd_infla$Class)
ggbetweenstats(data=dtClass_zd_infla,x=Class,y=il6.x,caption=NULL,centrality.point.args = list(size = 5, color = color1),
               ylab = "IL-6 (pg/ml)")+ylim(0,5000)+scale_color_manual(values = color1)+
  theme_classic()


ggbetweenstats(data=dtClass_zd_infla,x=Class,y=hscrp,caption=NULL,centrality.point.args = list(size = 5, color = color1),
               ylab = "HsCRP (mg/L)")+ylim(0,450)+scale_color_manual(values = co
```

```
lor1)+
  theme_classic()

#####Phenotypes prediction
#####selection of phenotypes
xgb_traindata<-data.matrix(newards_model_2[,c(2:4,7:9,11:16,18,20:26,28)])
xgb_traindata1<-Matrix(xgb_traindata,sparse=T)
xgb_traindata2<-data.matrix(newards_model_2[,27])
xgb_traindata3<-list(data=xgb_traindata1,label=xgb_traindata2)
xgbtrain<-xgb.DMatrix(data=xgb_traindata3$data,label=xgb_traindata3$label)
##XGboost
xgb<-xgb.train(data=xgbtrain, max_depth=50, eta=0.1,objective='multi:softmax',nround=25,
               verbose=1, gamma=0,num_class=3)
str(newards_model_2)
importance_matrix<-xgb.importance(colnames(xgb_traindata3$data), model = xgb)
importance_matrix$Feature<-c("Ventilatory ratio","Respiratory rate","Mechanical Power","
pH","Creatinine","Age","PaCO2","MAP","Fluid balance","Pao2/FiO2","PEEP","Bicarbonat
e","Peak Pressure","PaO2","BMI","Minute ventilation","Driving Pressure","PBW","ARDS
risk factors",”Use of vasopressor","Gender")

ggplot(importance_matrix,aes(x=Importance,y=reorder(Feature,Importance)))+geom_point(si
ze=3,colour="red")+ theme_bw()+theme(panel.grid.major.y = element_blank(),legend.posit
ion = c(1,0.55), legend.justification = c(1,0.5))+ylab("Features")+
  geom_segment(aes(yend=Feature),xend=0,colour="grey50")

xgb.plot.importance(importance_matrix, rel_to_first = FALSE,
                    xlab = "Importance",left_margin = 9)

###GBM

gbm2<-gbm(Class~age+gender+pbw+ARDS_risk_factors+vaso_d1+mapmin+
            ph+pao2+pf+paco2+bica+cr+mvrr+fb+peep+mv+peak+dp+vr+mp+bmi
          ,distribution = 'gaussian',data = newards_model_2,n.trees = 292,shrinkage =
0.01)
best.iter <- gbm.perf(gbm2, method = "OOB")
print(best.iter)

Feature<-c("Mechanical Power","Respiratory rate","Ventilatory ratio","pH","Fluid balance
","Peak Pressure","ARDS risk factors","Creatinine","Driving Pressure","MAP","PaCO2","
BMI","Age","Pao2/FiO2","Use of vasopressor","PEEP","PaO2","Gender","PBW","Bicarbo
nate","Minute ventilation")
inf<-c(23.255,20.852,18.380,16.2394,3.518,3.494,2.620479,2.538,2.4538,2.028,1.775,0.880,
0.6665,0.4413,0.4088, 0.244,0.1998,0.000,0.000,0.000,0.000)
```

```
gbm<-cbind(Feature,inf)%>%
  as.data.frame()

gbm$inf<-as.numeric(gbm$inf)
ggplot(gbm,aes(x=inf,y=reorder(Feature,inf)))+geom_point(size=3,colour="blue")+
  theme_bw()+theme(panel.grid.major.y = element_blank(),legend.position = c(1,0.55),leg
end.justification = c(1,0.5))+ylab("Features")+geom_segment(aes(yend=Feature),xend=0,co
lour="grey50")+
  xlab("Relative influence")

############Logistical model

mn_res<-nnet::multinom(Class~vr+mp+mvrr+ph,data=newards_model_1)
newards_model_1$ph_1<-newards_model_1$ph*10
round(odds.ratio(mn_res),3)

mn_pred<-predict(mn_res,newards_model_1,type = 'prob')
mn_pred <- data.frame(mn_pred)
colnames(mn_pred)<-paste(colnames(mn_pred), "_pred_MN")

true_label<-dummies::dummy(newards_model_1$Class, sep = ".")
true_label<-data.frame(true_label)
colnames(true_label)<-gsub(".*?\\.", "", colnames(true_label))
colnames(true_label)<-paste(colnames(true_label), "_true")
final_df<-cbind(true_label,mn_pred)

roc_res<-multi_roc(final_df,force_diag=TRUE)
unlist(roc_res$AUC)
plot_roc_df<-plot_roc_data(roc_res)
str(plot_roc_df)
plot_roc_df$Group<-factor(plot_roc_df$Group)


summary(plot_roc_df)
plot_roc_df%>%
  as.data.frame()


auc_1<-ggplot(plot_roc_df, aes(x = 1-Specificity, y=Sensitivity)) +
  geom_path(aes(colour = Group), size=1.0) +
  geom_segment(aes(x = 0, y = 0, xend = 1, yend = 1),
               colour='grey', linetype = 'longdash') +
  theme_bw() +
```

```
    theme(plot.title = element_text(hjust = 0.5),
            legend.justification=c(1, 0), legend.position=c(.95, .05),
            legend.title=element_blank(),
            legend.background = element_rect(fill=NULL, size=0.5,
                                                              linetype="solid", colour ="black"))+scale
_color_manual(values = color1)

roc_auc_with_ci_res<-roc_auc_with_ci(final_df, conf= 0.95, type='basic', R = 100)
roc_auc_with_ci_res
```
